# A versatile cGAMP reporter reveals principles of cGAS activation by DNA damage and chromosome instability

**DOI:** 10.1038/s41556-026-02037-0

**Published:** 2026-08-21

**Authors:** Vivianne Lebrec, Alexandra Kanellou, Lauren R. Davies, Negar Afshar, Tomoya Kujirai, Robert J. Pickering, Vera Solntceva, Federico Tidu, Christian Zierhut

**Affiliations:** 1 Genome Stability and Innate Immunity Group, Division of Cell and Molecular Biology, The Institute of Cancer Research, London, UK; 2 Laboratory of Chromatin Structure and Function, Institute for Quantitative Biosciences, The University of Tokyo, Tokyo, Japan; 3 Laboratory for Transcription Structural Biology, RIKEN Center for Integrative Medical Sciences, Yokohama, Japan

**Keywords:** Innate immunity, DNA damage and repair, Chromosomes

## Abstract

cGAS is the primary innate immune DNA sensor. On binding DNA, cGAS generates cGAMP, ultimately driving inflammation. Although normally silenced on self-DNA, genotoxic stress can activate cGAS, proposed to be mediated by micronuclei, chromosome bridges and DNA:RNA hybrids. However, mechanistically, this is poorly understood due to a lack of sensitive and selective single-cell cGAS activation assays. Here we solve this with an improved cGAMP reporter for microscopy, flow cytometry and biochemical assays. Strikingly, we find that genotoxic stress-mediated cGAS activation is a rare event that is not driven by enrichment on micronuclei and occurs by mechanisms that vary in dependence on the genotoxic stress. Following chromosome mis-segregation, cGAS activation correlates with bridge association but, notably, ionizing radiation activates cGAS independently of bridges. Whereas simple DNA:RNA hybrids are inert, more complex structures such as R-loops activate cGAS. Our work revises the cGAS signalling framework and introduces a flexible tool to examine it.

## Abstract (web summary)

Lebrec et al. present a FRET-based cGAS reporter and show that cGAS activation by genotoxic stress is rare and uncoupled from micronuclei. The study implicates R-loop structures and subtypes of chromatin bridges as potential cGAS activators.

## Main

Most cells express components of the innate immune system^1^, including cGAS, a sensor of double-stranded DNA (dsDNA) that triggers inflammatory responses after detecting pathogen-derived DNA^2^. On binding DNA, cGAS generates cGAMP^3,4^, which is a ligand for STING^2^. cGAMP engagement leads to the formation of STING oligomers, which translocate to the Golgi apparatus and activate the kinases TBK1 and IKK. These promote nuclear enrichment of NF-κB and IRF3, ultimately driving changes in gene expression and the production of type I interferons (IFNs), including IFN-β^2^. IFN-β can then signal to the local environment through STAT1 and -2, resulting in induction of IFN-stimulated genes (ISGs)^2^.

Although initially thought to be exclusively cytoplasmic, cGAS is also present in the nucleus^5–8^, raising the question of how it avoids activation by self-DNA. We and others demonstrated that this is prevented by inhibitory association with nucleosomes^6,9–16^. However, during the genotoxic stress from DNA damage and chromosomal instability, cGAS can be activated independently of infection^11^. Although the underlying mechanisms are currently unclear, a predominant model involves micronuclei^17^. Formed by chromosome fragments, or mis-segregated chromosomes, micronuclei assemble nuclear envelopes that frequently rupture^17–19^, thereby allowing enrichment of the cytoplasmic cGAS pool^5,20–23^. Chromosome bridges, which also exhibit nuclear envelope defects, were also suggested to be cGAS activators^24^. However, both micronuclei and chromosome bridges are composed of chromatinized DNA^25–27^, which should keep cGAS inactive. Alternatively, cGAS activation during genome instability may result from products arising from R-loops^28^, structures formed when nascent transcripts anneal to the DNA template strand behind RNA polymerases, displacing the non-template strand^29,30^. DNA-repair factors can process R-loops to release DNA:RNA hybrids, which were suggested to activate cGAS^28^, building on earlier findings that artificial poly(dT:rA) hybrids can elicit limited cGAS responses^31^.

A major obstacle to understanding how cGAS is activated is the lack of robust techniques to detect cGAS activation in individual cells, which impedes correlating cGAS localization patterns with cGAS activation. Initial approaches for this employed messenger RNA sequencing of cells isolated by microscopy-assisted procedures^22^. This indicated a limited ISG induction in some micronucleated cells. However, ISG induction is an indirect cGAS activation measurement as it depends on IFN, which is diffusible. Another strategy involved the visualization of cGAS molecules that are in proximity to each other, which was proposed to reflect activation-associated dimerization^32^. However, cGAS molecules can be in proximity when inactivated by nucleosomes^10,15^, which implies that proximity does not necessarily indicate activation. Finally, recent studies used STING relocalization to the Golgi apparatus or nuclear IRF3 translocation to infer cGAS activation^33–35^. These studies indicated that micronuclear cGAS enrichment might not necessarily lead to its activation, but no other potential cGAS activation mechanisms were investigated. One study went further, questioning the literature supporting cGAS activation following genotoxic stress given that no obvious IRF3 relocalization was found after irradiation, replication stress or chromosome mis-segregation^34^. However, nuclear accumulation of IRF3 is an indirect measurement, based on a transient event, and can be initiated independently of cGAS^36^. Furthermore, because STING is degraded following its activation^37^, its reliability as a readout for cGAS activity is temporally limited. Finally, all these approaches depend on the presence of downstream cGAS signalling components and may often amplify downstream signalling^35^. Therefore, these approaches have limited ability to report on cGAS activation in a direct and reliable manner without changing the cellular signalling state.

As an alternative, recent work employed Förster resonance energy transfer (FRET) to determine the presence of intracellular cGAMP^38^. This approach provided a direct readout for cGAMP production, a step unique to cGAS and the most upstream in the pathway, but several issues limited the use of this construct. Here we present an improved cGAMP FRET reporter that functions in the presence of endogenous STING and is highly effective in microscopy and flow cytometry, and as a recombinant protein. We applied this reporter to investigate cGAS activation kinetics following genotoxic stress.

## Results

### An improved cGAMP reporter for microscopy, flow cytometry and biochemical assays

**Fig. 1:**
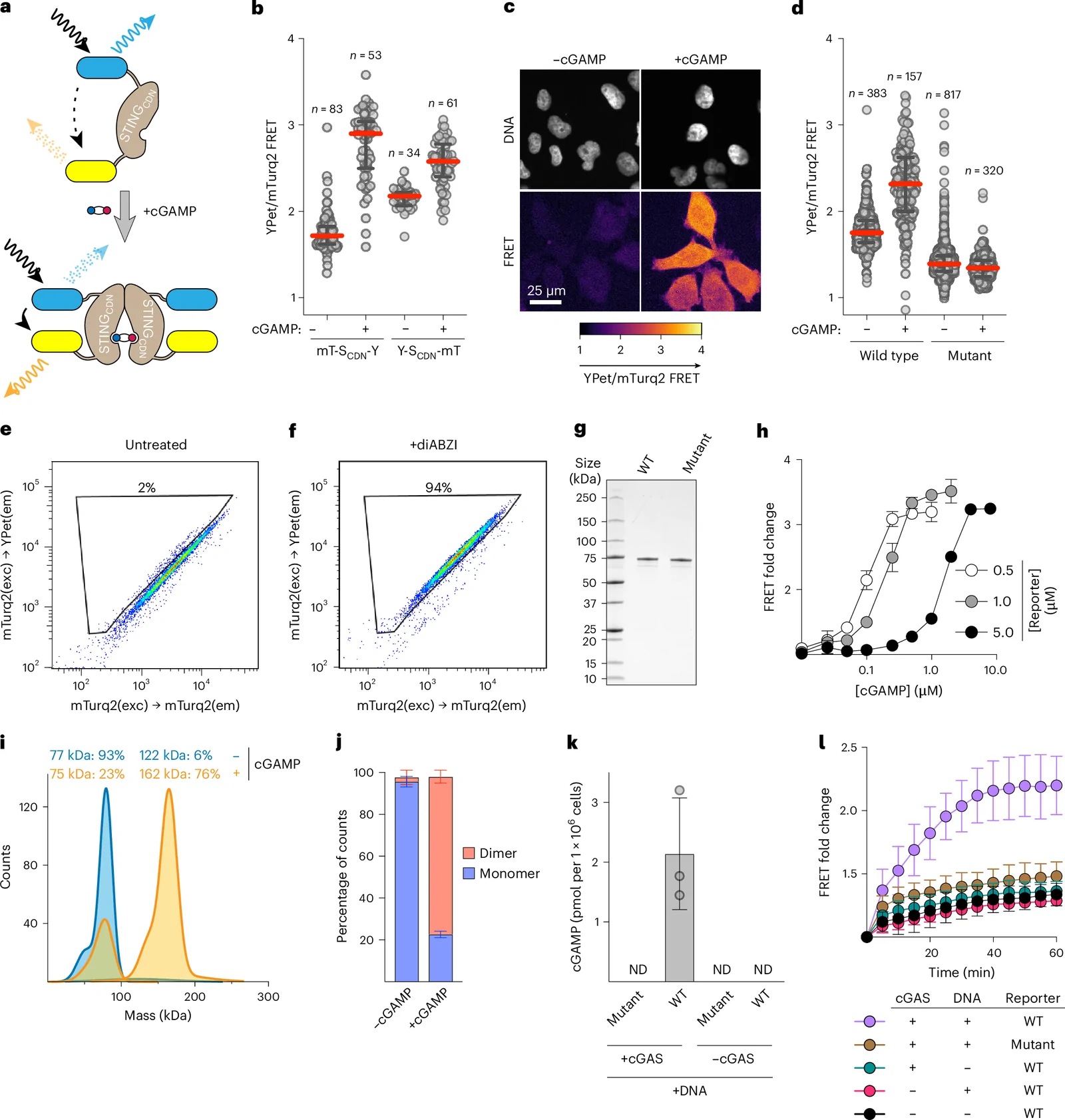
Design of an effective cGAMP reporter. **a**, Schematic of FRET-based cGAMP detection. **b**, Microscopy analysis of cGAS-disrupted HeLa cells electroporated with the indicated reporter constructs with or without cGAMP and analysed after 48 h. mT-S_CDN_-Y, mTurq2–STING_CDN_–YPet. Y-S_CDN_-mT, YPet–STING_CDN_–mTurq2. **c**,**d**, Microscopy analysis of cGAS-disrupted HeLa cells electroporated with mTurq2–STING_CDN_–YPet reporter constructs with or without cGAMP and analysed after 48 h. Representative images (**c**) and quantifications (**d**) are shown. **b**,**d**, Each dot represents one cell. Red lines, medians; black lines, interquartile ranges. **e**,**f**, Analysis of FRET in cGAMP reporter-expressing MDA-MB-231 cells that were either mock-treated (**e**) or treated with 1 μM diABZI for 16 h (**f**). The relative percentage of cells deemed cGAMP^+^ is shown above the gate; em, emission; exc, excitation. **b**–**f**, Representative data of three independent repeats. **g**, Gel stained with Coomassie brilliant blue showing purified wild-type (WT) and mutant reporters. **h**, Plate reader-based analysis of FRET generated by the indicated concentrations of reporter and cGAMP, normalized to unstimulated conditions. **i**,**j**, Mass photometry analysis of the oligomerization state of the reporter with and without cGAMP. Monomer predicted molecular weight, 76 kDa. **i**, Representative curves from one experiment. **k**, Measurement of cGAMP production by cells with or without cGAS that were transfected with DNA. Cell lysates were analysed for cGAMP using the improved reporter, either in its WT form or in its cGAMP-blind mutant form. ND, not detected. The standard curve for this experiment is in Extended Data Fig. 1h. **l**, In vitro reconstitution of cGAMP generation by cGAS using reporter FRET as readout. **g**,**j**,**k**,**l**, Averages and s.d. of three experiments are shown. Source numerical data and the unprocessed gel image are provided. Source data

**Extended Data Fig. 1:**
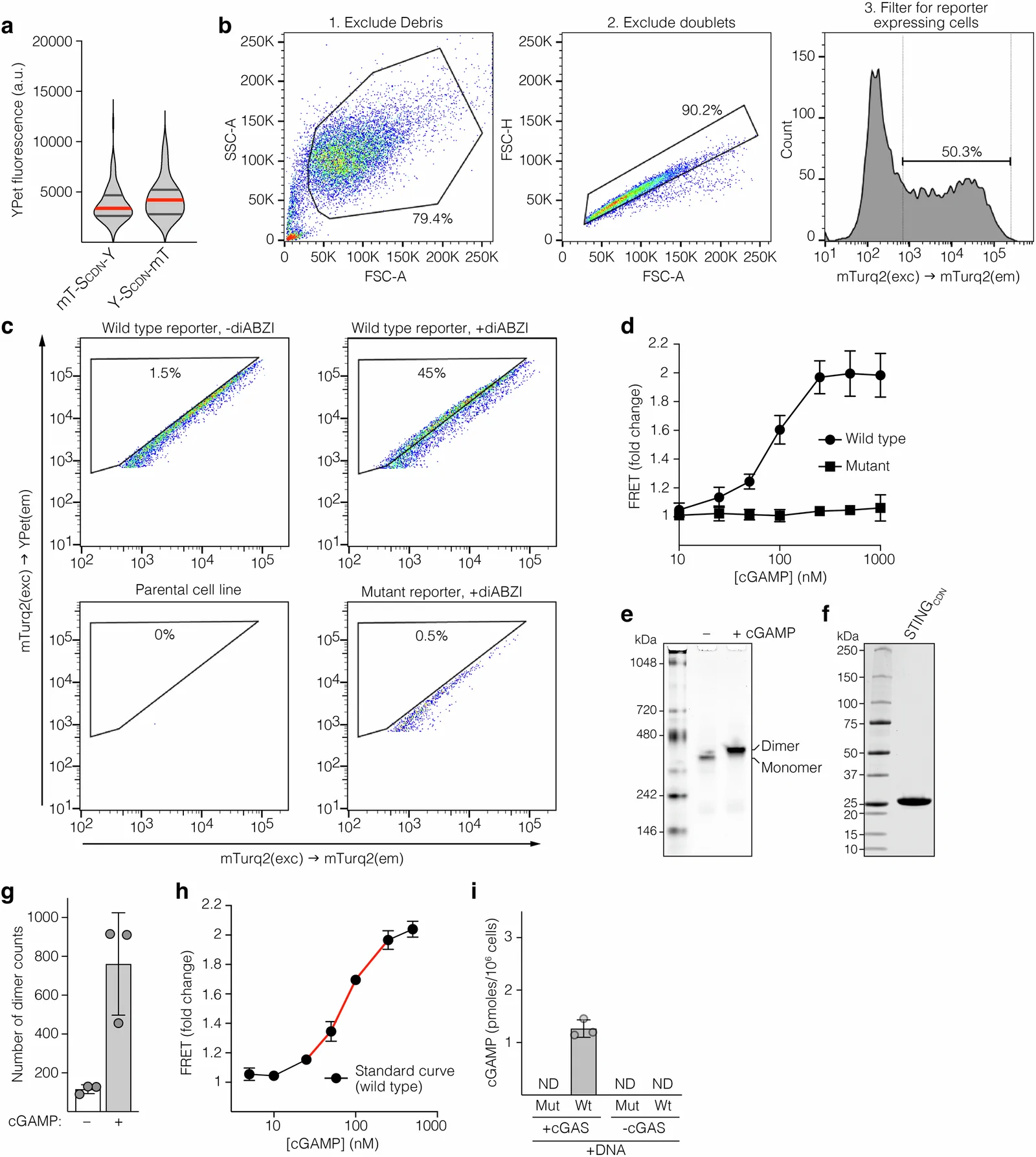
Flow cytometry and biochemical characterization of the cGAMP reporter. **a**, Reporter expression levels for the indicated constructs. Shown is one of three independent experiments (median and interquartile range shown). **b**, Gating strategy to measure FRET signal in HEK293FT cells transiently transfected with the cGAMP reporter. Cells were first gated on FSC-A and SSC-A, followed by singlet isolation by FSC-A vs FSC-H. Non-expressing cells were excluded based on mTurq2 fluorescence. **c**, Analysis of cGAMP-reporter FRET in flow cytometry. HEK293 cells were transfected with constructs promoting expression of the improved cGAMP reporter or its mutant cGAMP-blind version, and subsequently treated with 1 µM diABZI for 16 h. FRET-positive cells are shown in red, FRET-negative cells are shown in blue. Percentages of FRET positive cells indicated above the gate. Shown is one of two independent experiments. **d**, Plate reader-based analysis of FRET generated by the indicated concentrations of wild-type or mutant reporter and cGAMP, normalized to unstimulated conditions. Averages and standard deviation from three independent experiments are shown. **e**, Native gel electrophoresis of the cGAMP reporter pre-incubated with or without cGAMP, stained with Coomassie brilliant blue. Note that molecular weight markers do not necessarily indicate that proteins running in a similar region are of similar molecular weight, as separation depends on protein mass, shape and charge. Shown is one of two independent experiments. **f**, Coomassie brilliant blue-stained gel of purified mouse STING_CDN_. **g**, Mass photometry analysis of STING_CDN_ dimerization in dependence on cGAMP. Averages and standard deviation from three experiments are shown. Please note that STING_CDN_ is too small for the monomeric version to be detected. Therefore, we measured the number of dimer counts detected in the same amount of time for each sample. **h**, Standard curve for one of the experiments shown in Fig. 1k. Red lines indicate the confidence region used to determine cGAMP concentration in experimental samples. **i**, Measurement of cGAMP production by HeLa cells +/− cGAS that were transfected with DNA. Cell lysates were analysed for cGAMP using a commercial ELISA kit. Averages from three experiments (bar) and standard deviation are shown. ND, not detected. Source numerical data and unprocessed blots are provided.

To investigate the cGAS activation state of individual cells, we built on a previously published genetically encoded FRET-based reporter consisting of the cyclic dinucleotide binding domain (CDN) of STING sandwiched between cyan and orange fluorescent proteins^38^. Conformation changes dependent on cGAMP engagement led to increased FRET from the donor to the acceptor, used as a readout for the cGAS activation status. However, this reporter is not well-suited for microscopy, primarily due to its suboptimal FRET pair that has low spectral overlap and an acceptor emission spectrum that is not readily compatible with standard microscopy filters. Furthermore, this reporter did not seem functional in the presence of endogenous STING. To improve this approach, we used an optimized FRET pair, mTurquoise2 (mTurq2)^39^ and YPet^40^ (Fig. 1a), which we had previously used to investigate the DNA-damage checkpoint^41^ and is well-suited for microscopy. Using the mouse STING CDN for the cGAMP-binding region as previously described^38^, we prepared two versions, YPet–STING_CDN_–mTurq2 and mTurq2–STING_CDN_–YPet. When electroporated together with cGAMP, both constructs were similarly expressed but only the mTurq2–STING_CDN_–YPet construct showed a cGAMP-dependent ratiometric FRET shift of more than 2σ (Extended Data Fig. 1a and Fig. 1b,c). As expected, cGAMP did not increase FRET with a mutated version of the reporter (Y239S/T262A) that does not bind cGAMP^38^ (Fig. 1d). Given the superior FRET shift with mTurq2–STING_CDN_–YPet, we used this construct for subsequent experiments (hereafter referred to as cGAMP reporter).

To test whether this cGAMP reporter is suitable for flow cytometry, HEK293 cells, which lack endogenous cGAS, were transfected with cGAMP reporter plasmids. The cells were subsequently mock-treated or incubated with 1 μM of the STING agonist diABZI^42^ and analysed. A FRET shift was observed in treated cells expressing wild-type reporter but not those expressing mutant reporter (Extended Data Fig. 1b,c), demonstrating usability in flow cytometry. Similar results were obtained in reporter-expressing MDA-MB-231 breast cancer cells (Fig. 1e,f).

To investigate the mechanism underlying the cGAMP-induced FRET increase and characterize the reporter in isolation, we purified wild-type and mutant reporters expressed in *Escherichia coli* (Fig. 1g). Only the wild-type reporter exhibited a concentration-dependent FRET shift across broad ranges of cGAMP and reporter concentrations (Fig. 1h and Extended Data Fig. 1d). As expected, because signal depends on the fraction of reporter molecules bound to cGAMP, the linear detection range scaled with reporter concentration (Fig. 1h), enabling detection of a wide range of cGAMP concentrations by adjusting reporter levels. Mass photometry showed that the unbound reporter is predominantly monomeric, whereas the cGAMP-bound reporter is predominantly dimeric and does not oligomerize (Fig. 1i,j). Consistent with these data, a single band that shifted following cGAMP binding rather than forming a smear was observed following native gel electrophoresis, confirming the absence of oligomers (Extended Data Fig. 1e). The complete and stable shift during electrophoresis further suggests tight cGAMP binding and a potentially low off-rate. Similar cGAMP-induced dimerization was observed for the CDN lacking mTurq2 and YPet (Extended Data Fig. 1f,g and Discussion). Together, these results show that the cGAMP-bound reporter is primarily dimeric and that it is functional in cells and as a purified protein.

Typically, cGAMP production is measured in cell lysates using mass spectrometry or commercial ELISA kits. To determine whether our reporter can be used in a similar manner, we prepared lysates from DNA-transfected or mock-treated wild-type and cGAS-disrupted HeLa cells. We detected cGAMP only in lysates from DNA-transfected, cGAS-expressing cells (Fig. 1k and Extended Data Fig. 1h), at concentrations comparable to those estimated by ELISA (Extended Data Fig. 1i). As expected, no response was observed with mutant reporter (Fig. 1k). Overall, this indicates that the reporter can be used to determine the presence of cGAMP in cell lysates.

Finally, we tested whether our reporter could detect cGAMP production in real-time biochemical assays. Mixing recombinant cGAS with ATP and GTP caused a DNA-dependent increase in FRET over time with wild-type (but not mutant) reporter (Fig. 1l) demonstrating its utility in biochemical assays. We conclude that our cGAMP reporter is a versatile tool for detecting cGAMP in vivo and in vitro.

### The improved cGAMP reporter is highly sensitive for detecting cGAS activation in cells

**Fig. 2:**
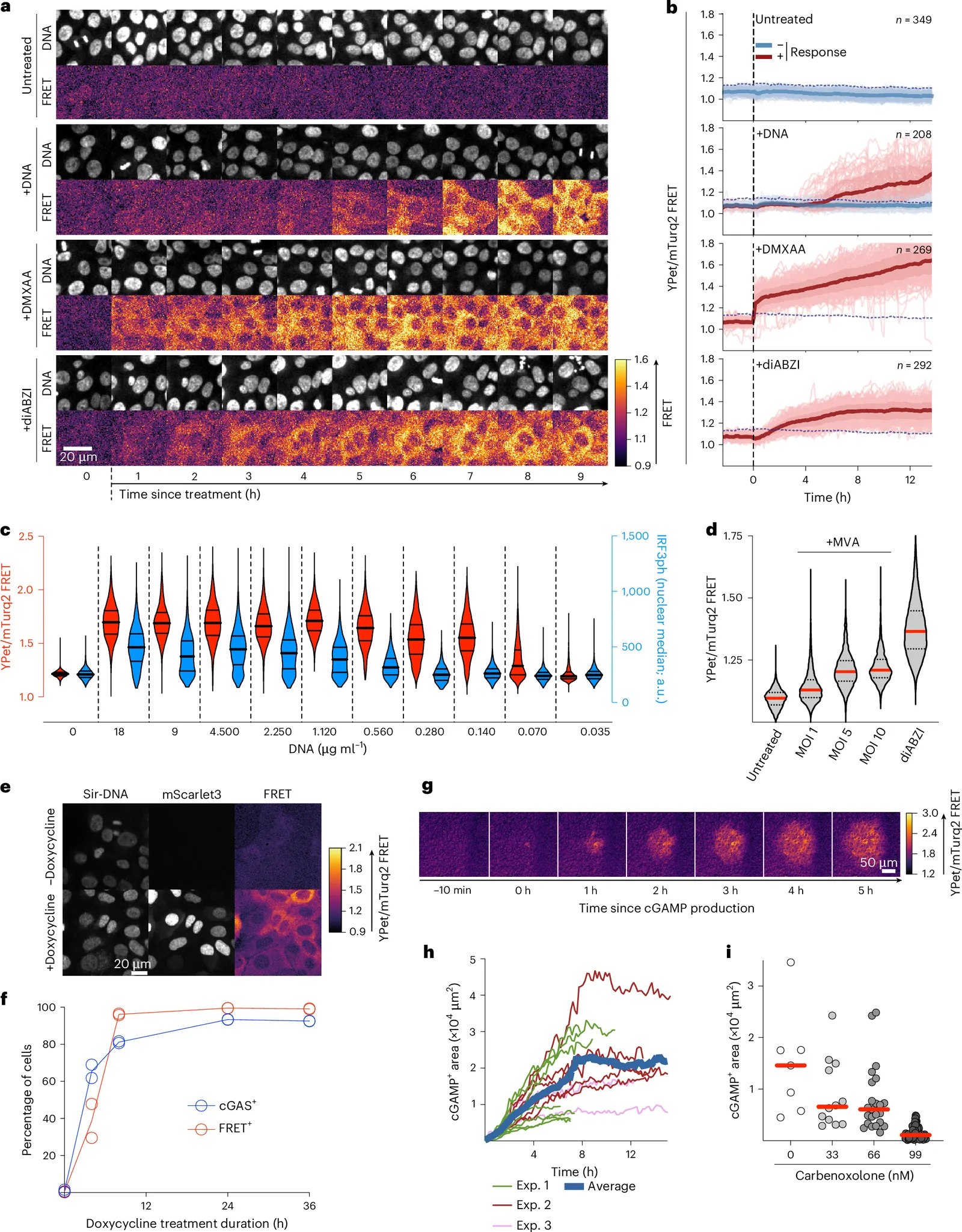
The improved cGAMP reporter sensitively detects cellular cGAS activation. **a**,**b**, Time-lapse microscopy of MCF10A cells stably expressing the cGAMP reporter following mock-treatment, DNA transfection, or treatment with either DMXAA (10 μg ml^−1^) or diABZI (1 μM). Representative images (**a**) and quantifications (**b**, dark lines, average; shaded area, s.d.) are provided. **b**, Note that not all cells received DNA during the DNA transfection, allowing us to divide cells into responders (transfected) and non-responders (untransfected) at the end of the imaging procedure. The dashed line marks the upper limit of the FRET signal over time in control cells from the same experiment. **c**, Comparison of the cGAMP-reporter FRET response (red, left *y* axis) with the nuclear signal of phospho-Ser396 of IRF3 (IRF3ph; blue, right *y* axis) in MCF10A cells stably expressing the cGAMP reporter. The cells were transfected with the indicated concentrations of DNA and FRET was analysed after 24 h. The cells were subsequently fixed and stained with the indicated antibodies. One of two independent repeats is shown (bold line, median; light lines, interquartile range); a.u., arbitrary units. **d**, Response of cGAMP reporter-expressing MCF10A cells following treatment with MVA at the indicated multiplicity of infection (MOI) or 1 μM diABZI compared with untreated cells. Images were taken 8 h after infection or diABZI treatment. **c**,**d**, One of two independent repeats is shown (bold line, median; light lines, interquartile range). **e**,**f**, Representative images (**e**) and quantification (**f**; averages from two independent experiments) of the cGAMP reporter response to inducible expression of mScarlet3–NLS–cGAS_R255E in cGAS-disrupted cells. **g**,**h**, Analysis of cGAMP spreading between cells. cGAMP reporter-expressing MCF10A cells were subjected to DNA transfection with limiting amounts of DNA, resulting in individual cGAS activation events. An individual example (**g**) and individual traces as well as averages from three experiments (**h**; exp. 1–3) are shown. Trace ends indicate tracking until the last time point of the time lapse. **i**, Analysis of cGAMP spreading in reporter-expressing MCF10A cells subjected to DNA transfection with limiting amounts of DNA and either mock-treated or treated with the indicated concentrations of the gap junction inhibitor carbenoxolone. The maximal expansions of individual spreading events (dots) after 18 h from one of two independent repeats are shown. Red line, median. Source numerical data are provided. Source data

**Extended Data Fig. 2:**
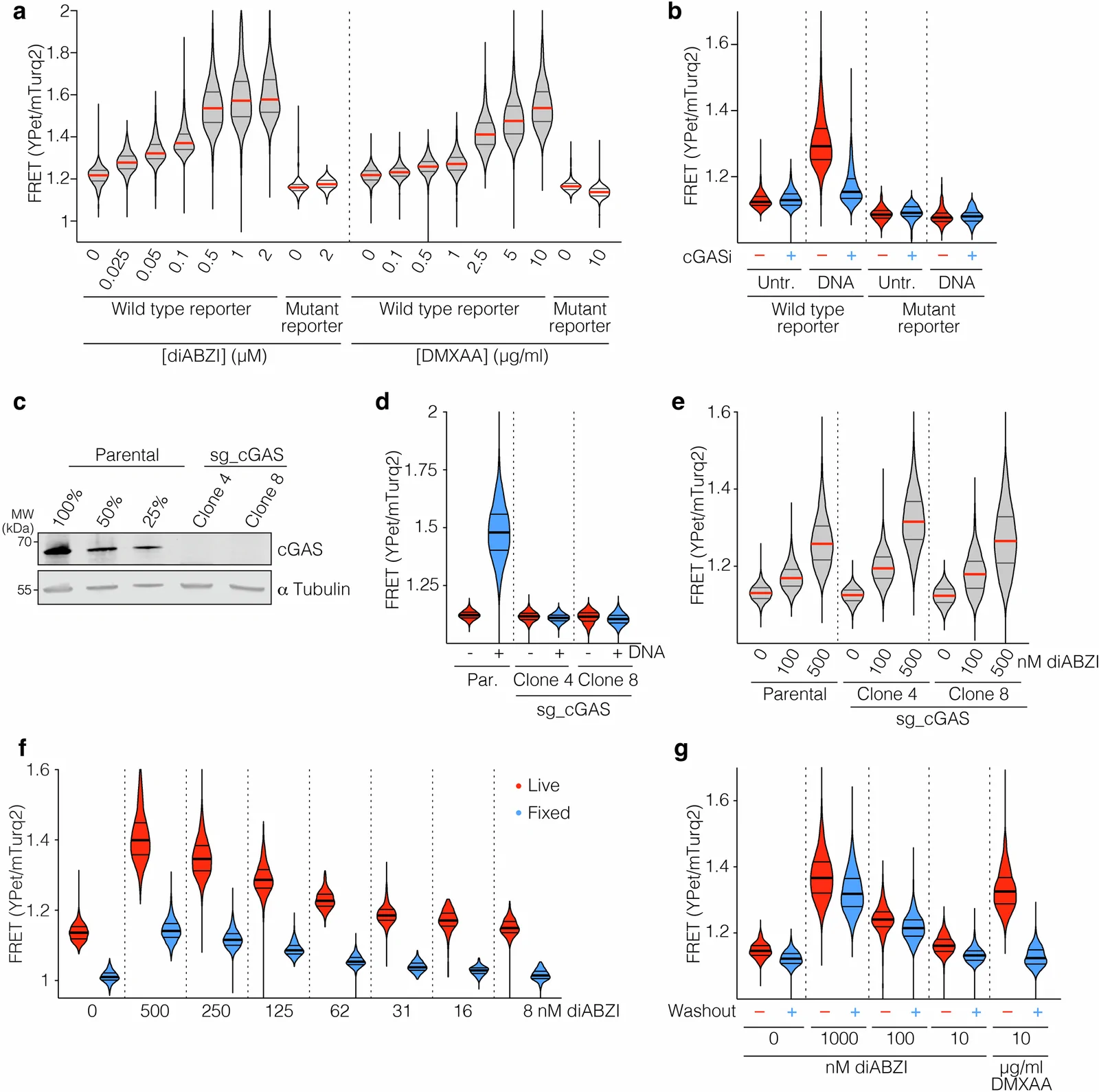
Microscopic characterization of the cGAMP reporter. **a**, FRET response of MCF10A cells expressing either wild-type or mutant reporter following 15 h incubation with the indicated concentrations of diABZI or DMXAA. Red horizontal lines, median; black horizontal lines, interquartile range (*n* = 300–1,400 cells for each condition). Shown is one of three independent experiments. **b**, FRET response of MCF10A cells expressing either wild-type or mutant reporter 8 h after mock-transfection or transfection with 1.33 µg ml^−1^ DNA, with or without 10 µM cGAS inhibitor (cGASi) G150. Bold horizontal lines, median; light horizontal lines, interquartile range (*n* = 43–600 cells for each condition). Shown is one of two independent experiments. **c**–**e**, Analysis of cGAMP-reporter FRET response in cGAS-knockout MCF10A cells. **c**, Western blot analysis of the indicated proteins from the indicated cell lines. To assess depletion levels, a dilution series of lysates from the parental cell line is shown. **d**, FRET response of the indicated cells expressing the cGAMP reporter 15 h after mock-transfection or transfection with 1.33 µg ml^−1^ DNA. Bold horizontal lines, median; light horizontal lines, interquartile range (*n* = 670–2,200 cells for each condition). Shown is one of two independent experiments. **e**, FRET response of the indicated cells expressing the cGAMP reporter 15 h after treatment with 1 µM diABZI. Bold horizontal lines, median; light horizontal lines, interquartile range (*n* = 570–4,000 cells for each condition). **f**, Comparison of FRET signal of cGAMP reporter-expressing MCF10A cells imaged under live conditions (red) or following fixation (blue). Cells were incubated for 15 h with the indicated concentrations of diABZI, imaged, fixed and imaged again. Bold horizontal lines, median; light horizontal lines, interquartile range (*n* = 127–1,600 cells for each condition). Shown is one of two independent experiments. **g**, Comparison of FRET signal of cGAMP reporter-expressing MCF10A cells incubated for 15 h with the indicated concentrations of diABZI or DMXAA, imaged either before (red) or after 60 min of washout (blue). Bold horizontal lines, median; light horizontal lines, interquartile range (*n* = 200–1,350 cells for each condition). Shown is one of three independent experiments. Source numerical data and unprocessed blots are provided.

To evaluate cGAS activation at the single-cell level, we stably inserted wild-type or mutant reporter into MCF10A non-cancerous breast epithelial cells, which were previously used to extensively characterize cGAS responses to genotoxic stress^21,43^. Constructs were delivered via lentiviruses and, to prevent recombination during reverse transcription, we reduced the homology between mTurq2 and YPet by codon-optimizing YPet for *E. coli*^44^. Confocal microscopy of live cells that were mock-treated, subjected to DNA transfection or treated with one of two STING agonists, DMXAA^45–47^ or diABZI, revealed rapid responses to DNA and both agonists with the wild-type reporter but not the mutant reporter (Fig. 2a,b, Extended Data Fig. 2a,b and Supplementary Video 1). Intracellular cGAS concentrations are estimated to be ≥100 nM (ref. ^48^), which suggests that cGAMP levels might also be in the nanomolar-to-micromolar range. Within this concentration range, FRET shifts are readily detected, indicating responsiveness to physiological levels of simulation (Extended Data Fig. 2a). As expected, signal induced by DNA transfection was sensitive to chemical inhibition^49^ of cGAS (Extended Data Fig. 2b) and was lost following CRISPR–Cas9-mediated cGAS disruption (Extended Data Fig. 2c,d), whereas cGAS loss did not affect the response to diABZI (Extended Data Fig. 2e). FRET was partially preserved after formaldehyde fixation (Extended Data Fig. 2f). There were also kinetic differences, with DMXAA promoting faster responses than diABZI (Fig. 2a,b). Conversely, washout did not change FRET for diABZI but returned DMXAA-induced FRET to baseline levels, presumably reflecting differential off-rates or membrane translocation rates of the two agonists (Extended Data Fig. 2g).

**Extended Data Fig. 3:**
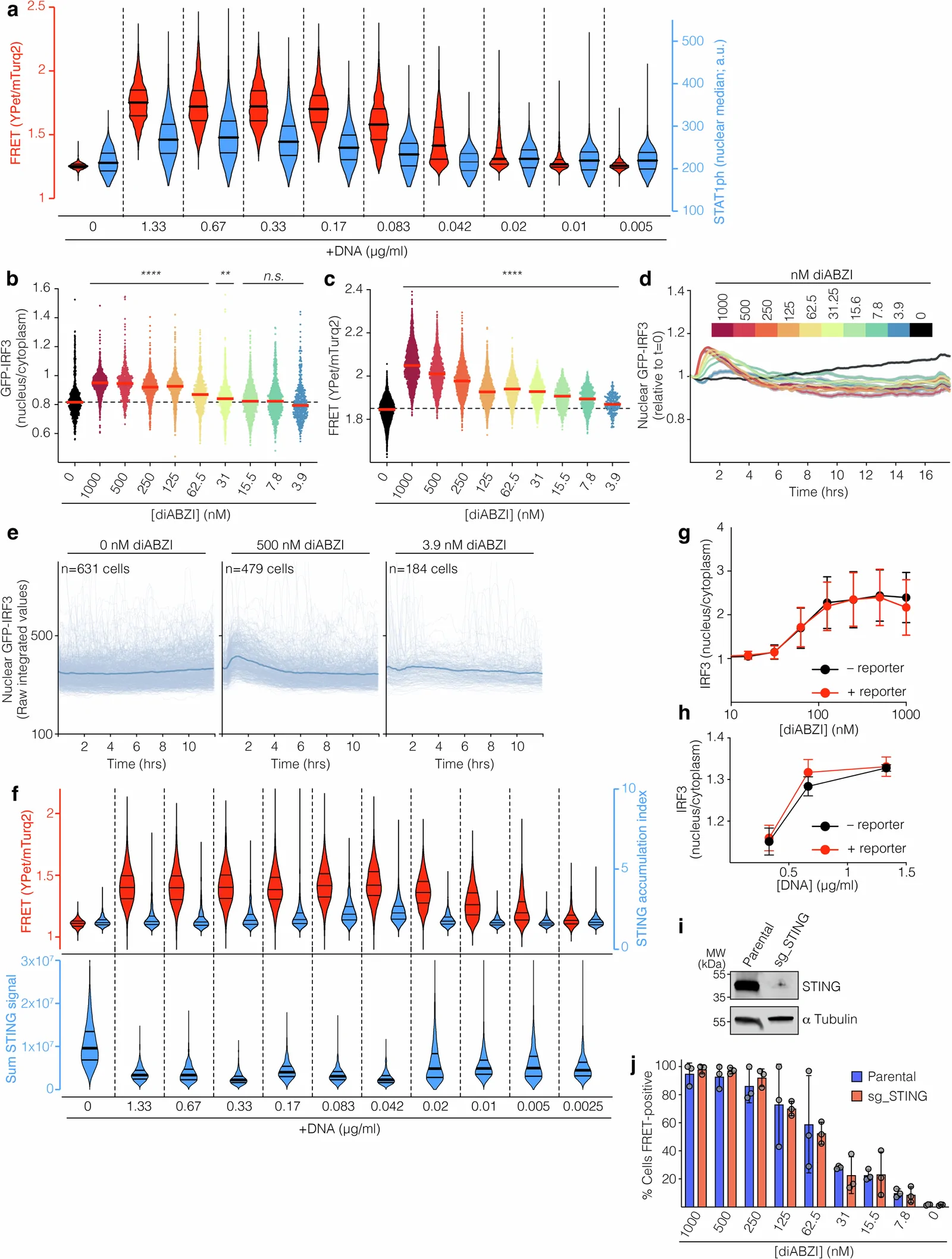
Comparison to other potential single-cell cGAS activation assays. **a**, Comparison of cGAMP-reporter FRET response (red, left *y* axis) with nuclear signal of phospho-Tyr701 of STAT1 (STAT1ph) (blue, right *y* axis). Cells were transfected with the indicated concentrations of DNA, and FRET was analysed after 16 h. Subsequently, cells were fixed and stained with the indicated antibodies. Bold horizontal lines, median; light horizontal lines, interquartile range. Shown is one of two independent experiments. **b**–**e**, MCF10A stably expressing GFP–IRF3 were treated with the indicated diABZI doses, and imaged every 5 min for 18 h. **b**, Column scatter plot of GFP–IRF3 nucleus/cytoplasm ratio at 60 min for every cell. Dots represent values from individual cells, red horizontal bar is the population median. **c**, In parallel to **b**, MCF10A cells stably expressing the cGAMP reporter were treated with the same diABZI doses. FRET in each cell was measured by imaging 18 h after stimulation with diABZI. For **b**,**c**, two-tailed *t*-test, ns: non significant, **p < 0.01, ****p < 0.0001. **d**, Mean ± s.e.m. of nuclear IRF3 normalized to t0. **e**, Raw signal traces of individual cells (light blue) and population means (dark blue) for the indicated concentrations of diABZI. **f**, Comparison of cGAMP-reporter FRET response (red, left *y* axis) with STING accumulation index (maximum signal divided by the average signal; blue, right *y* axis on top graph) and total STING signal intensity per cell (bottom graph). Cells were transfected with the indicated concentrations of DNA, and FRET was analysed after 8 h. Subsequently, cells were fixed and stained with the indicated antibodies. Bold horizontal lines, median; light horizontal lines, interquartile range. Shown is one of two independent experiments. **g**, Comparison of downstream signalling between MCF10A cells expressing the cGAMP reporter, and the parental cell line, as assessed by analysis of nuclear translocation of IRF3. Cells were incubated for 90 min with the indicated concentrations of diABZI, following which they were fixed and stained for total IRF3. Means and standard deviations of ~3,000 cells per condition are shown from one representative experiment out of two independent repeats. **h**, Comparison of downstream signalling between MCF10A cells expressing the cGAMP reporter, and the parental cell line, as assessed by analysis of nuclear translocation of IRF3. Cells were transfected with the indicated concentrations of DNA (pBluescript), and fixed after 6 h. Cells were then stained for total IRF3. Note that for this analysis, due to the heterogeneous effect of DNA transfection (Fig. 2a,b), we focused on responder cells. Means and standard deviations from three independent experiments are shown. **i**,**j**, Comparison of cGAMP reporter-expressing MCF10A cells with a STING-disrupted derivative. **i**, Western blot analysis of STING-knockout efficiency. **j**, Live FRET imaging of the indicated cells treated with the indicated amounts of diABZI for 16 h. Values and averages from three experiments are shown. Error bars, standard deviation. Source numerical data and unprocessed blots are provided.

To compare reporter sensitivity with established methods detecting downstream events, we transfected cells with varying amounts of DNA or treated them with varying concentrations of diABZI, carried out live FRET imaging, and subsequently fixed and stained the cells with antibodies to either IRF3 phospho-Ser396 (IRF3ph) or STAT1 phospho-Tyr701 (STAT1ph). Our reporter exhibited superior sensitivity compared with the alternative assays (Fig. 2c and Extended Data Fig. 3a). Furthermore, it exhibited higher sensitivity compared with analysis of the nuclear translocation of GFP–IRF3 (Extended Data Fig. 3b,c). Moreover, GFP–IRF3 nuclear translocation was transient and detectable only within a narrow time window (Extended Data Fig. 3d,e), and signal in response to lower stimuli was probably only apparent due to the synchroneity of the response and prior normalization to the starting time point (Extended Data Fig. 3d,e). It is therefore unlikely that small fractions of cells weakly activating GFP–IRF3 asynchronously could be robustly detected. Similar reasons are also likely to reduce sensitivity of IRF3ph- and STAT1ph-based readouts. Finally, we compared our reporter to analysis of STING relocalization, which is also used to infer cGAS activation state^33^. This again revealed reduced sensitivity compared with our reporter (Extended Data Fig. 3f(top)). Furthermore, due to the activation-induced degradation of STING, detection was limited to a Goldilocks-zone of intermediate stimulus (Extended Data Fig. 3f(bottom)).

One possible limitation of our reporter is that it might compete for cGAMP with endogenous STING, thereby interfering with downstream signalling; reciprocally, endogenous STING might limit reporter sensitivity. To test the former, we treated MCF10A cells stably expressing the reporter as well as the parental cells lacking the reporter with a range of diABZI concentrations. IRF3 nuclear translocation was subsequently used as a proxy for downstream cGAS signalling. No difference was observed (Extended Data Fig. 3g), indicating that in this cell line, the reporter does not interfere with STING-dependent signalling. Similar results were observed with DNA transfection instead of diABZI treatment (Extended Data Fig. 3h). To test whether endogenous STING limits reporter sensitivity, we disrupted STING in the reporter MCF10A line using CRISPR–Cas9 (Extended Data Fig. 3i). STING loss did not alter the response to diABZI (Extended Data Fig. 3j), showing that in this cell line, no obvious competition exists between endogenous STING and the reporter.

We next assessed the response to cGAS activation by virus infection, a more physiological context than plasmid transfection or STING-agonist treatment. We previously demonstrated that only limited cGAS-dependent IFN production is detected following infection with modified vaccinia virus Ankara (MVA)^50^. This is due to the viral E5R gene, which encodes a cGAS inhibitor. Infection with MVA is therefore an attractive means to test the true sensitivity of our reporter. Reflecting the high sensitivity of the reporter, MVA infection promoted dose-dependent FRET increases (Fig. 2d). Consistent with an attenuated response due to E5R, the signal remained below that induced by saturating diABZI concentrations (Fig. 2d). Whereas vaccinia-like viruses replicate in the cytoplasm, other DNA viruses replicate in the nucleus, raising the potential for nuclear cGAS activation. As our reporter is predominantly cytoplasmic (Fig. 2a) and we therefore generally analysed cytoplasmic signal (Methods), we next determined whether it could nonetheless detect cGAMP generated in the nucleus. We therefore rescued cGAS-disrupted cells with an inducible nuclear localization sequence (NLS)-tagged cGAS carrying the R255E mutation, which prevents inhibition by nucleosomes^10^ and promotes cGAMP generation from chromatin^7,10^. This revealed widespread reporter activation that correlated with cGAS induction kinetics (Fig. 2e,f), consistent with the idea that cGAMP can freely diffuse through nuclear pores^38^. We conclude that our cGAMP reporter is the most sensitive tool for detection of cGAS activation currently available, it responds to a variety of cGAS stimulants and STING agonists, and it does not interfere with downstream signalling when expressed at suitable concentrations.

### cGAMP is transmitted through gap junctions over multiple layers of cells

Following DNA transfection, cGAS activation frequently occurred in clusters of cells (Fig. 2a,b and Supplementary Video 1), suggesting intercellular cGAMP transmission, as reported previously^51^. To investigate this, we transfected DNA with limiting amounts of transfection reagent, resulting in individual activation-transmission events that transmitted over distances equivalent to multiple layers of cells (Fig. 2g,h and Supplementary Video 2). The transmission speed eventually stagnated, potentially due to dilution of cGAMP with each cell layer. Analysis of individual traces revealed a large degree of variability (Fig. 2h). As indicated by previous literature for other cell types^51^, inhibition of gap junctions with carbenoxolone largely abrogated cGAMP spreading (Fig. 2i), indicating that differences in gap-junction formation may at least partially underlie the differences in cGAMP transmission. These data show that the reporter allows the visualization of cell non-autonomous sources of cGAMP in cells.

### DNA damage and chromosomal instability promote weak cGAS activation in only a subpopulation of cells

**Fig. 3:**
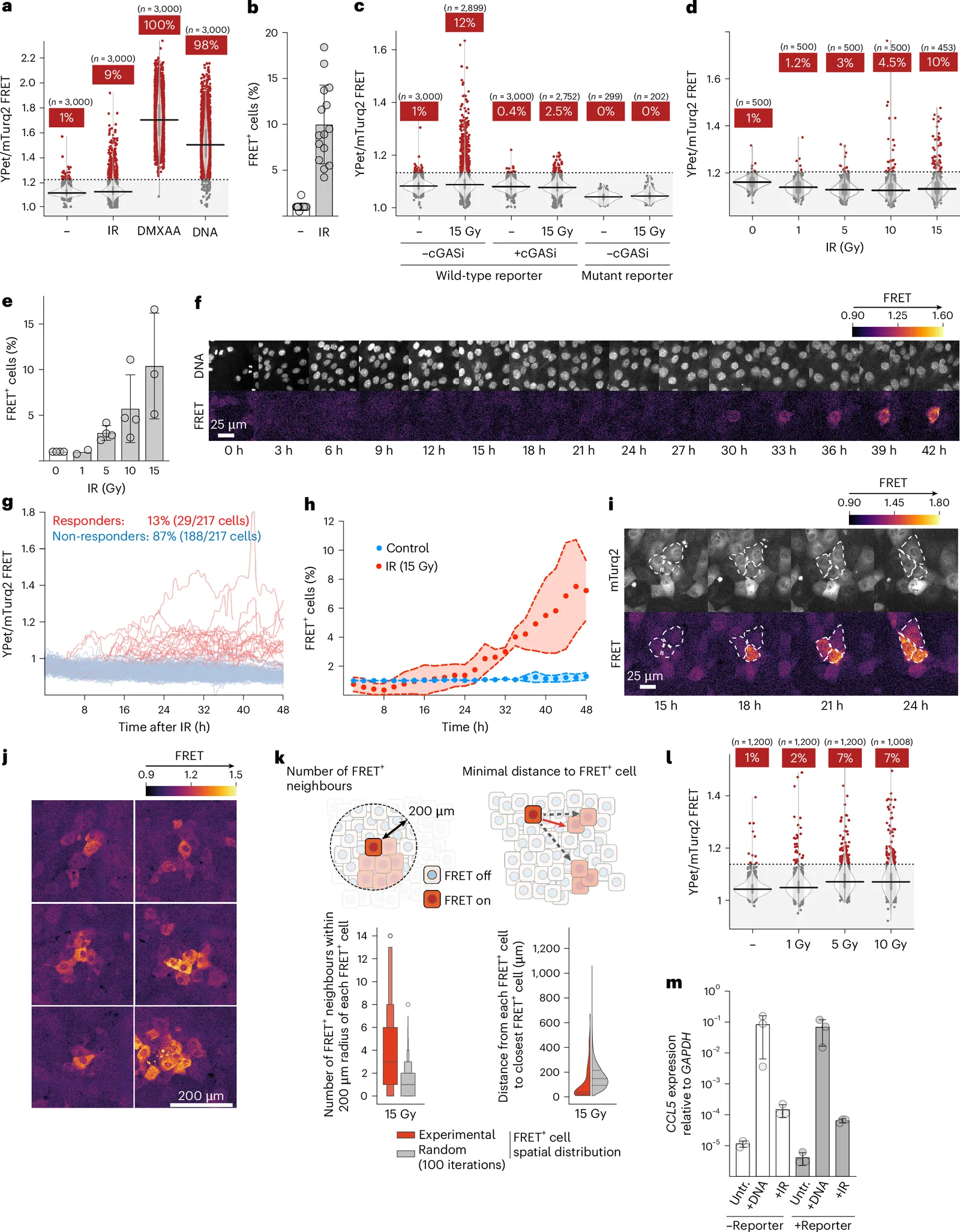
Genotoxic stress promotes only a weak cGAS response that manifests in a minority of cells. **a**–**l**, Analysis of cGAMP-reporter FRET response following the indicated treatments. **a**,**b**, Comparison of cGAMP-reporter FRET responses in untreated cells, cells subjected to IR (15 Gy; analysis after 48 h) or DNA transfection (1.33 μg ml^−1^; analysis after 8 h) and cells treated with DMXAA (10 μg ml^−1^; 2 h treatment). Results from an individual experiment (**a**) and average ± s.d. of 15 individual IR (15 Gy) experiments (**b**). **c**, Analysis of the FRET response of cells expressing the indicated constructs with or without treatment with the cGAS inhibitor G150 (10 μM; cGASi) and IR. Analysis was performed 48 h after irradiation. One of three independent experiments is shown. **d**,**e**, Analysis of the FRET response of cells expressing the cGAMP reporter 48 h after irradiation with the indicated doses of IR. Representative experiment (**d**), and average ± s.d. of two (1 Gy), three (15 Gy) and four (0, 5 and 10 Gy) independent experiments (**e**). **f**–**i**, Time-lapse analysis of the FRET response of the cGAMP reporter to 7 Gy (**f**) or 15 Gy (**g**–**i**). **f**, Images indicating cGAS activation in an individual cell. **g**, FRET traces of individual cells (*n* = 217). One of three independent experiments is shown. **h**, Fraction of cells with a FRET^+^ response at different time points following irradiation. Dots indicate the average and the shaded area (bounded by dotted lines) indicates the s.d. of two independent experiments. **i**, Example of cGAS activation in a single cell followed by reporter response in neighbouring cells. Dotted lines outline relevant cells. **j**, Examples of FRET^+^ cell clusters 48 h after irradiation with 15 Gy. **k**, Analysis of the spatial distribution of FRET^+^ cells. Schematic explanation of the two analyses (top). Results from the experimental data compared with 100 iterations of a random distribution. Data on the left are represented as letter value boxenplots and data on the right as split violin plots. One of two independent repeats is shown. **l**, FRET response six days after irradiation with the indicated doses of IR. One of two independent repeats is shown. **m**, Quantitative reverse-transcription PCR analysis of CCL5 induction by DNA (6 h post transfection) and IR (48 h after 15 Gy irradiation). Data are the average ± s.d. of three individual experiments; untr., untreated. **a**,**c**,**d**,**l**, The dots represent the FRET values of individual MCF10A cells expressing the indicated versions of the cGAMP reporter. The violin plots show the general distribution, with the median indicated by a horizontal line and the interquartile range indicated by a grey vertical line. Red dots indicate cells containing cGAMP (as defined by a FRET response higher than 99% of control cells). The numbers in the red boxes indicate the percentage of cells containing cGAMP. Source numerical data are provided. Source data

**Extended Data Fig. 4:**
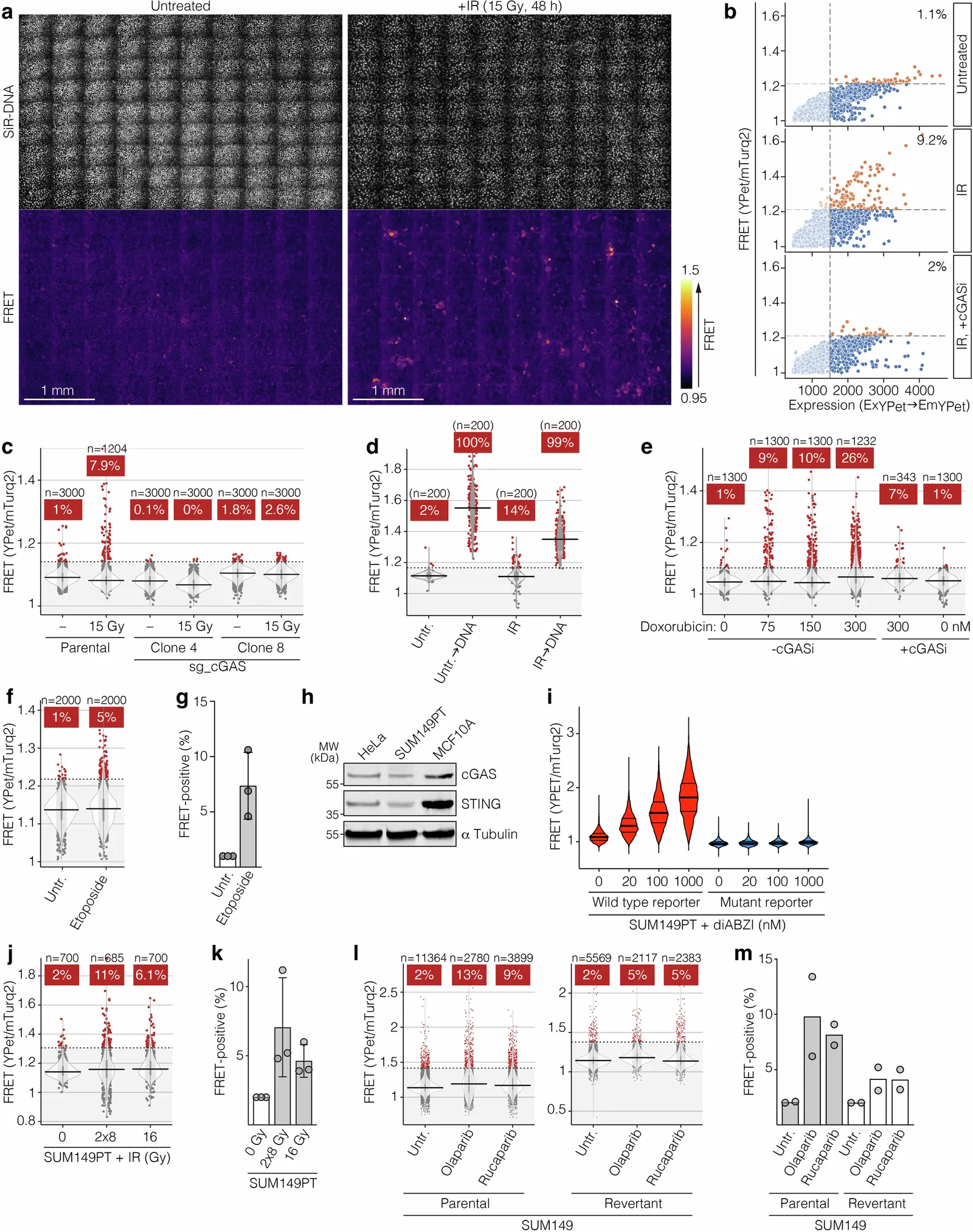
Multiple sources of DNA damage lead to only rare cGAS activation. MCF10A cells stably expressing the cGAMP reporter, or (where indicated) SUM149PT cells stably expressing the reporter were either untreated or subjected to the indicated treatments. For (**c**–**f**,**j**,**l**), dots represent values from individual cells expressing the cGAMP reporter. Violin plots show the general distribution, with the median indicated by a horizontal line, and the interquartile range indicated by a grey vertical line. Red dots indicate cells containing cGAMP (as defined by a FRET response higher than 99% of control cells). Numbers in red boxes indicate the percentage of cells containing cGAMP. **a**, Overview of a large region of untreated or irradiated cells imaged for DNA content (upper panels) or cGAMP-reporter FRET (lower panels), generated by computational stitching of multiple imaging views (see Methods for details). Please note the slight stitching artefact that causes darker edges around each field of view. **b**, Scatter plots indicating the relationship between cGAMP reporter expression levels (*x* axis) and FRET (*y* axis). Cells were either untreated, or treated with 15 Gy of IR in the presence or absence of 10 µM cGAS inhibitor G150. After 48 h, cells were analysed by live-microscopy. Cells of a YPet intensity below 1,500 were considered too low-expressing for reliable cGAMP detection. (See Methods section ‘Image processing and data analysis’.) **c**, Analysis of cGAMP-reporter FRET in parental cells as well as the cGAS-knockout clones from Extended Data Fig. 2c. Cells were untreated or irradiated and imaged 48 h later. Shown is one of two independent experiments. **d**, MCF10A cells stably expressing the cGAMP reporter were either untreated, or treated with 15 Gy of IR. After 40 h, each of the samples was either transfected with 1.33 µg ml^−1^ DNA or left untransfected. Cells were imaged and for FRET ratio measurement 8 h later. Shown is one of two independent experiments. **e**, cGAMP-reporter FRET response following doxorubicin. Cells were incubated with the indicated concentrations of doxorubicin, either on its own, or in the presence of 10 µM cGAS inhibitor G150. FRET imaging was performed after 48 h. Due to fluorescence from doxorubicin affecting the baseline of measured FRET, measured FRET values from each cells were normalized to the average FRET value of MCF10A stably expressing the mutant cGAMP-blind reporter that were treated with the same dose of doxorubicin and imaged in parallel. Shown is one of three independent experiments. **f**,**g**, cGAMP-reporter FRET response to etoposide. Cells were treated with 10 µM etoposide for 48 h before analysis. Shown is a representative image (**f**) and averages with standard deviations from three independent experiments (**g**). **h**, Western blot comparing cGAS and STING expression levels between HeLa, SUM149PT and MCF10A cell lines (*n* = 1 independent experiment). **i**, FRET response of SUM149PT cells stably expressing either wild-type or mutant reporter following 16 h incubation with the indicated concentrations of diABZI. Shown is one out of two independent experiments. **j**,**k**, SUM149PT cells stably expressing the cGAMP reporter were either untreated, treated with 16 Gy of IR on day 1, or treated with 8 Gy of IR on days 1 and 2. Cells were imaged on Day 3. Shown is one representative experiment (**j**) and averages with standard deviation from three independent experiments (**k**). **l**,**m**, SUM149PT or BRCA1-revertant cells were either mock-treated or treated with the indicated concentrations of PARPi for 4 days. Shown is one experiment (**l**) and averages from two independent experiments (**m**). Source numerical data and unprocessed blots are provided.

We next investigated the cGAS response to DNA damage, starting with 15 Gy ionizing radiation (IR), similar to previous studies^21^. Surprisingly, only a minor fraction (approximately 10%) of cells showed evidence of cGAMP production and the signal in each activating cell was generally lower than following agonist treatment or DNA transfection (Fig. 3a,b and Extended Data Fig. 4a). FRET occurred over a range of reporter expression levels, indicating that there was no specific expression level requirement (Extended Data Fig. 4b). As expected, cGAS inhibition largely abolished this response and no FRET change was observed with the mutant reporter, confirming that the response to IR depends on cGAS and cGAMP (Fig. 3c and Extended Data Fig. 4b). Similarly, CRISPR–Cas9-mediated cGAS disruption abolished reporter response (Extended Data Fig. 4c). DNA transfection at the end of the experiment confirmed that all cells had maintained competence for cGAS activation and reporter stimulation (Extended Data Fig. 4d). Lower IR doses resulted in an even weaker response (Fig. 3d,e). Similar limited responder fractions were observed with doxorubicin and etoposide, other inducers of DNA damage (Extended Data Fig. 4e–g), and in the breast cancer-derived SUM149PT cell line treated with IR (Extended Data Fig. 4h–k). SUM149PT cells carry a mutation in *BRCA1* that sensitizes them to PARP-inhibitor treatment and also drives cGAS-dependent inflammatory responses^52^. Consistent with this, treatment with two PARP inhibitors, olaparib and rucaparib, promoted cGAS activation but, once again, only in a minority of cells (Extended Data Fig. 4l,m). Cells in which *BRCA1* was restored to wild type^53^ showed an even lower response (Extended Data Fig. 4l,m). We conclude that multiple sources of DNA damage lead to only limited cGAS activation.

**Extended Data Fig. 5:**
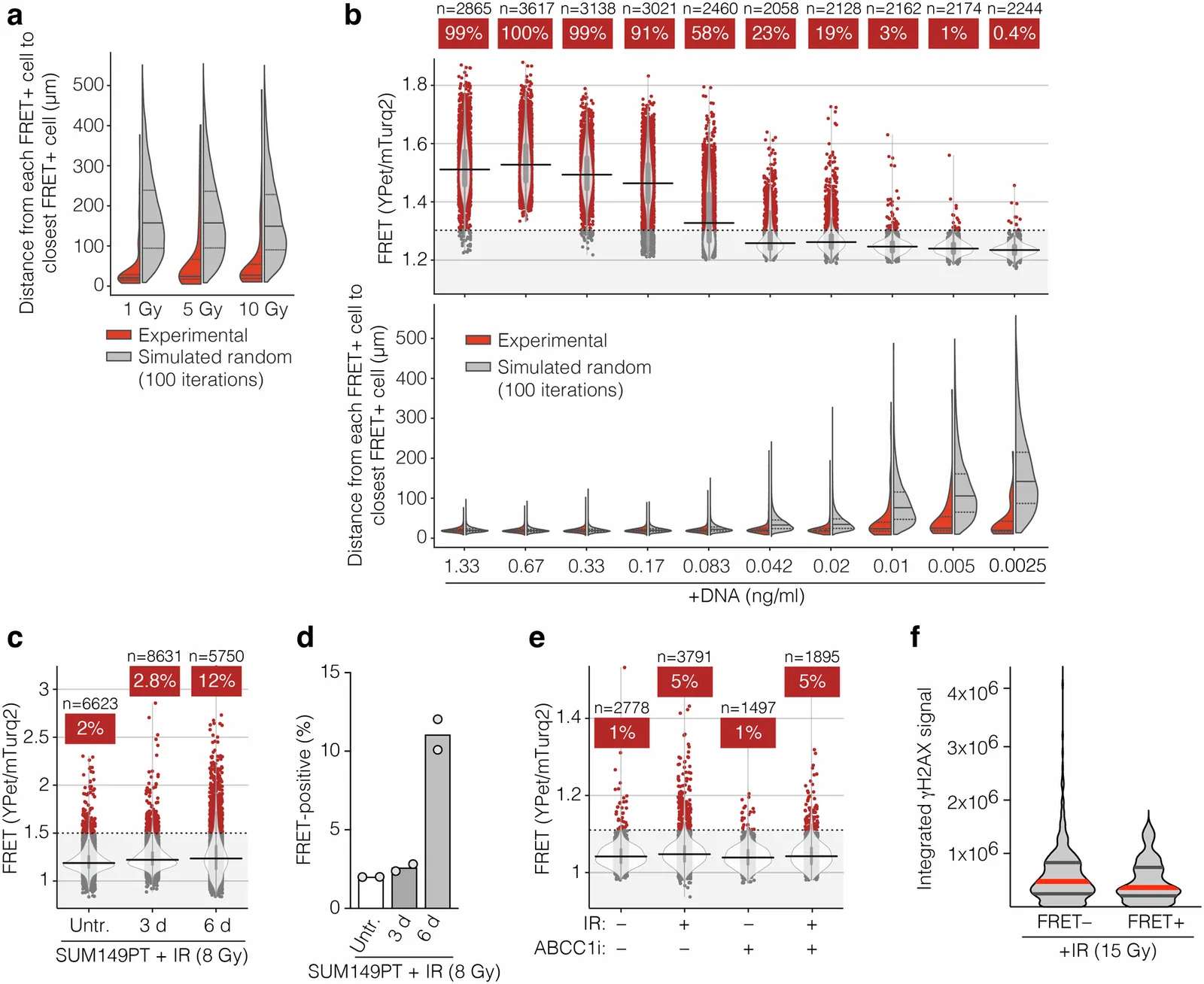
Further characterization of the cGAS response to DNA damage. **a**, Analysis of minimal distance between FRET^+^ cells as per schematic from Fig. 3k, 48 h after treatment of MCF10A cells stably expressing the cGAMP reporter with the indicated doses of IR. Shown is one of two independent experiments. **b**, MCF10A cells stably expressing the cGAMP reporter were transfected with the indicated concentrations of pBlueScript DNA. After 8 h, FRET imaging was performed (top), and minimal distance between FRET^+^ cells (bottom) was performed as per schematic from Fig. 3k. Shown is one of two independent experiments. **c**,**d**, Comparison of SUM149PT cells stably expressing the cGAMP reporter irradiated, and imaged for reporter FRET either after three days, or after six days. Shown is one of two independent experiments (**c**), and averages of two experiments (**d**). **e**, Analysis of the effect of ABCC1 inhibition on the cGAS response to IR. MCF10A cells stably expressing the reporter were irradiated with 15 Gy, incubated in the presence of 25 µM of the ABCC1 inhibitor MK-571 for 48 h, and subsequently imaged for reporter FRET. Shown is one of three independent experiments. **f**, Correlative analysis of cGAMP-reporter FRET and γH2AX immunofluorescence. Cells were imaged for FRET under live conditions 48 h after treatment with 15 Gy of IR. Subsequently, cells were fixed and stained. Corresponding cells were identified in each dataset. Red line, median; black lines, interquartile range. Source numerical data are provided.

Time-lapse microscopy following irradiation furthermore revealed large temporal heterogeneity in the cGAS response (Fig. 3f–h and Supplementary Video 3). This also revealed that cGAS activation in one cell was frequently followed by the appearance of FRET signals in neighbouring cells (Fig. 3i,j), consistent with cGAMP transmission. To further characterize this potentially clustered activation, we employed two metrics (Fig. 3k(top)). First, we quantified the number of activated neighbours surrounding each active cell within a radius of 200 μm. Second, we measured the distance from each activated cell to its nearest active neighbour. The results were compared with 100 iterations of randomly assigning activation status to cells based on the proportion of activated cells. Both metrics indicated that clustered activation occurred more frequently than random (Fig. 3k(bottom)). Lower radiation doses did not change this behaviour (Extended Data Fig. 5a). Transfection with a dilution series of DNA verified this approach by showing differences between experimental and random populations only in conditions of low activation from limiting DNA transfection (Extended Data Fig. 5b), where we had previously observed cGAMP transfer. Because cells did not tolerate carbenoxolone over the multiday time frame of the irradiation experiment, we could not establish whether activation events clustered after IR due to cGAMP transfer via gap junctions.

It was previously suggested that IR-induced cGAS activation takes multiple (3–6) days^21,54^, as assayed by the appearance of STAT1ph. Therefore, we also analysed cGAS activation after six days. Although there were increases in cGAMP^+^ cells after six days, particularly for SUM149PT cells, this remained the range of approximately 10% of cells (compare Fig. 3l with Fig. 3d; Extended Data Fig. 5c,d). This suggests that the increase in STAT1ph over time reflects both a modest rise in cGAS activation and amplification of downstream signalling, perhaps through an increased number of cells responding to IFN-β secreted by the small responder fraction.

As cGAMP can be exported by ABCC1 (ref. ^55^), export could account for the low responder fraction. However, ABCC1 inhibition with a high dose of MK-571 (ref. ^55^) did not alter the responder frequency in irradiated cells (Extended Data Fig. 5e).

To support our single-cell analysis with a population-based assay, we analysed induction of the ISG CCL5. Although IR resulted in a cGAS-dependent tenfold increase in CCL5 expression, it was nonetheless orders of magnitude less than the induction achieved by DNA transfection (Fig. 3m). We conclude that DNA damage provides only a weak stimulant for cGAS activation that manifests itself in only a minority of cells.

### Micronuclear cGAS accumulation generally does not lead to cGAS activation

**Fig. 4:**
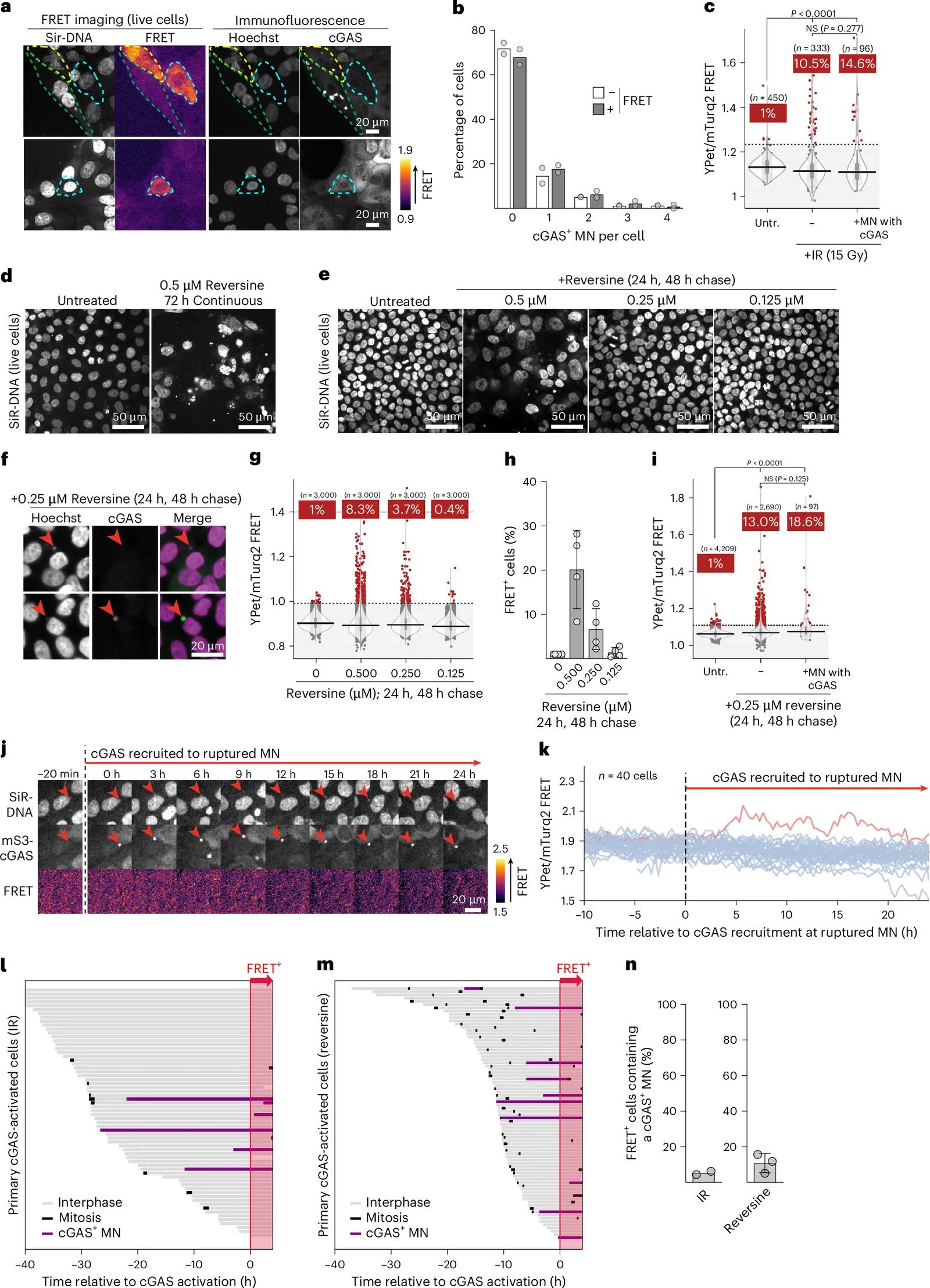
Radiation- and chromosome mis-segregation-induced cGAS activation is independent of its micronuclear enrichment. MCF10A cells stably expressing the reporter were either untreated or subjected to the indicated treatments. **a**–**c**, Analysis of correlative live-microscopy cGAMP-reporter FRET imaging, followed by immunofluorescence-based analysis of cGAS enrichment on micronuclei. Cells were analysed 48 h after irradiation. **a**, Representative examples of cells with micronuclear cGAS (top) that do (outlined in yellow) and do not (outlined in green) show reporter activation as well as cells with an active reporter that do not show evidence of micronuclear cGAS (top and bottom, outlined in cyan). **b**,**c**, Quantitative analysis of the correlation between cGAS-enriched micronuclei and cGAMP-reporter FRET in cells treated with 15 Gy irradiation. **b**, Averages of two independent experiments (dots). **c**, Cells exposed to 15 Gy radiation were categorized depending on the absence (–) or presence (+) of a cGAS^+^ micronucleus and according to the percentage of FRET^+^ cells. **d**,**e**, Representative images from one of >3 independent experiments of cells exposed to the indicated treatments with reversine. Cellular DNA was stained with SiR-DNA and subjected to live imaging. **f**–**i**, Cells were treated with the indicated dose of reversine (or dimethylsulfoxide control) for 24 h following media change and FRET imaging or immunofluorescence 48 h later. **f**, Representative cGAS immunofluorescence images showing micronuclei with (bottom) or without (top) cGAS accumulation. **g**,**h**, cGAMP response to reversine treatment. Representative experiment (**g**) and averages of four experiments (**h**; error bars, s.d.). **i**, Analysis of correlative live-microscopy cGAMP-reporter FRET imaging, followed by immunofluorescence-based analysis of cGAS enrichment on micronuclei. Cells were categorized depending on the absence or presence of a cGAS^+^ micronucleus and depending on the percentage of FRET^+^ cells compared with the untreated control. **c**,**i**, *P* values were derived using a two-tailed Fisher’s exact test; NS, not significant. One of two independent experiments is shown. **j**,**k**, Correlative time-lapse imaging of mScarlet3–cGAS enrichment on micronuclei and cGAMP-reporter FRET of cells treated with 0.5 μM reversine. Behaviour of a representative cell (**j**) and quantification (**k**) from one of three independent experiments. **k**, Cells were synchronized computationally to the time of cGAS recruitment to micronuclei. Each trace represents FRET values over time for individual cells. Cells that activate cGAS are displayed in red, inactive cells in blue. **l**,**m**, Lineage analysis of cells irradiated with 15 Gy (**l**) or treated with 0.5 μM reversine (**m**). Shown is the behaviour of each cell activating cGAS in a representative experiment for each condition (**l**, *n* = 64 cells, data from one of two independent experiments; **m**, *n* = 76 cells, data from one of three independent experiments. Note that these cells are the same as the ones analysed in Fig. 5g–i). Isolated responder cells or first responder cells in clusters were identified and back-traced throughout the time lapse. **n**, Percentage of responder cells in **l**,**m** that had a cGAS^+^ micronucleus. Averages of the two (IR, left) and three (reversine, right) experiments. **c**,**g**,**i**, The dots represent individual MCF10A cells expressing the cGAMP reporter. The violin plots show the general distribution, with the median indicated by a horizontal line and the interquartile range indicated by a grey vertical line. Red dots indicate cells containing cGAMP (as defined by a FRET response higher than 99% of control cells). The numbers in the red boxes indicate the percentage of cells containing cGAMP. MN, micronucleus; untr., untreated. Arrowheads in **f** and **j** indicate MN. Source numerical data are provided. Source data

**Extended Data Fig. 6:**
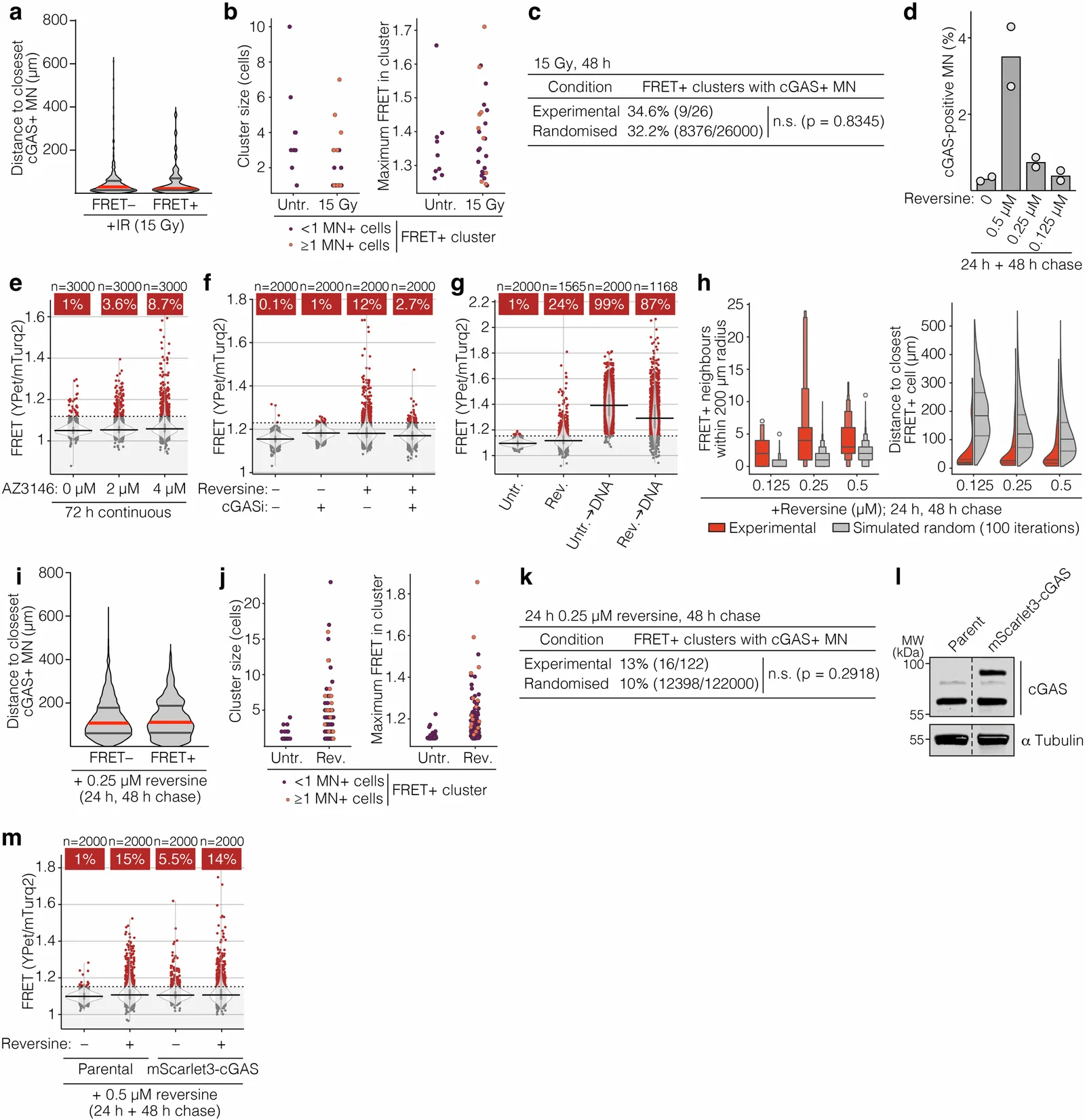
Analysis of DNA damage and cGAS localization parameters following ionizing radiation and chromosome mis-segregation. MCF10A cells stably expressing the cGAMP reporter were either untreated or subjected to the indicated treatments. For (**e**–**g**,**m**), dots represent values from individual cells expressing the cGAMP reporter. Violin plots show the general distribution, with the median indicated by a horizontal line, and the interquartile range indicated by a grey vertical line. Red dots indicate cells containing cGAMP (as defined by a FRET response higher than 99% of control cells). Numbers in red boxes indicate the percentage of cells containing cGAMP. MN, micronucleus. **a**, Correlative analysis of cGAMP-reporter FRET and cGAS immunofluorescence, used to determine the distance to the closest cGAS-enriched micronucleus in cells with and without FRET. Cells were imaged for FRET under live conditions 48 h after treatment with 15 Gy of IR. Subsequently, cells were fixed and stained. Corresponding cells were identified in each dataset. Red line, median; black lines, interquartile range. Shown is one of two independent experiments. **b**,**c**, Analysis of distribution of cGAS-positive micronucleated cells (MN^+^) with regards to clusters of FRET^+^ cells. Cells were left either untreated (Untr.) or irradiated with 15 Gy and analysed after 48 h. **b**, Comparison of cluster size (left) and maximum FRET value within a given cluster (right) for clusters without cGAS-positive micronuclei and clusters with cGAS-positive micronuclei. **c**, Comparison of the percentage of FRET^+^ clusters containing a cGAS-positive micronucleus with 100 iterations of randomized populations. *P* value derived by two-tailed Fisher’s exact test. Shown is one of two independent experiments. **d**, Percentage of MCF10A cells stably expressing the cGAMP reporter containing a cGAS-positive micronucleus following the indicated treatments. Shown are averages from two independent experiments. **e**, MCF10A stably expressing the cGAMP reporter were treated for 72 h with the indicated AZ3146 doses, and subsequently imaged for FRET ratio measurement. Shown is one of two independent experiments. **f**, MCF10A cells stably expressing the cGAMP reporter were grown for 72 h in the presence or absence of 0.5 µM reversine with or without co-treatment with 10 µM cGAS inhibitor G150, and subsequently imaged. Shown is one of two independent experiments. **g**, MCF10A cells stably expressing the cGAMP reporter were either untreated, or grown for 72 h in the presence or absence of 0.5 µM reversine. After 64 h, each of the samples was either transfected with 1.33 µg ml^−1^ DNA or left untransfected. Cells were imaged for FRET ratio measurement 8 h later. Shown is one of two independent experiments. **h**, Analysis of the spatial distribution of FRET^+^ cells after reversine treatment. FRET^+^ cells were analysed for number of FRET^+^ neighbours (left) and shortest distance to nearest other FRET^+^ cell (right) as per schematic in Fig. 3k. Shown is one of two independent experiments. **i**, Distance to closest cGAS-enriched micronucleus in cells with and without FRET after the indicated treatment. Cells were FRET imaged, followed by fixation and staining for cGAS. Red line, median; black lines, interquartile range. Shown is one of two independent experiments. **j**,**k**, Analysis of distribution of cGAS-positive micronucleated cells (MN^+^) with regards to clusters of FRET^+^ cells. Cells were left either untreated (Untr.) or treated with 0.25 µM reversine (Rev.) for 24 h followed by 48 h chase. **j**, Comparison of cluster size (left) and maximum FRET value within a given cluster (right) for clusters without cGAS-positive micronuclei and clusters with cGAS-positive micronuclei. **k**, Comparison of the percentage of FRET^+^ clusters containing a cGAS-positive micronucleus with 100 iterations of randomized populations. *P* value derived by two-tailed Fisher’s exact test. Shown is one of two independent experiments. **l**,**m**, Analysis of MCF10A cells stably expressing the cGAMP reporter and mScarlet3–cGAS. **l**, Western blot analysis of the indicated cell lines. The dotted vertical lines indicate removed irrelevant lanes. One independent experiment. **m**, cGAMP-reporter FRET analysis of the indicated cell lines following the indicated treatments. Source numerical data and unprocessed blots are provided.

To investigate differences between responders and non-responders, we FRET-imaged cells under live conditions, and subsequently fixed the cells and analysed them by immunofluorescence. Corresponding cells were then identified in both datasets (Methods). Using this procedure with γH2AX staining as a proxy for DNA damage, we established that cGAMP production was not generally correlated with the amount of persistent DNA damage that cells maintained (Extended Data Fig. 5f). However, rather than DNA damage itself, it is widely believed that enrichment of cGAS within micronuclei, derived from damaged chromosomes, drives cGAS activation^5,11,20–23^, despite recent studies challenging this paradigm^24,33–35^. To investigate this, we performed live-cell FRET imaging after IR, followed by immunofluorescence microscopy to visualize cGAS. Although cGAMP^+^ cells containing cGAS^+^ micronuclei were observed (yellow outlines in Fig. 4a(top)), most FRET^+^ cells lacked cGAS^+^ micronuclei (cyan outlines in Fig. 4a, see also Fig. 4b,c). Conversely, cells with cGAS^+^ micronuclei often showed no evidence of cGAMP production (green outlines in Fig. 4a(top), see also Fig. 4c). Accordingly, cGAS activation occurred at similar frequencies in cells with and without cGAS^+^ micronuclei (Fig. 4c). Given that cGAS activation by IR can occur in clusters, it is possible that micronucleated cells might transfer cGAMP to non-micronucleated cells. However, the distance to the closest cell with a cGAS^+^ micronucleus was indistinguishable between FRET^−^ and FRET^+^ cells (Extended Data Fig. 6a). Moreover, FRET^+^ clusters containing at least one cGAS^+^ micronucleated cell did not contain more cells than clusters that did not contain cGAS^+^ micronuclei nor did they contain cells with higher FRET signal (Extended Data Fig. 6b). Finally, cells with cGAS^+^ micronuclei were not more frequent in activated clusters than randomized populations (Extended Data Fig. 6c). Together with the finding that most cells containing cGAS^+^ micronuclei did not activate cGAS (Fig. 4c), this provides an argument against micronuclei being major contributors to cGAS activation by IR.

Given that radiation generates micronuclei by potentially complex processes and may have other effects on cellular physiology besides micronucleation, we also generated micronuclei by promoting chromosome mis-segregation independently of previous DNA damage. This was achieved by treatment with MPS1 inhibitors (MPS1i), which weaken the spindle-assembly checkpoint^56,57^. We initially used 72 h continuous treatment with 0.5 μM reversine—conditions commonly used to generate micronuclei^43,56^—but in addition to micronuclei, this caused extensive cell death and nuclear abnormalities (Fig. 4d). As this precluded the investigation of potential correlations between micronuclear enrichment of cGAS and its activation, we used lower reversine concentration pulses instead, which generated cGAS^+^ and cGAS^−^ micronuclei but did not promote gross nuclear changes (Fig. 4e,f and Extended Data Fig. 6d). Although reversine treatment did promote cGAS activation, this was again limited to a subset of cells (≤20%), even at concentrations that caused gross abnormalities (Fig. 4g,h), indicating that the genotoxic stress generated by MPS1 inhibition also is not a potent cGAS stimulant. Similar results were obtained with another MPS1i, AZ3146 (Extended Data Fig. 6e). As for IR, reporter signal after reversine treatment depended on cGAS activity (Extended Data Fig. 6f) and almost all reversine-treated cells remained capable of activating cGAS following DNA transfection (Extended Data Fig. 6g). Correlative live FRET imaging, followed by fixation and cGAS immunofluorescence revealed that, similar to IR, the large majority of cells that activated cGAS did not contain a cGAS^+^ micronucleus (Fig. 4i). Furthermore, most cells with a cGAS^+^ micronucleus did not activate cGAS (Fig. 4i).

Also similar to IR, cGAS activation following reversine treatment frequently occurred in clusters (Extended Data Fig. 6h). However, FRET^+^ cells were not located closer to cells harbouring a cGAS^+^ micronucleus than FRET^−^ cells (Extended Data Fig. 6i). Furthermore, clusters containing a cGAS^+^ micronucleus were neither larger nor characterized by elevated cGAMP production (Extended Data Fig. 6j), and FRET^+^ clusters were no more likely to contain a cGAS^+^ micronucleus-bearing cell than randomized control populations (Extended Data Fig. 6k).

To directly assess whether micronuclear cGAS association temporally correlates with cGAMP production, we stably expressed cGAS fused to the bright monomeric red fluorescent protein mScarlet3 (ref. ^58^) in the reporter cell line (mScarlet3–cGAS; Extended Data Fig. 6l), which did not impact on the responder fraction after reversine treatment (Extended Data Fig. 6m). Time-lapse imaging after reversine treatment revealed that cGAS enrichment within micronuclei was not associated with subsequent cGAMP generation (Fig. 4j,k and Supplementary Video 4), even after multiple hours. Analysis of the history of first cells activating cGAS in a cluster and isolated cells activating cGAS furthermore showed that transient enrichment within micronuclei was also not a requirement for cGAS activation after IR or reversine treatment (Fig. 4l–n). We conclude that micronuclear cGAS enrichment does not generally lead to cGAS activation and that cGAS activation can occur independently of micronuclei.

### Chromosome bridges correlate with cGAS activation after chromosome mis-segregation but not ionizing radiation

**Fig. 5:**
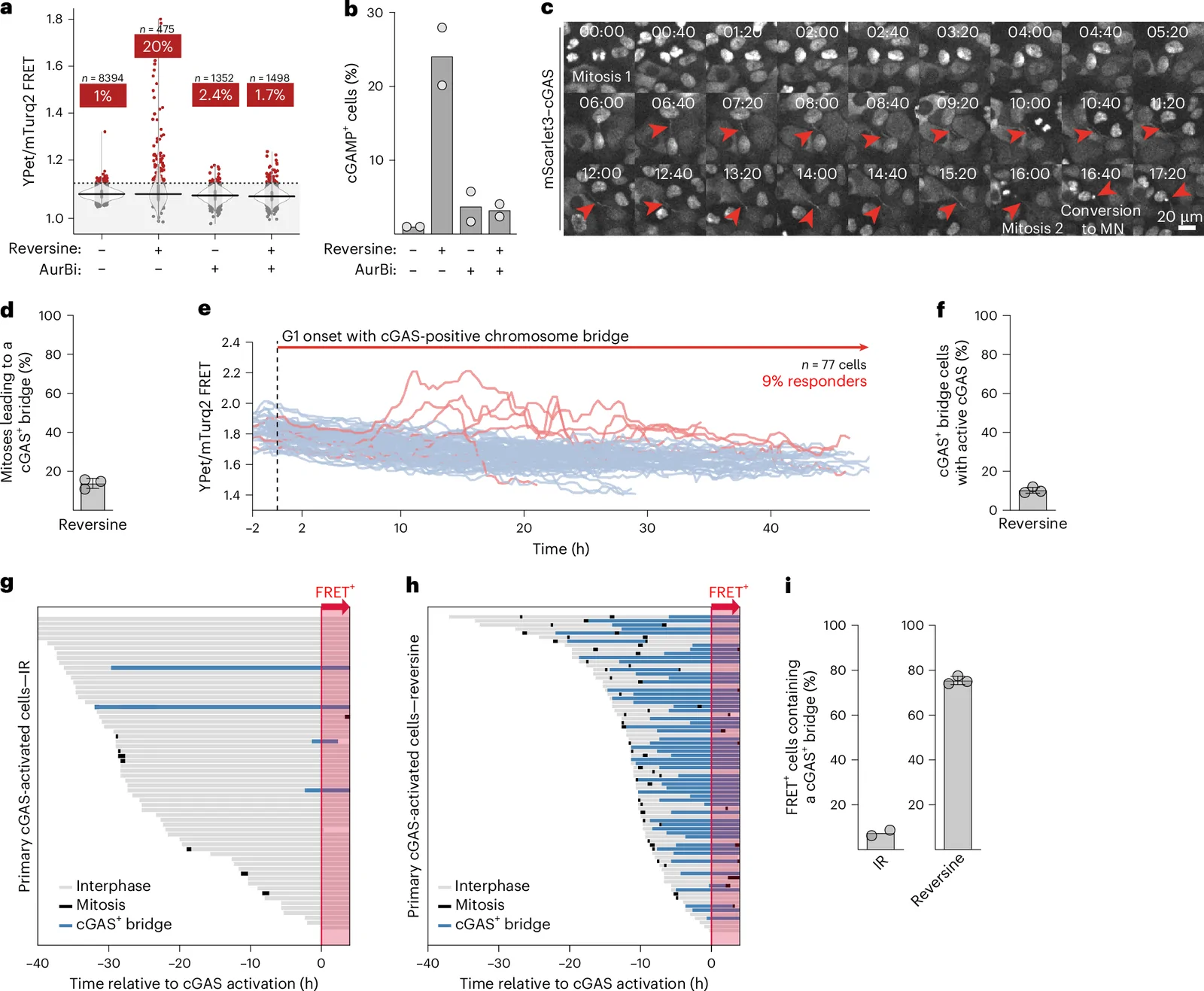
Chromosome bridge enrichment correlates with cGAS activation in a stimulus-dependent manner. **a**,**b**, MCF10A cells stably expressing the cGAMP reporter were either untreated or subjected to the indicated treatments for 72 h, followed by microscopy analysis. Results from an individual experiment (**a**) and averages from two independent experiments (**b**). **a**, The dots represent individual cells. The violin plots show the general distribution, with the median indicated by a horizontal line and the interquartile range indicated by a grey vertical line. Red dots indicate cells containing cGAMP (as defined by a FRET response higher than 99% of control cells). The numbers in the red boxes indicate the percentage of cells containing cGAMP. **c**–**f**, Time-lapse imaging analysis of mScarlet3–cGAS enrichment on chromosome bridges and cGAMP-reporter FRET of cells treated with 0.5 μM reversine. MN, micronucleus. **c**, Behaviour of a representative pair of daughter cells at the time points indicated up to 17 h 20 min. The arrowhead indicates formation of a cGAS+ bridge followed by conversion to cGAS+ MN at 16 h 40 min. **d**,**f**, Proportion of cells forming cGAS^+^ bridges following mitosis (**d**) and proportion of cells with cGAS^+^ bridges in which cGAS activation occurred (**f**). Data are the average ± s.d. of three independent experiments. **e**, Representative traces from one experiment (each trace represents FRET values over time for individual cells; cells that activate cGAS are displayed in red, inactive cells in blue). Cells were synchronized computationally to the time of entry into G1. **g–i**, Lineage analysis of cells irradiated with 15 Gy (**g**) or treated with 0.5 μM reversine (**h**). The behaviour of each cell activating cGAS in a representative experiment for each condition is shown (**g**, *n* = 64 cells, data from one of two independent experiments; **h**, *n* = 76 cells, data from one of three independent experiments. Note that these cells are the same as the ones analysed for Fig. 4l–n). Isolated responder cells, or first responder cells in clusters were identified and back-traced throughout the time lapse. **i**, Percentage of responder cells treated with IR (left) or reversine (right) that had a cGAS^+^ chromosome bridge. Data are the mean ± s.d. of two (IR) and three (reversine) experiments. Source numerical data are provided. Source data

**Extended Data Fig. 7:**
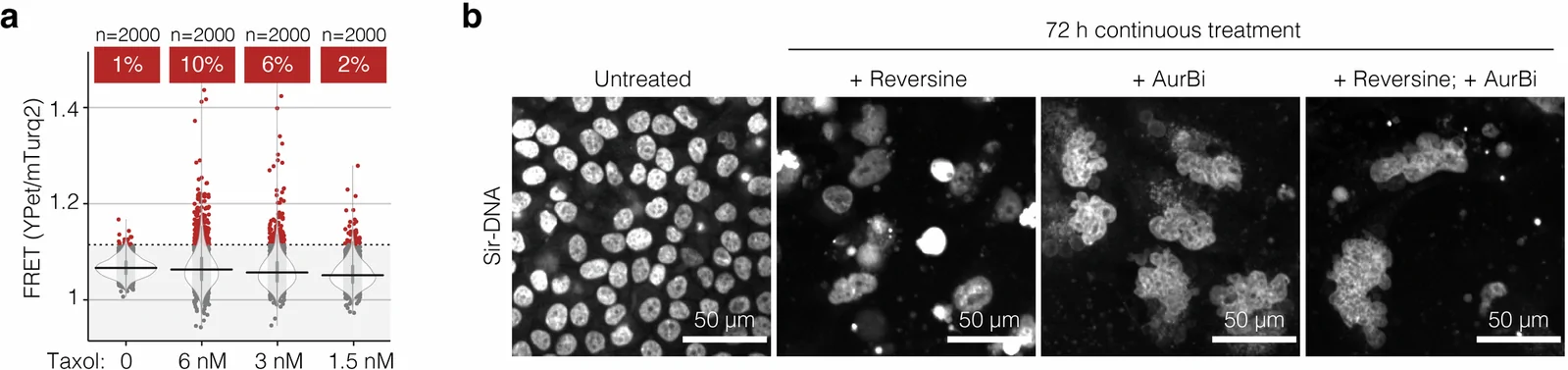
Impact of taxol treatment and AurBi treatment on cGAS activation. MCF10A cells stably expressing the cGAMP reporter were either untreated or subjected to the indicated treatments. **a**, MCF10A stably expressing the cGAMP reporter were treated for 48 h with the indicated dose of the taxane Taxol, and subsequently imaged for FRET ratio measurement. Dots represent values from individual cells expressing the cGAMP reporter. Violin plots show the general distribution, with the median indicated by a horizontal line, and the interquartile range indicated by a grey vertical line. Red dots indicate cells containing cGAMP (as defined by a FRET response higher than 99% of control cells). Numbers in red boxes indicate the percentage of cells containing cGAMP. Shown is one of three independent experiments. **b**, Analysis of nuclear shape following 72 h of treatment with the indicated drugs. Shown are images from one out of two independent experiments. Source numerical data are provided.

Previous research indicated that, among antimitotic drugs, MPS1i and taxanes might be especially potent at promoting cGAS activation^24^. This was proposed to be mediated by cGAS enrichment on chromosome bridges, which MPS1i and taxanes more frequently promote than other antimitotic drugs^24^. In addition, Aurora B inhibitors^59^ (AurBi) were reported to reduce MPS1i-dependent cGAS signalling, interpreted as a consequence of reducing chromosome-bridge frequency^24^. To investigate this, we first tested predictions from these findings in our experimental set-up. Taxane treatment indeed promoted dose-dependent cGAS activation in some cells (Extended Data Fig. 7a), and reversine-dependent cGAS activation was reduced by co-treatment with the AurBi ZM447439 (Fig. 5a,b). Furthermore, the morphological changes promoted by AurBi were epistatic to those caused by reversine (Extended Data Fig. 7b).

To directly test whether cGAS enrichment on chromosome bridges was a major driver of cGAS activation, we analysed time-lapse images of reversine-treated cells expressing mScarlet3–cGAS. Fewer than 20% of the cells formed cGAS^+^ chromosome bridges (Fig. 5c,d). Among the cells with cGAS^+^ bridges, cGAS activation occurred in only about 10% of cases (Fig. 5e,f), indicating that most cells in which cGAS associates with a bridge do not activate cGAS. Strikingly, however, there was a contribution of chromosome bridges in the reversine-treated cells (but not irradiated cells) that became apparent when we focused only on cells that activated cGAS (either in isolation or as first cells in a cGAMP^+^ cluster): the majority (approximately 70%) of these cells had experienced a cGAS^+^ chromosome bridge (Fig. 5g–i). No such correlation was observed for IR (Fig. 5g,i). We conclude that although cGAS enrichment on chromosome bridges is neither necessary nor sufficient for its activation, specific genotoxic stressors may generate a distinct subset of bridges that actively promotes cGAS activation (Discussion).

### Model R-loops, but not DNA:RNA hybrids, promote cGAS activation

**Fig. 6:**
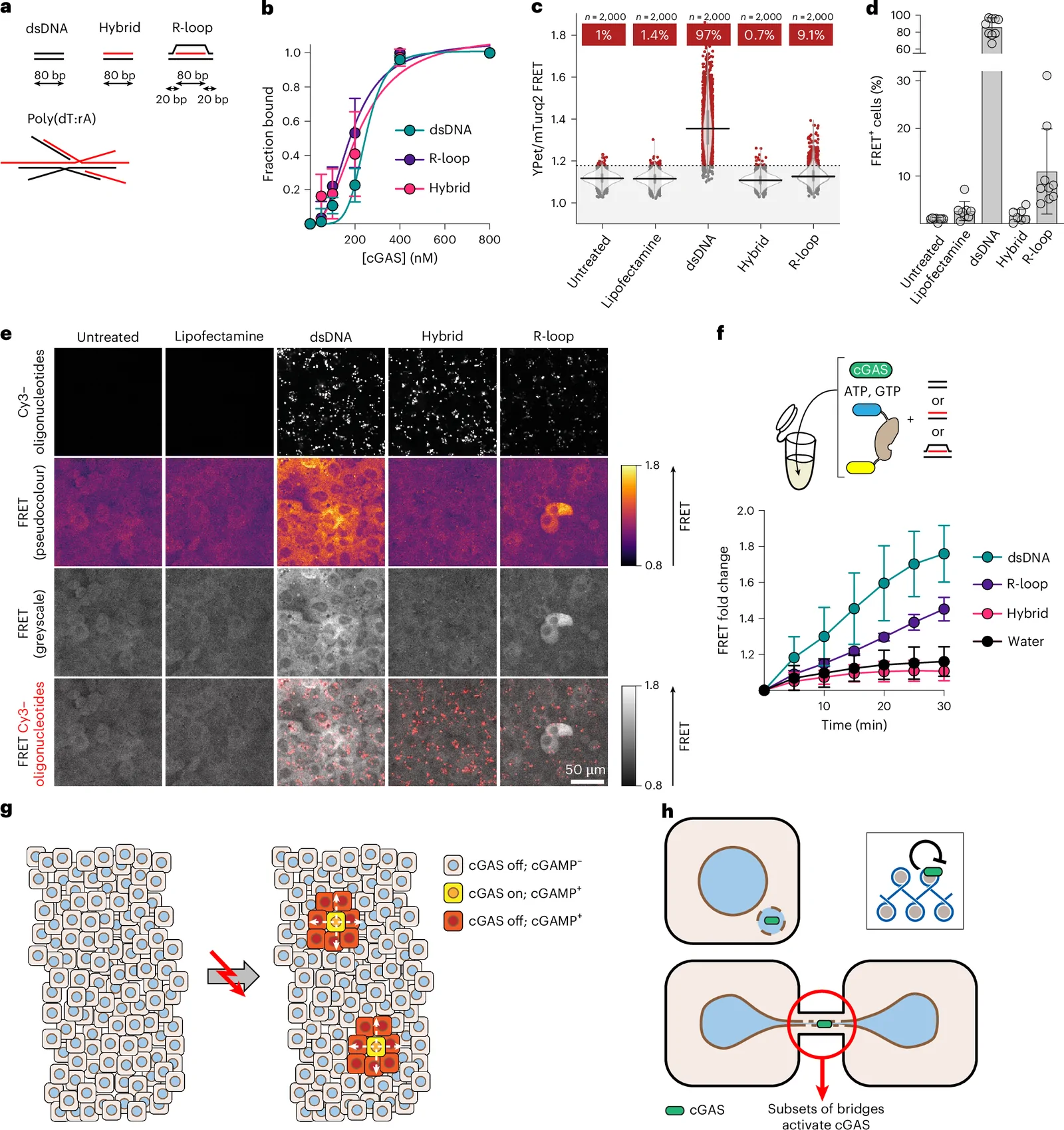
Model R-loops can activate cGAS. **a**, Schematic of the model structures used. DNA strands are shown in black and RNA strands in red. **b**, Gel mobility shift binding analysis with purified cGAS and the indicated structures. Average ± s.d. of three experiments. **c**–**e**, MCF10A cells stably expressing the cGAMP reporter were either untreated or transfected with the indicated structures, followed by microscopy analysis after 24 h. **c**,**d**, Individual experiment (**c**) and averages of nine independent experiments (**d**; error bars, s.d. Note that experiments using unlabelled and Cy3-labelled substrates were pooled). **c**, The dots represent individual cells. The violin plots show the general distribution, with the median indicated by a horizontal line and the interquartile range indicated by a grey vertical line. Red dots indicate cells containing cGAMP (as defined by a FRET response higher than 99% of control cells). The numbers in the red boxes indicate the percentage of cells containing cGAMP. **e**, Microscopy analysis showing cGAMP-reporter FRET as well as signal from the indicated Cy3-labelled structures. Images are representative of five independent experiments. **f**, In vitro reconstitution of cGAMP generation by cGAS and the indicated structures using the cGAMP reporter as a readout. Average ± s.d. of three experiments. **g**,**h**, Working model for cGAS responses in tissues and cells experiencing genotoxic stress. **g**, When a tissue experiences genotoxic stress, such as that associated with radiotherapy and chemotherapy, cGAS–STING signalling and IFN production may be localized events that could, however, spread through cGAMP transfer and cell migration. Cells that receive cGAMP from neighbouring cells are shown in red. **h**, In individual cells, micronuclei (top left) and chromosomal bridges (bottom left) are generally not potent drivers of cGAS activation, potentially due to inhibition by nucleosomes (top right). However, subsets of chromosome bridges could activate cGAS and these bridges could have different likelihoods of generation depending on the type of genotoxic stress. Source numerical data are provided. Source data

**Extended Data Fig. 8:**
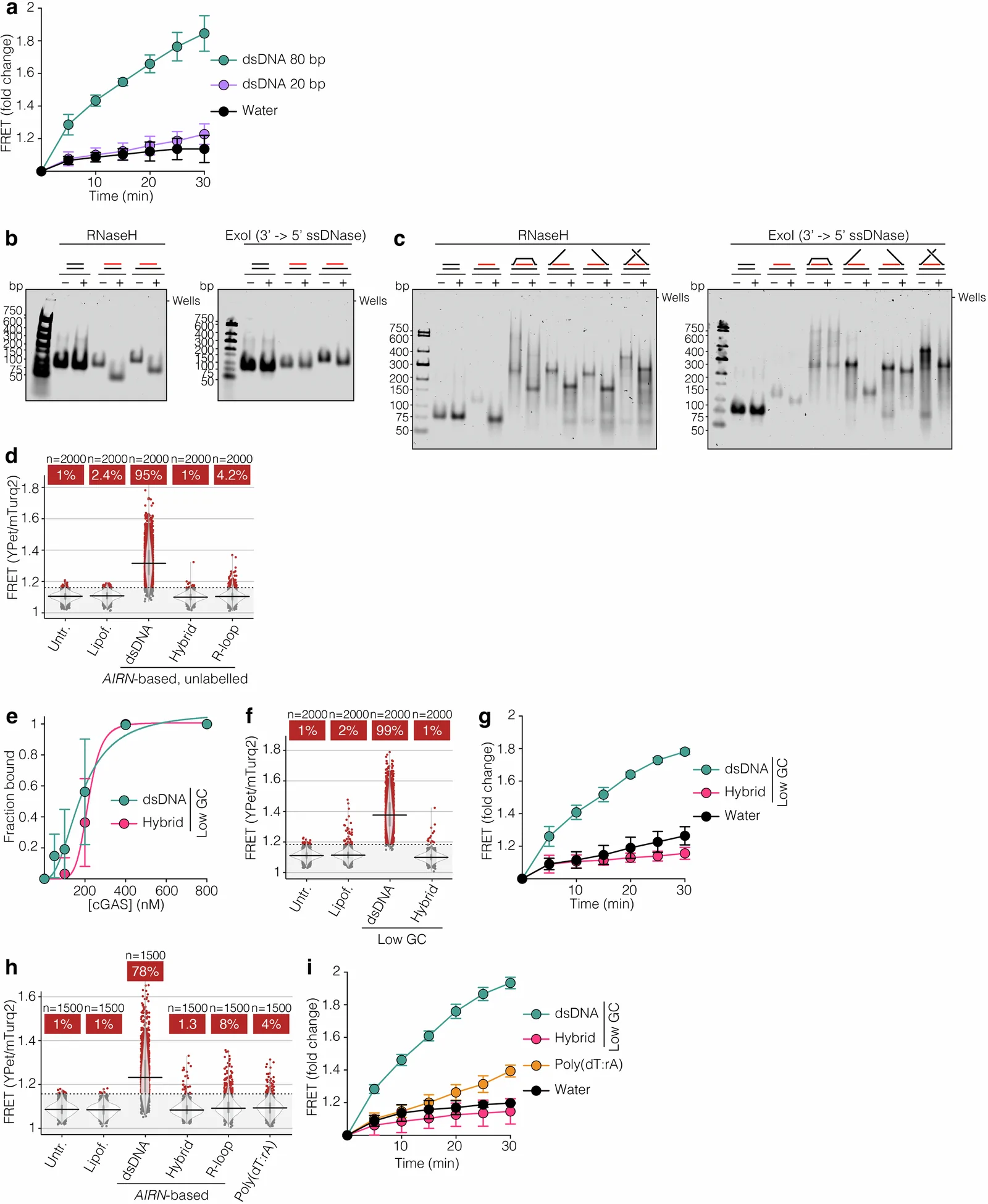
Further characterization of the cGAS response to DNA:RNA hybrids and R-loops. **a**, Analysis of cGAS activation by 20 bp or 80 bp dsDNA olignonucleotides by biochemical analysis using the cGAMP FRET reporter as a readout as in Fig. 1l. Averages and standard deviations from three independent experiments are shown. **b**,**c**, Validation of the DNA structures used for the analysis of cGAS activation. Folded structures were treated with the indicated enzymes, and products were resolved by native polyacrylamide gel electrophoresis. Bands were visualized using SYBR Gold. Red lines indicate RNA, black lines indicate DNA. **d**, MCF10A cells stably expressing the cGAMP reporter were either untreated, mock transfected with Lipofectamine (Lipof.), or transfected with the indicated structure. Dots represent values from individual cells expressing the cGAMP reporter. Violin plots show the general distribution, with the median indicated by a horizontal line, and the interquartile range indicated by a grey vertical line. Red dots indicate cells containing cGAMP (as defined by a FRET response higher than 99% of control cells). Numbers in red boxes indicate the percentage of cells containing cGAMP. Shown is one of three independent experiments. **e**–**g**, Analysis of cGAS activation by lower GC content 80 bp dsDNA or DNA:RNA hybrids. **e**, Gel mobility shift binding analysis with purified cGAS and the indicated structures. Averages and standard deviation from four experiments are shown. **f**, MCF10A cells stably expressing the cGAMP reporter were either untreated, mock transfected with Lipofectamine (Lipof.), or transfected with the indicated structure. Graph parameters as in **d**. Shown is one of four independent experiments. **g**, In vitro reconstitution of cGAMP generation by cGAS and the indicated structures using the reporter as a readout. Averages and standard deviations from three independent experiments are shown. **h**,**i**, Analysis of cGAS activation by poly(dT:rA). **h**, MCF10A cells stably expressing the cGAMP reporter were either untreated, mock transfected with Lipofectamine (Lipof.), or transfected with the indicated structure. Graph parameters as in **d**. Shown is one of four independent experiments. **i**, In vitro reconstitution of cGAMP generation by cGAS and the indicated structures using the reporter as a readout. Averages and standard deviations from three independent experiments are shown. Source numerical data and unprocessed blots are provided.

Early studies based on annealed poly(dT) and poly(rA) polymers indicated that DNA:RNA hybrids might have a weak stimulatory effect on cGAS, suggesting a mechanism by which cGAS could respond to retroviruses and retroelements^31^. These findings were recently extended with the possibility that DNA:RNA hybrids released from processed R-loops may promote cGAS activation^28^. Given that R-loops frequently result from genotoxic stress, can themselves drive further genotoxic stress^29,30^ and may be released by micronuclei^60^, they could be important cGAS activators. To test this, we generated model dsDNA, DNA:RNA hybrid and R-loop substrates based on the endogenous R-loop region at the murine *AIRN* locus^61,62^, with the displaced R-loop strand optimized to show no homology to the bottom strand (Fig. 6a). The flanking dsDNA regions were designed to be too short to activate cGAS themselves^63^ (Extended Data Fig. 8a). Annealing procedures were optimized to generate largely homogenous structures (Methods). Diagnostic digestion with RNaseH (which degrades the RNA of DNA:RNA hybrids) or ExoI (which degrades single-stranded DNA in the 3′ to 5′ direction), and comparison to flap versions of the R-loop confirmed correct folding (Extended Data Fig. 8b,c).

Surprisingly, purified cGAS bound all these structures with equal affinity, as determined by electrophoretic mobility shift assays (Fig. 6b). However, when transfected into cells, DNA:RNA hybrids were inert (Fig. 6c,d), whereas the R-loop activated cGAS in a fraction of cells, reminiscent of our observations with DNA damage (Fig. 6c,d). As expected, the dsDNA version activated cGAS in all cells (Fig. 6c,d). Incorporation of a Cy3 label ruled out poor delivery as the cause of hybrid inertness (Fig. 6e) and the fluorophore itself did not affect the responder fraction (Extended Data Fig. 8d). These results were mirrored in biochemical assays using purified cGAS and cGAMP reporter, which again showed dsDNA to be a potent cGAS activator, DNA:RNA hybrids to be inert and the R-loop to have an intermediate effect (Fig. 6f). To confirm that the inertness of the DNA:RNA hybrid was not an artefact of the high GC content that characterizes *AIRN* similar to most R-loops, we generated dsDNA and DNA:RNA hybrids based on an 80 bp sequence of lower GC content previously shown to activate cGAS as dsDNA^63^. Once again, cGAS bound both versions with equal affinity (Extended Data Fig. 8e) but was only activated by the dsDNA version in both cellular and biochemical assays (Extended Data Fig. 8f,g). To compare these findings with previous work on DNA:RNA hybrids^31^, we annealed dT and rA polymers as described previously^31^. Note that rather than forming linear poly(dT:rA) hybrids, these are likely to form complex branched structures (Fig. 6a). In agreement with a previous study^31^, mild stimulation by poly(dT:rA) hybrid networks was detected in cellular and biochemical assays (Extended Data Fig. 8h,i). This indicates that DNA:RNA hybrids can stimulate cGAS, but potentially only when they occur in conjunction with more complex secondary structures. Therefore, although clean DNA:RNA hybrids are unlikely to be major drivers of cGAS activation, more complex hybrids such as those found at R-loops, can activate cGAS. Together, our work establishes that the local enrichment of cGAS cannot be taken as an indicator of its activation status, and that subsets of chromosome bridges and complex structures containing DNA:RNA hybrids can promote cGAS activation.

## Discussion

Although pathogen DNA is likely to activate cGAS during infection, it remains unclear how cGAS is activated by genotoxic stress and whether this occurs through multiple pathways or a common underlying mechanism. Here we present an improved FRET-based reporter for cGAMP, with which we addressed these questions. This reporter can be used in microscopy, biochemical and flow cytometry assays.

In potential contrast to the STING CDN on its own, which was dimeric in structural studies performed in the absence of cGAMP^64–66^, the reporter only dimerized when bound to cGAMP. This difference might be due to the high CDN concentrations used in structural studies (micromolar to millimolar concentrations, and higher), which might promote dimerization, whereas our analysis was conducted at low concentrations (14 nM). In agreement with this, a CDN construct that was dimeric in crystals without cGAMP^64^ was also monomeric in solution under low concentrations (Extended Data Fig. 1f,g). Although nanomolar concentrations may seem more physiological than the high concentrations of structural biology, STING membrane association could generate local concentrations sufficient for cGAMP-independent dimerization.

Careful cell-line construction and experimental design will ensure robust results when using our cGAMP reporter. Cells with low cGAMP levels might require low reporter expression and knockdown/knockout of endogenous STING may further improve signal. Reduced reporter expression levels may ensure that it does not reduce the availability of cGAMP for endogenous STING. Importantly, however, neither procedure was necessary in our set-up.

It is unlikely that our reporter intercalates into endogenous STING oligomers as it does not intrinsically oligomerize and shows no evidence of intracellular aggregation (for example, Figs. 1c, 2a and 3f,i,j). Presumably because it lacks the transmembrane domain, the reporter fails to enrich at the Golgi apparatus, where cGAMP-bound STING is normally trafficked^66–71^ and targeted for degradation^69,72–78^. This, combined with the potentially low cGAMP off-rate, suggests that the reporter may show delayed off-kinetics, which may allow cGAMP production to be captured cumulatively, facilitating detection of weak or transient events. Given that different STING agonists showed variable activation/dissociation kinetics (Fig. 2a,b and Extended Data Fig. 2g), our reporter may help inform agonist choice for specific experimental contexts.

Other potential single-cell approaches to determine cGAS activation have been described, such as visualizing IFN-β expression, ISG expression^21,22^, nuclear IRF3 accumulation^34^, the subcellular translocation of STING^33^ or of a STING–IRF3 fusion construct^35^. However, IFN-β expression, ISG expression and IRF3 translocation are not unique to cGAS^36,79^ and are under feedback control, showing transient activation when continuously stimulated (Extended Data Fig. 3d,e). In addition, many cancer cell lines rewire cGAS signalling away from IRF3 (ref. ^80^). Similar to IRF3 translocation, STING translocation is transient due to activation-dependent degradation^37^. Finally, all these approaches require intact downstream signalling and may themselves change downstream signalling^35^. In contrast, our reporter visualizes cGAMP generation, which is unique to cGAS and the most upstream step in the pathway. It is also more sensitive than these other approaches and can be used without impacting downstream signalling.

In response to DNA damage and chromosomal instability, cGAS activation was primarily investigated at the population level, interpreted as uniform responses of cells during genotoxic stress. However, we found that cGAS activation induced by multiple genotoxic agents is a rare event that moreover does not frequently result in high cGAMP production. This was also supported by our own population-based gene expression analysis. Revisiting previous work in this light suggests that low-frequency cGAS activation may be a universal aspect of genotoxic stress^6,8,11,21,22,43,81^. Our results therefore indicate that within exposed tissue, local signalling centres may exist that can expand by cGAMP transfer and cell migration (Fig. 6g). This may be especially relevant within tumours, where locally restricted signalling may promote tumour microenvironment heterogeneity^82^.

Our findings also provide a possible explanation for the conclusions of a recent study based on nuclear translocation of IRF3 that questioned whether cGAS could be activated after DNA damage and chromosomal instability^34^. Our reporter shows that cGAS activation does occur during genotoxic stress, albeit in an attenuated and rare manner, which may be too weak and asynchronous to be detected through IRF3 translocation.

Testing a major hypothesis for how cGAS is activated by genotoxic stress, we showed that cGAS enrichment on micronuclei does not generally lead to cGAMP production (Fig. 6h). Similar results were recently reported using other, albeit less optimal, visualization of cGAS signalling^24,33–35^. This lack of activation probably reflects the fact that micronuclei are composed of chromatin^25–27^, which would keep cGAS inactive^6,9–16^. Even chromosome 21, the smallest human chromosome^83^, would contain a large excess of nucleosomes over the approximately 50,000 cGAS molecules estimated to be present in a relatively high-expressing cell line such as HeLa^6,48^. Although our work does not exclude that micronuclear cGAS can be activated, it indicates that enrichment is insufficient and that other processing events may be required.

Similar to micronuclei, we show that chromosome-bridge enrichment of cGAS—also suggested as a major cGAS activator^24^—is unlikely to generally lead to cGAMP production. However, subsets of bridges, or bridges co-occurring with other yet unidentified cellular events, may activate cGAS, as indicated by our data (Fig. 6h). These findings also imply that not all genotoxic stresses are likely to activate cGAS in the same way, given that only MPS1i (not IR) showed bridge-associated cGAS activation. Although the exact nature of the structures activating cGAS is unclear, processing of DNA damage to more complex structures such as R-loops or branched molecules may be involved, as indicated by our data. The generation of these will probably also be affected by cell-cycle events, which regulate many aspects of DNA-damage processing. Our cGAMP reporter will provide a useful tool to identify these structures and the processes generating them.

## Methods

### Cells

MCF10A cells were cultured in DMEM/F12 medium supplemented with 5% (vol/vol) horse serum (Thermo Fisher Scientific), 20 ng ml^−1^ EGF (Sigma-Aldrich), 0.5 μg ml^−1^ hydrocortisone (Sigma-Aldrich), 100 ng ml^−1^ cholera toxin (Sigma-Aldrich), 10 μg ml^−1^ insulin (Sigma-Aldrich) and 1% penicillin–streptomycin (Gibco). SUM149PT and SUM149 BRCA1-revertant cells were cultured in Ham’s F12 medium (Gibco) supplemented with 5% (vol/vol) heat-inactivated fetal bovine serum (FBS; Gibco), 10 μg ml^−1^ insulin (Sigma), 0.5 μg ml^−1^ hydrocortisone (Sigma) and penicillin–streptomycin. HeLa, MDA-MB-231 and HEK293FT cells were cultured in DMEM medium supplemented with 10% FBS and 1% penicillin–streptomycin.

MCF10A cells were a gift from J. Mansfeld^84^. HEK293FT cells were provided by K. Helin (ICR London, UK). SUM149PT cells were provided by C. Lord (ICR London, UK). The MDA-MB-231 and Hela Flp-IN T-Rex H2B–RFP cGAS-knockout cells, and HeLa Flp-IN T-Rex H2B–RFP cGAS-knockout cells with EGFP-cGAS rescue were previously generated by us^6^.

### Cell-line generation

MCF10A cells stably expressing the cGAMP reporter, the cGAMP-blind reporter, GFP–IRF3 and mScarlet3–cGAS were generated by lentivirus transduction. To generate lentiviral particles, pLex306 plasmid (Addgene plasmid, 41391) containing the open reading frames of either sequence was co-transfected with psPAX2 and pMD2.G into HEK293FT cells at 50% confluency in a T75 flask using Lipofectamine 2000 (Invitrogen). The medium was changed after 24 h, and the supernatant was collected after three days and centrifugated to remove cell debris. The supernatant was mixed 3:1 with 4×concentrator solution (80 g PEG-8000, 14 g NaCl in 200 ml 1×PBS, pH 7.0–7.2) and mixed overnight by constant rotation at 4 °C. The mix was centrifuged at 1,600*g* and 4 °C for 60 min. The pellet was resuspended in PBS and used to transfect MCF10A cells in T25 flasks. Successfully transduced clones were selected by flow cytometry based on expression levels.

MCF10A cGAS^−/−^ cells stably expressing inducible mScarlet3–NLS–cGAS R255E were generated by lentivirus transduction. To generate lentiviral particles, pLX-TRE plasmid containing Tet-ON 3G as well as the open reading frames of mScarlet3–NLS–cGAS R255E under the control of a TRE3G promoter was co-transfected with psPAX2 and pMD2.G in HEK293FT cells as described in the previous paragraph. The final pellet, resuspended in PBS, was used to transfect MCF10A cGAS-knockout cells stably expressing the cGAMP reporter in T25 flasks. Positive cells were selected by blasticidin (10 μg ml^−1^) for a week.

SUM149PT and SUM149 BRCA1-revertant cells stably expressing the cGAMP reporter and the cGAMP-blind reporter were generated by lentivirus transduction. To generate lentiviral particles, pLex306 plasmid containing the open reading frame of either was co-transfected with psPAX2 and pMD2.G into HEK293FT cells at 50% confluency in a 10-cm dish using Lipofectamine 2000 (Invitrogen). The medium was changed after 24 h, and the supernatant was collected after three days, filtered with a 0.4-μm pore filter to remove cell debris and used to infect cells in suspension in a 3-cm dish. The infected cells were then expanded, sorted by flow cytometry and pools of transduced cells were generated.

Hela H2B–RFP cGAS-knockout and EGFP-cGAS rescue cells were used according to our established methods^6^. The cGAS knockouts and STING knockouts were generated by ribonucleoprotein delivery using EnGenCas9-NLS (NEB, M0646M), Alt-R CRISPR–Cas9 *trans*-activating CRISPR RNA (crRNA; IDT, 1072534), Alt-R CRIPSR–Cas9 electroporation enhancer (IDT, 28481474) and Alt-R CRISPR–Cas9 crRNAs (designed using CHOPCHOP^85^). Ribonucleoproteins were generated according to the manufacturer’s instructions and nucleofected using a Lonza 4D-Nucleofector X unit (Lonza SE cell line 4D-Nucleofector X kit S; programme DS-138). Single clones were generated by cell sorting or limited dilution plating and tested by western blotting. The cGAS crRNA targeted AAGTGCGACTCCGCGTTCAG and the STING crRNA targeted CGGTCGGCCCGCCCTTCACT.

MDA-MB-231 cells stably expressing the cGAMP reporter were generated by lentivirus transduction. To generate lentiviral particles, pLex306 plasmid containing the open reading frames of the reporter was co-transfected with psPAX2 and pMD2.G into HEK293FT cells at 50% confluency in a T25 flask using Lipofectamine 2000 (Invitrogen). The supernatant was collected after two days, filtered with a 0.4-μm pore filter to remove cell debris and used to infect cells seeded on a six-well dish one day before the transduction. The medium was changed 24 h after infection. The infected cells were then expanded and seeded for single-cell clone isolation.

### Reagents

Chemical compounds dissolved in sterile dimethylsulfoxide were used at the following concentrations unless indicated otherwise: DMXAA STING agonist (Invivogen, 10 μg ml^−1^), cGAS inhibitor (G150, Selleckchem, 10 μM), Reversine (Fisher Scientific, 0.5 μM), AZ3146 (Fisher Scientific), doxorubicin (Medchem Express), etoposide (Medchem Express, 10 μM), aurora B inhibitor (ZM447439, Medchem Express), paclitaxel (Cayman), doxycycline hyclate (Sigma, 1 μg ml^−1^), olaparib (Selleckchem, 10 μM) and rucaparib (Selleckchem, 10 μM). Chemical compounds dissolved in sterile water were used at the following concentration unless indicated otherwise: 2′3′-cGAMP (Invivogen, 10 μg per electroporation with 100,000 cells), diABZI compound 3 (Invivogen, 1 μM), carbenoxolone (Tocris) and ABCC1 inhibitor (MK-571, Sigma, 25 μM).

### Antibodies

The following antibodies were used at the indicated dilutions: anti-cGAS (D1D3G), 1:300 for immunofluorescence, 1:1,000 for western blots (Cell Signaling Technology (CST), 15102); anti-IRF3 (D6I4C), 1:500 for immunofluorescence (CST, 11904); anti-phospho-IRF3 Ser396 (4D4G), 1:100 for immunofluorescence (CST, 4947); anti-phospho-STAT1 Tyr701 (58D6), 1:300 for immunofluorescence (CST, 9197); anti-yH2AX Ser139 (JBW301), 1:500 for immunofluorescence (Millipore, 05-636-25UG05-636-25UG); anti-STING (D2P2F), 1:1,000 for western blots (CST, 13647); anti-STING (E9X7F), 1:1,000 for immunofluorescence (CST, 90947) and anti-α-tubulin (DM1A), 1:5,000 for western blots (Sigma, T9026).

### Western blotting

Cell pellets were harvested and lysed in 1×RIPA buffer (Merck, 20-188) supplemented with 1×protease inhibitor cocktail (Roche, 11873580001). The samples were diluted in 4×LDS sample buffer (Life Technologies, NP0008) containing 1×sample reducing agent (Life Technologies, NP0009) and boiled for 5 min at 95 °C before being subjected to gel electrophoresis and western blotting. For western blotting mScarlet3–cGAS, the reducing/boiling step was omitted to prevent chromophore hydrolysis. The Odyssey infra-red imaging system (Li-Cor M) was used for detection.

### Plasmid construction

To generate a FRET-based cGAMP reporter, PCR amplification of the coding sequences of mTurquoise2 (ref. ^39^), mSTING fragment^38^ and YPet^40^ were cloned by restriction digest on an ePB piggyBac backbone^86^. For the cGAMP-blind reporter, the mSTING fragment was replaced with the Y239S/T262A mutant^38^. To generate lentiviral plasmids containing the cGAMP reporter, a fragment containing mTurquoise2 and the mSTING CDN was amplified by PCR and cloned in a pLex306 backbone. The sequence of *E. coli* codon-optimized YPet from ref. ^44^ was synthesised as a gBlock (IDT) and cloned onto the C terminus of mSTING. DNA sequences for the reporter constructs are provided in Supplementary Table 1.

The GFP–IRF3 construct was made by PCR amplification of GFP–IRF3 from ref. ^6^ and cloning it into the lentiviral pLex306 backbone. The mScarlet3–cGAS (hereafter mScarlet–cGAS) construct was made by PCR amplification of plasmid containing human cGAS for protein expression^6^. The mScarlet3 was synthesized by IDT. These DNA fragments were inserted into the lentiviral pLex306 backbone.

Plasmids for inducible expression of mScarlet3–NLS–cGAS R255E were generated by first generating an mScarlet3–NLS synthetic construct according to previous designs^8^ (synthesised by IDT). Together with PCR-amplified cGAS, this was then cloned into pLX-TRE-dCas9-KRAB-MeCP2-P2A–mCherry_BSD (a gift from A. Radzisheuskaya, ICR). Subsequently, Arg255 of cGAS was mutated to Glu.

Detailed maps of all plasmids are available on request.

### Recombinant protein expression and purification

Wild-type and mutant reporters as well as the CDN of mouse STING previously used for crystallization^64^ were expressed in Rosetta (DE3) pLysS cells from pET15b plasmid backbones with an N-terminal 6×His tag. The cells were seeded into terrific broth (24 g l^−1^ yeast extract, 12 g l^−1^ bacto tryptone, 0.004% glycerol, 17 mM KH_2_PO_4_ and 72 mM K_2_HPO_4_) containing 0.4% glucose, 50 μg ml^−1^ carbenicillin and 34 μg ml^−1^ chloramphenicol, and incubated with shaking at 37 °C until they reached an optical density at 600 nm of approximately 0.8. Expression was induced with 0.25 mM isopropyl β-D-1-thiogalactopyranoside at 18 °C for 6 h. Cleared lysate was incubated with HisPur Ni-NTA resin (Fisher Scientific, 10038124) for 1 h at 4 °C and recombinant protein was eluted using elution buffer (50 mM Tris–HCl pH 7.5 at 4 °C, 200 mM NaCl, 300 mM imidazole and 5 mM β-mercaptoethanol). The eluted fractions were pooled and further purified on a 1 ml HiTrap FF Q column (VWR, 17-5053-01) using an AKTA FPLC system (Cytiva). Flow-through fractions were collected and concentrated before size-exclusion chromatography on a Superdex 200 10/300 GL gel filtration column (Cytiva) using gel filtration buffer (40 mM Tris–HCl pH 7.5, 100 mM NaCl and 0.5 mM Tris(2-carboxyethyl)phosphine). The purified proteins were diluted in gel filtration buffer containing glycerol to a final concentration of approximately 40% glycerol and stored at −20 °C.

### Mass photometry

All measurements were performed on an OneMP mass photometer (Refeyn Ltd). Glass slides (high precision microscope cover glasses, 24 mm × 50 mm; Marienfeld, 630-2186) were cleaned with water and isopropanol four times, and then dried using compressed air. A drop of emersion oil (Olympus, Immoil-F30CC) was placed on the objective and a glass slide with a gasket (Reuseable Culturewell gasket 3 mm diameter × 1 mm depth; Grace Bio-Labs, 103250) adhered to the glass was placed on top of the emersion oil. The instrument was calibrated by the addition of 15 μl protein standards from Native mark unstained protein standard (Thermo Fisher, LC0725) in buffer A (10 mM Tris–HCl pH 7.4, 10 mM KCl and 1.5 mM MgCl_2_). For calibration, the objective was focused through the buffer-free method. For cGAMP reporter readings, 100 nM reporter pre-incubated with 250 nM cGAMP for 15 min at 25 °C or 100 nM reporter control reaction was prepared. The objective was focused using the drop-wise dilution method by the addition of 13 μl buffer A and 2 μl volumes of the corresponding reactions. The mass photometry recording was started in Acquire MP immediately after gentle mixing of the samples. Recordings were made in the regular field of view for 60 s, with 1,000–4,000 particle landing events per movie. Analyses of each movie were performed using the mass photometry software Discover MP. Masses of the major forms of reporter ±cGAMP were estimated by fitting a Gaussian distribution into mass histograms and taking the value at the mode of distribution.

### Cell-lysate preparation for cGAMP measurement

HeLa H2B–RFP cells with cGAS knockout and rescue^6^ were plated (600,000 cells per well) in a six-well tissue-culture dish containing DMEM plus 100 ng ml^−1^ doxycycline. The medium was replaced 24 h later with fresh medium without antibiotics and the cells were transfected with a cocktail of 2.6 μg pBlueScript II Sk (+), 8.62 μl ViaFect (Promega, E4981) and 103 μl Opti-MEM (Gibco, 31985062). After 8 h, 1.2 × 10^6^ cells were harvested following trypsinization and two rounds of washes with 1×PBS. The samples were processed using established procedures^87^. Briefly, each cell pellet was resuspended in 200 μl of 80:20 methanol:water and incubated overnight at −80 °C. The samples were then subjected to two rounds of vortex, freeze–thaw cycles in liquid nitrogen, sonicated in an ice-water bath for 10 min and clarified by centrifugation at 21,000*g* and 4 °C for 20 min. Extracts were dried using a SpeedVac and reconstituted in 50 μl buffer A.

### cGAMP measurement in cell lysates

For measurements based on the cGAMP reporter, 500 nM reporter concentrations were used. Reconstituted cell lysate was incubated with the reporter for 15 min at 25 °C and transferred into 96-well half area black flat-bottom plates (Sigma-Aldrich, CLS3694-25EA). FRET was measured on a POLARstar Omega system (BMG Labtech) with mTurqoise2 detected with 430 nm (excitation)–480 nm (emission) filter pairs and YPet-FRET detected with 430 nm (excitation)–530 nm (emission) filter pairs. Gain adjustments were done for reporter without cGAMP for 430–480 nm and for reporter with saturating concentrations of cGAMP for 430–530 nm. FRET ratios were determined by dividing 530 nm emission values by 480 nm emission values. A standard curve was generated for each experiment.

The levels of cGAMP in lysates were determined using an ELISA kit from Arbor Assays as per the manufacturer’s instructions. Briefly, standards were prepared in buffer A. Desiccated extracts were reconstituted in 130 μl buffer A, 20 μl of which was used for measurement in a 50 μl reaction. The cGAMP concentration was interpolated through sigmoidal 4PL regression of the standard curve. Considering the 2.5-fold dilution, the amount of cGAMP produced per one million cells was calculated.

### Native gel electrophoresis

Purified reporter (1 μg) was incubated with or without 2.64 μM cGAMP at 25 °C for 15 min. The samples were mixed with 2 M sucrose at a 5:1 volume ratio and resolved on Mini-Protean TGX 4–15% gels (Bio-Rad, 4561086) in a buffer containing 25 mM Tris and 192 mM glycine (pH 8.3) at room temperature (1 h, 100 V). The gel was stained with GelCode blue safe protein stain (Thermo Fisher Scientific, 10763505) and imaged on an LI-COR Odyssey M infra-red scanner.

### Preparation of DNA substrates for cGAS activity assays and cell transfection

All oligonucleotides, as well as their source and manufacturer, are summarized in Supplementary Table 2. Oligonucleotides designed to form R-loops as well as their dsDNA and DNA:RNA hybrid equivalents were based on the endogenous R-loop-forming region at mAIRN and were chosen based on previous analysis of R-loop formation^61,62^. Iterative BLAST analysis was used to design the displaced top strand of the R-loop to ensure as little homology to the bottom strand as possible. Low-GC oligonucleotides were based on previously published 80-base pair sequences, shown to provide potent cGAS activation stimulation^63^.

The following oligonucleotides were used to generate mAIRN-based structures (Supplementary Table 2): dsDNA, OCZ815 + OCZ816; DNA:RNA hybrid, OCZ813 + OCZ816; DNA:RNA hybrid with short single-stranded DNA overhangs, OCZ813 + OCZ818; R-loop, OCZ813 + OCZ817 + OCZ818; 3′ flap, OCZ813 + OCZ818 + OCZ826; 5′ flap, OCZ813 + OCZ818 + OCZ827 and double flap, OCZ813 + OCZ818 + OCZ826 + OCZ827. For the low-GC version, the following oligonucleotides were used (Supplementary Table 2): dsDNA, OCZ836 + OCZ837 and DNA:RNA hybrid, OCZ837 + OCZ838. The 20 bp dsDNA oligonucleotides from Extended Data Fig. 8a were OCZ839 + OCZ840 (Supplementary Table 2). All oligonucleotides were synthesised by IDT.

To support the formation of R-loops over single flaps, we folded all these structures in the presence of 10 mM MgCl_2_, as suggested by a previous study^88^. Oligonucleotides were mixed (4 μM each) in 40 mM Tris-acetate (pH 8 at 22 °C) and 10 mM MgCl_2_, and annealed in a thermal cycler. After optimization, we found that the following settings produced the most homogenous results: 98 °C for 5 min, ramp to 60 °C at 0.81 °C min^−1^ over 47 min, 60 °C for 30 s, ramp to 55 °C at 0.81 °C min^−1^ over 6 min, 55 °C for 30 s, ramp to 50 °C at 0.81 °C min^−1^ over 6 min, 50 °C for 30 s, ramp to 25 °C at 0.81 °C min^−1^ over 31 min, and finally incubate overnight at 6 °C. Following annealing reactions, 1 μl was mixed with 1 μl water and 0.4 μl of 25% glycerol, resolved on a native 4.5% polyacrylamide gel in 0.5×TBE, stained with SYBR Gold (Thermo Fisher Scientific) and visualized using a Li-Cor M Odyssey infra-red imaging system. For generating poly(dT:rA), we followed established procedures^31^ with slight modifications. Poly(dT) (Sigma, P6905) and poly(rA) (Sigma, P9403) were resuspended at 0.4 mg ml^−1^ in nuclease-free water. Subsequently, they were mixed at a concentration of 100 ng μl^−1^ each in 40 mM Tris-acetate (pH 8 at 22 °C) and 10 mM MgCl_2_, and annealed using the following temperature steps: 98 °C for 5 min, ramp to 20 °C at 0.75 °C min^−1^ over 105 min and overnight incubation at 4 °C. The dsDNA oligonucleotides used in Extended Data Fig. 8a were prepared by mixing top and bottom strands (1 μM each), which were annealed with the same temperature steps used to make dsDNA, DNA:RNA hybrids and R-loops. All substrates were stored at −20 °C until use.

For analysis of these substrates with nucleases, 1 μl of the relevant annealing mixes was digested with 0.4 μl exonuclease I (NEB, M0293S) or 0.4 μl RNaseH (NEB, M0297S) in a total volume of 8 μl containing the respective reaction buffers as recommended by the manufacturer for 30 min at 37 °C. Following the addition of 2 μl of 35% glycerol, the products were resolved on a native 4.5% polyacrylamide gel in 0.5×TBE, stained with SYBR Safe (Thermo Fisher Scientific) and visualized using a Li-Cor M Odyssey infra-red imaging system.

### Biochemical reconstitution of cGAMP production by cGAS

Each reaction consisted of 20 μl in a reaction buffer of 16 mM Tris–HCl pH 7.5 at 22 °C, 60 mM NaCl, 6 mM MgCl_2_, 0.8 mM dithiothreitol, 1 mM ATP and 1 mM GTP. cGASΔN lacking the N-terminal domain, which is not required for catalytic activity^3,89–91^, was purified as described previously^6^ and used at 250 nM. The cGAMP reporter was used at 500 nM. Double-stranded DNA, DNA:RNA hybrids and R-loops were used at 100 nM each. Where applicable, cGAMP was used at 1 μM. For each reaction, cGAS, reporter and (where applicable) DNA, DNA:RNA hybrid or R-loop were mixed in 18 μl and pre-incubated for 20 min. Subsequently, this mix was added to a well of a low-volume flat-bottom black 384-well plate (Corning, 3821) already containing 2 μl of 10×ATP/GTP mix. Measurements (430 nm excitation and 480 nm emission; 430 nm excitation and 530 nm emission) were performed every 5 min in a POLARstar Omega plate reader (BMG Labtech) pre-warmed to 25 °C. The FRET ratio was calculated by dividing the 530 nm emission result by the 480 nm emission result and plotted normalized to the starting value for each sample.

### cGAS binding assays

Binding assays were performed in 20 mM Tris–HCl (pH 7.5 at 22 °C), 50 mM NaCl, 5 mM MgCl_2_, 1 mM dithiothreitol and 6% glycerol using 50 nM dsDNA, DNA:RNA hybrid or R-loop and full-length cGAS of the indicated concentration. Binding mixes (8 μl total) were incubated at 24 °C for 60 min, resolved on a native 4.5% polyacrylamide gel in 0.5×TBE, stained with SYBR Gold (Thermo Fisher Scientific) and visualized using a Li-Cor M Odyssey infra-red imaging system. ImageJ was used to quantify the fraction bound at each concentration of cGAS.

### Analysis of CCL5 expression

For irradiated samples, approximately 2 × 10^5^ cells were seeded in a well of a six-well plate. The cells were irradiated with 15 Gy following day. Immediately after irradiation, the medium was replaced with fresh medium. The cells were harvested for RNA extraction 48 h post irradiation. For DNA transfection samples, 4 × 10^5^ cells were seeded in a well of a six-well plate. The following day, the medium was removed and replaced with 0.7 ml medium without antibiotics. To generate the transfection mix, 2 μg herring-testis DNA (Sigma-Aldrich, D6898-1G) was combined with 0.15 ml Opti-MEM (Life Technologies, 51985-034). A 6 μl volume of Lipofectamine 2000 (Life Technologies, 11668) was separately mixed with 0.15 ml Opti-MEM. Both mixtures were incubated at room temperature for 5 min, combined and incubated at room temperature for 15 min before being added to the cell dish. The samples were harvested for RNA extraction 6 h post stimulation.

To prepare complementary DNA, approximately 4 × 10^5^ cells were harvested and resuspended in 0.8 ml TRI-Reagent (Sigma-Aldrich, T9424-25ML). This mixture was frozen at −80 °C until use. For RNA extraction, 0.2 ml chloroform was added to the samples after thawing, which were then transferred to Phase-lock gel tubes, heavy (VWR, 733-2478). Following extensive mixing, the samples were incubated at room temperature for 2 min before centrifugation for 5 min at 12,000*g* and 4 °C. The aqueous phase was removed and mixed with an equal volume of ethanol. After mixing, the samples were loaded onto RNeasy columns (Qiagen), washed once with buffer RW1 and twice with buffer RPE. The samples were eluted using 35 μl nuclease-free water. Approximately 1 μg RNA of each sample was used to generate cDNA with a High-capacity cDNA reverse transcription kit (Applied Biosystems, 4368813) according to the manufacturer’s instructions. The cDNA samples were diluted 1:10 in nuclease-free water; 4 μl of each cDNA reaction was used for quantitative PCR with the SYBR Green PCR master mix (Applied Biosystems, 4344463) carried out on an StepOnePlus real-time PCR system (Applied Biosystems, 4376600). Amplification conditions were: 95 °C for 10 min, followed by 40 cycles of 95 °C for 15 s and 60 °C for 1 min. The following oligonucleotides were used: 5′-AATCCCATCACCATCTTCCA-3′ and 5′-TGGACTCCACGACGTACTCA-3′ for GAPDH^92^; 5′-TGCCCACATCAAGGAGTATTT-3′ and 5′-CTTTCGGGTGACAAAGACG-3′ for CCL5 (ref. ^93^; Supplementary Table 2).

### FRET measurement by flow cytometry

HEK293FT cells were seeded in 10 cm dishes to achieve 80% confluency on day 1. The cells were transfected with the cGAMP reporter using Lipofectamine 2000 (Invitrogen) as per the manufacturer’s instructions and treated 24 h later with medium or diABZI (1 μM) for 1.5 h. The cells were then washed with PBS, trypsinized and spun down, and the pellet was resuspended in PBS. MDA-MB-231 cells stably expressing the cGAMP reporter were treated with medium or diABZI (1 μM) for 18 h before being washed with PBS, trypsinized and spun down; the pellet was resuspended in PBS.

Live-cell flow cytometry FRET measurements were performed on a BD FACSymphony S6 cell sorter (BD Biosciences) piloted by BD FACSDiva 9.5 (BD Biosciences). mTurq2 was excited using a Violet 405 nm diode laser with a power of 100 mW. mTurq2 was detected with the 405–474/25 channel at 510 V. YPet-FRET was detected with the 405–530/30 channel at 400 V. Data were analysed using the FlowJo software. Briefly, after gating for single cells based on FSC-H and FSC-A, we gated the mTurq2^+^ population based on the parental cells that did not express the reporter.

All FACS data in this manuscript were plotted with FlowJo (BD Biosciences) as pseudocolour density plots.

### cGAS stimulation with DNA in cells

For experiments using plasmid DNA in cells as a stimulus for cGAS activation, cells were seeded on a μCLEAR 96-well plate (Greiner Bio-one, 655090) in MCF10A culture medium without phenol red. The cells were seeded to reach 80% confluency on the the day of imaging. pBluescript SK(+) plasmid DNA was suspended in 25 μl MCF10A culture medium without serum or antibiotics and mixed with a second tube containing 0.5 μl Lipofectamine 2000 (Invitrogen) in 25 μl MCF10A culture medium without serum or antibiotics. The mixture was incubated for 20 min and added to a well containing cells in 100 μl MCF10A culture medium. Unless specified otherwise, the cells were imaged 8 h post transfection.

For analysis of cGAMP cell-to-cell transfer (Fig. 2g–i), cells were seeded to be a 100% confluent monolayer on the day of imaging. The cells were transfected, as described in the previous paragraph, with 400 ng pBluescript SK(+) per well but with a fourfold reduction in the Lipofectamine 2000 final concentration in the mix to limit the number of cell transfection events per well. The cells in Fig. 2i were treated with carbenoxolone at the indicated concentration immediately after plasmid transfection. Time-lapse image acquisition was started immediately after DNA transfection.

For transfection of model substrates (Fig. 6 and Extended Data Fig. 8), 8 pmol dsDNA, DNA:RNA hybrid or R-loop model substrate was suspended in 25 μl MCF10A culture medium without serum or antibiotics, mixed with a second tube containing 0.5 μl Lipofectamine 2000 (Invitrogen) in 25 μl MCF10A culture medium without serum or antibiotics, incubated for 40 min and then added to the well containing the cells in 100 μl of MCF10A culture medium. The cells were imaged 24 h post transfection.

### Preparation and use of MVA

The MVA particles were prepared as previously described^94^. For infections, MVA particles were diluted in MCF10A culture medium without phenol red to infect MCF10A cells stably expressing the cGAMP reporter at a multiplicity of infection of 1, 5 or 10. Live cells were imaged 8 h post infection.

### Live-cell imaging

For live-cell experiments, MCF10A cells stably expressing the cGAMP FRET reporter were seeded on a μCLEAR 96-well plate or μ-Slide 8 well high ibiTreat #1.5 polymer tissue culture-treated chamber slide (IBIDI) in MCF10A culture medium without phenol red reach approximately 80% confluency on the day of imaging. For radiation experiments, unless specified otherwise, cells were exposed to 15 Gy of X-ray radiation and allowed to recover for 48 h before imaging. For SUM149 cells, the medium was changed to DMEM/F12 without phenol red plus 5% FBS immediately before imaging. For both cell lines, nuclei were stained with 200 nM SiR-DNA (Spirochrome) 2–6 h before imaging.

For FRET experiments in HeLa cells, 100,000 cGAS-knockout HeLa cells were transiently co-transfected with the cGAMP reporter (or cGAMP-blind mutant) with or without 10 μg cGAMP using the Neon transfection system (Invitrogen; 10 μl tips; programme, 1,005 V, two pulses, 35 ms). Following transfection, the cells were immediately seeded on a μ-Slide 8 well high ibiTreat #1.5 polymer tissue culture-treated chamber slide (IBIDI) containing 300 μl DMEM without phenol red plus 5% FBS. The cells were allowed to recover for 48 h before imaging.

FRET images were acquired on a Zeiss Axio Observer Z1 piloted by SlideBook (3i) and equipped with a Yokogawa CSU-X1 spinning-disk unit, as well as with 445 nm 75 mW, 488 nm 50 mW, 514 nm 40 mW, 561 nm 50 mW and 640 nm laser illumination sources. Emission filters (Semrock) were as follows: 440/521/607/700, ChromaZET440/514/561/640, 482(35), 542(27), 617(73), 525(30). To maximize the number of cells in each field of view, a Zeiss Plan-Apochromat ×20/0.8 objective was used for all experiments. Temperature and CO_2_ were maintained constant at 37 °C and 5% CO_2_ using an Okolabs Bold Line T-Unit1 and Okolabs Bold line CO_2_/O_2_ unit.

To assess bleed-through between the CFP and YFP FRET channels, cGAS-knockout HeLa cells were transfected with an mTurquoise2–mSTING–mTurquoise2 or H2B–Citrine construct. Using the CFP excitation laser, 39.7 ± 3.1% (mean ± s.d.) of the mTurquoise2–mSTING–mTurquoise2 fluorescence could be detected with the YFP emission filter, and 0% of the H2B–citrine signal could be detected with the CFP emission filter. When using the YFP excitation laser, no mTurquoise2–mSTING–mTurquoise2 fluorescence could be detected with either the CFP or YFP emission filters, and no H2B–citrine signal could be detected with the CFP emission filter.

Snapshot images for both MCF10A and SUM149PT were acquired on a Hamamatsu Flash4.0 V3 camera with full laser power and 2 × 2 camera binning. Exposure was set at the same 600-ms value for all three FRET channels (CFP, YFP and CFP-excited YFP emission) and at 100 ms for SiR-DNA staining. To capture rare FRET activation events following DNA-damage induction, at least 50–80 fields of view were acquired per well and multiple technical replicates were combined per experimental condition.

For time-lapse FRET, mScarlet3–cGAS and GFP–IRF3 imaging, the camera was switched to a Photometrics Prime95b with Gain 3 and laser power reduced to 20% to limit photodamage. Images were acquired every 15 or 20 min for FRET experiments and every 5 min for GFP–IRF3. For the cumulative IR FRET activation plot in Fig. 3h, images were acquired every 120 min on a Flash4 camera with full laser power. For time-lapse FRET DNA-damage experiments, the acquisition was started either immediately following X-ray irradiation or 3 h after the addition of 0.5 μM reversine.

When drugs were added during a time-lapse acquisition, they were diluted to 10× concentration in 10 μl imaging media and then added drop-wise to the well containing cells in 100 μl imaging medium. Care was taken not to touch the plate with the pipette tip and not to open the imaging chamber for more than a minute to limit temperature and CO_2_ shifts. For stimulation with DNA during time-lapse imaging, 50 μl transfection mix (as described in the ‘cGAS stimulation with DNA in cells’ section) in MCF10A imaging medium without serum or antibiotics was added drop-wise to the well, increasing the total well volume to 150 μl.

### Fixed-cell FRET imaging

Cells seeded in a μCLEAR 96-well plate were treated with 200 nM Sir-DNA for 2–4 h, washed once in PBS and then fixed in 4% paraformaldehyde for 10 min. The cells were washed 3× with PBS then stored at 4 °C, protected from light. The cells were imaged as described earlier for snapshots of live cells.

### Immunofluorescence

Cells seeded in a μCLEAR 96-well plate were washed with PBS, fixed in 4% paraformaldehyde for 10 min, washed 3× in PBS and then stored at 4 °C for further processing. The cells were permeabilized in PBS + 0.1% Triton X-100 for 10 min, washed in PBS and blocked in 3% BSA for 1 h. Next, the cells were incubated in primary antibody for 1 h, washed 3× in PBS + 0.1% tween (PBST), followed by incubation for a further 1 h with secondary antibody. The cells were then washed 3× in PBST, incubated in 10 μg ml^−1^ Hoechst 33342 (Thermo) for 5 min and stored in PBST at 4 °C.

Immunofluorescence images were acquired on an ImageXpress imaging system (Molecular Devices) equipped with 377/54, 475/34, 531/40 and 631/28 excitation filters, and 447/60, 536/40, 593/40, 624/40 and 692/40 emission filters. Images were acquired with a ×20 objective in wide-field mode.

### Image processing and data analysis

Segmentation, tracking and pixel value quantification were performed using custom Python scripts^95^. Manual annotations for time-lapses were done with Napari using custom Python scripts. Python scripts based on Pandas and Numpy were used for data processing, and matplotlib and seaborn to generate graphs. Alternatively, processed data were exported as a table to be imported and plotted in GraphPad Prism. All code used in this work is available in *Zenodo* (https://doi.org/10.5281/zenodo.19789597)^96^.

For both live and fixed cells, nuclear signal (SiR-DNA, H2B–RFP or Hoechst 33342) was used to segment nuclei using StarDist^97^. A 3-pixel-wide ring was generated 3 pixels away and around the periphery of every nucleus to obtain a ‘cytosolic’ region. FRET ratios were calculated by dividing measured pixel values in the YPet FRET channel (CFP excitation laser, YFP emission filter) by the measured pixel values in the mTurquoise2 donor channel (CFP excitation laser, CFP emission filter). Care was put into determining which approach gave the most robust to noise and reproducible measurements across a range of conditions and it was ultimately decided to measure median values of channels of interest in the cytosolic region. All FRET data in this work show cytoplasmic FRET ratio values.

Despite being derived from a clone, slight variability in expression levels is apparent in cells stably expressing the cGAMP FRET reporter and FRET measurements do not seem to be fully accurate below a certain expression level. To account for that, the YFP fluorescence channel (YFP excitation laser, YFP emission filter) was used to assess expression level and cells in which reporter expression was deemed too low were fully excluded from any further analysis. In this work, a threshold of 1,500 a.u. for median YFP cytosolic intensity was empirically determined to be the most robust and reproducible across different treatment conditions (Extended Data Fig. 4b). This threshold needs to be optimized according to cell line, expression level and imaging conditions. Note that because baseline FRET can vary between experiments due to stochastic differences in cellular parameters or imaging conditions, exact *y*-axis values are not necessarily comparable between independent experiments.

For all FRET experiments showing heterogeneous and partial response to DNA damage, a threshold of activation was defined in each experiment as the 99th (for MCF10A cells) or 98th (for SUM149 cells) percentile of all measured FRET values in the control condition. Any cell for which the measured FRET ratio was above this threshold was classified as active and plotted as a red dot; cells below this threshold were plotted in grey. The percentage of cells in the population above the threshold (and therefore classified as ‘active’) is indicated for each condition in a red box at the top of the graph along with the total cell number (active or inactive) in the given condition.

Treatments such as radiation and high doses of reversine severely impact cell growth compared with the untreated control condition. To display comparable cell numbers across all conditions, and when applicable, cells were randomly selected using the unbiased ‘sample’ function from Python pandas before plotting. The percentage of active cells was calculated from the total population (before sampling).

For Extended Data Fig. 4e, autofluorescence from doxorubicin affected the baseline of the measured FRET ratio. To account for that non-cGAMP-related shift, the FRET ratio of each cell was divided by the average FRET measurement of MCF10A cells treated with the same doxorubicine concentration but stably expressing the mutant cGAMP-blind reporter instead of the wild type. Extended Data Fig. 4e is the only graph that was normalized to the average cGAMP-blind reporter FRET values.

For STING signal analysis in immunofluorescence, nuclei stained with Hoechst were first segmented with StarDist. The cGAMP reporter was then segmented with CellPose to obtain a full cell mask. Mean and maximum values of STING antibody signal in the full cell mask as well as the total integrated density in the full cell mask were measured. The STING accumulation index was calculated as described previously by dividing the maximum pixel intensity signal in the full cell by the mean signal^33^.

For time-lapse experiments, detected nuclei from each frame were linked in time using btrack^98^. To account for cells entering or exiting the field due to cell motility, only traces that lasted more than 37.5 h were considered for analysis in Fig. 3g. For time-lapse experiments, individual traces were smoothed with a rolling median algorithm using a centred 1-h window.

To automatically detect immunofluorescent cGAS^+^ micronuclei, the cGAS immunofluorescence staining channel was segmented using StarDist. Detected particles were filtered based on size and fluorescence to exclude main nuclei and only keep cGAS^+^ micronuclei. Distances from every detected cGAS^+^ particle to all neighbouring main nuclei (defined by the Hoechst staining) were computed and used to match every detected cGAS^+^ particle to its closest main nucleus. For Figs. 4j,k and 5c–e, mScarlet3–cGAS^+^ bridges and micronuclei were manually annotated.

For Fig. 2g–i, extension of the FRET^+^ area was manually segmented in each frame using Fiji. Figure 2i shows maximal extent of the FRET^+^ area, monitored 18 h after DNA transfection.

Mosaics (Extended Data Fig. 4a) were generated using the Slidebook software (3i). Representative microscopy images were assembled using Fiji.

### Analysis of FRET^+^ neighbour cell number and minimal distance to FRET^+^ cell

MCF10A cells stably expressing the cGAMP FRET reporter were seeded on a μCLEAR 96-well plate in MCF10A culture medium without phenol red to reach 80% confluency on the day of imaging. Live cells were imaged as described in the previous section. For Fig. 3j,k, acquired images were stitched into a mosaic (approximately 4 mm × 4 mm) before analysis. Active FRET^+^ cells were identified as described in the previous section using the untreated condition to define a 99th-percentile threshold, with any cell with FRET ratio values above that threshold to be classified as FRET^+^ in both the untreated and treated conditions.

Distances from each FRET^+^ cell to every other FRET^+^ cell in the stitched mosaic were computed and both the distance to the closest other FRET^+^ cell and the number of FRET^+^ cells within a 200 μm radius were calculated for each FRET^+^ cell. This radius of 200 μm was chosen empirically to match the size of observed clusters of FRET^+^ cells after IR (for example, Fig. 3j).

To generate the simulated ‘random’ condition in Fig. 3k and Extended Data Figs. 5a,b, 6h, centroid coordinates of every cell were extracted, regardless of their FRET status. A fraction of these centroid coordinates (equal to the fraction of FRET^+^ cells in the actual experimental dataset) were randomly assigned to be simulated FRET^+^, and both the distance to the closest other simulated FRET^+^ cell and the number of simulated FRET^+^ cells within a 200 μm radius were calculated for each cell randomly classified as simulated FRET^+^. This process of randomly assigning simulated FRET^+^ status to centroid coordinates from the actual dataset was repeated for 100 iterations and the results were consolidated into a single ‘Simulated’ dataset. All clustering analysis was performed using custom Python scripts.

### Analysis of MN^+^ cells in FRET^+^ clusters

FRET^+^ cells were identified as described in the previous section using the untreated condition to define a 99th-percentile threshold, with any cell with FRET ratio values above that threshold to be classified as FRET^+^. To define local clusters of FRET^+^ cells (hereafter referred as ‘FRET^+^ cluster’) in Extended Data Fig. 6b,j, a single pass clustering with a 200 μm threshold was applied to all FRET^+^ cells. Briefly, for every FRET^+^ cell, if the distance from its centroid to the centroid of an already-found cluster was <200 μm, the FRET^+^ cell was classified as being part of that cluster. If no already-found cluster had its centroid within <200 μm of the FRET^+^ cell, a new empty cluster was created and the FRET^+^ cell added to it.

Cells with a cGAS^+^ micronucleus (hereafter referred to as ‘MN^+^’ cells) were identified by correlative cGAS immunofluorescence (‘Correlative live FRET imaging and immunofluorescence’ section). The presence of a MN^+^ cell was assessed for each FRET^+^ cluster.

To generate the simulated random MN^+^ cell dataset in Extended Data Fig. 6c,k, *n* cells from the full experimental dataset were randomly classified as ‘simulated MN^+^ cells’, regardless of their FRET^+^ or MN^+^ status, with *n* equal to the real count of MN^+^ cell in the dataset. Presence of ‘simulated MN^+^ cells’ was assessed for each of the previously determined FRET^+^ clusters. This process of randomly classifying cells as ‘simulated MN^+^ cells’ and testing presence in the previously determined clusters was repeated for 1,000 iterations. The proportion of FRET^+^ clusters with ≥1 ‘real’ MN^+^ cell was then compared with the proportion of FRET^+^ clusters that were found to have ≥1 randomly assigned ‘simulated’ MN^+^ cell over 1,000 iterations using the two-tailed Fisher’s exact test.

### Time-lapse annotation of cell-cycle states, FRET^+^ clusters and cGAS^+^ nuclear structures

Primary cGAS-activated cells and their time of activation were manually determined in a time lapse by identifying either isolated FRET activation events or by identifying the first cell to activate in a local cluster of FRET^+^ cells. Parents of primary cGAS-activated cells were then determined and prometaphase, G1 onset, as well as onset/disappearance of cGAS^+^ bridges and cGAS^+^ micronuclei were manually determined for each cell in the parent lineage. The parent lineage of manually identified primary cGAS-activated cells and presence of cGAS^+^ bridges or cGAS^+^ micronuclei in the lineage was plotted using custom Python scripts.

### Correlative live FRET imaging and immunofluorescence

Following acquisition of live-cell FRET images, the cells were immediately washed in PBS and fixed in 4% paraformaldehyde for 10 min. Immunofluorescence incubations were performed as described earlier. To limit interaction with the CFP and YFP channel, only a single primary antibody was incubated with the cells and further detected by a red fluorescence Cy3 secondary antibody.

For all wells in which FRET images were previously acquired, a tiled mosaic encompassing the full well was generated and the Hoechst 33342 and Cy3 immunofluorescence staining acquired on either a wide-field ImageXpress (Molecular Devices) microscope with a ×20 objective or on a spinning-disk confocal Opera Phenix (Perkin-Elmer) microscope with a ×40 water-immersion objective.

To identify cells from the live-imaging experiment in the follow-up immunofluorescence acquisition, individual images from the live FRET experiment were resized to account for differences in pixel size and magnification between the two imaging set-ups. Live-cell images and the full-well immunofluorescence mosaic montage were segmented using StarDist to generate binary images, which were further processed to obtain their Fourier transform. For each field from the live imaging, the associated Fourier transform conjugate was multiplied with the mosaic image Fourier transform and then converted back with an inverse Fourier transform to compute the location of the FRET live-imaging field in the follow-up immunofluorescence full-well mosaic. Segmented objects in both live-imaging and IF datasets were filtered by size to exclude debris, and *x*–*y* coordinates of every cell from either the live-imaging or immunofluorescence images were merged into a common coordinate system adjusted for magnification and pixel-size differences. Distances between every cell from the FRET live-cell experiment to every cell from the immunofluorescence acquisition were computed into a cost matrix, which was resolved using the SciPy linear sum assignment algorithm to identify every cell from the live-imaging experiment in their immunofluorescence counterpart. Small images centred on the nuclei (SiR-DNA versus Hoechst) of identified corresponding cells were generated to assess matching quality.

Image analysis of the immunofluorescence data was performed on each individual tile instead of the reconstituted mosaic montage to prevent potential stitching artefacts from affecting the pixel values. Cells from the individual immunofluorescence tiles were matched to the mosaic montage and the corresponding live cells as described in the previous paragraph.

### Statistics and reproducibility

To capture rare FRET activation events following DNA-damage induction, at least 50–80 fields of view were acquired per well, and multiple technical replicates were combined per experimental condition. Each experiment was independently replicated at least two times (most experiments were performed three or more times); all replication attempts were successful. Where possible, at least 2,000 cells were analysed per treatment group in each biological replicate, except in conditions in which treatments severely affected viability, where a minimum of 100–500 cells were analysed per experiment. No statistical method was used to pre-determine sample size.

We used a two-tailed Fisher’s exact test in Extended Data Fig. 6c,k to compare the observed frequency of FRET^+^ clusters with at least one micronucleated cell versus their expected theoretical frequency if micronucleated cells were randomly distributed in the cell population (‘Analysis of MN^+^ cells in FRET^+^ clusters’ section). The reported *P* values are for a representative experiment of two independent repeats. Note that this statistical test was performed internally for each replicate (and not comparing the means of multiple independent replicates in one condition with the means of independent replicates in another condition like for a *t-*test).

Despite being derived from a clone, slight variability in expression levels is apparent in cells stably expressing the cGAMP reporter and FRET measurements do not seem to be fully accurate below a certain expression level. To account for that, the YFP fluorescence channel (YFP excitation laser, YFP emission filter) was used to assess expression levels. Cells in which reporter expression was deemed too low were excluded from further analysis. In this study, a threshold of 1,500 a.u. for median YFP cytosolic intensity was empirically determined to be the most robust and reproducible across different treatment conditions. Details are provided in the ‘Image processing and data analysis’ section.

Given that all our assays involved cell lines split into various wells at the time of plate seeding, resulting in highly homogeneous biological samples across the tested conditions, the experiments were not randomized.

The investigators were not blinded to allocation during experiments and outcome assessment. Blinding was not deemed relevant to our experimental set-up as almost all experiments were analysed by unbiased automated image analysis scripts.

### Protocol availability

A detailed step-by-step guide, describing cell-line generation and use of cGAMP reporter-expressing cell lines has been deposited to *protocols.io*^99^.

### Reporting summary

Further information on research design is available in the Nature Portfolio Reporting Summary linked to this article.

## Online content

Any methods, additional references, Nature Portfolio reporting summaries, source data, extended data, supplementary information, acknowledgements, peer review information; details of author contributions and competing interests; and statements of data and code availability are available at https://doi.org/10.1038/s41556-026-02037-0.

## Supplementary information

Reporting Summary

Supplementary Tables Table 1. DNA sequences of the cGAMP reporter constructs. Table 2. Oligonucleotides used in this study. Table 3. Light microscopy reporting table.

Supplementary Video 1 Time-lapse microscopy of MCF10A cells stably expressing the cGAMP reporter treated with DNA transfection, DMXAA (10 µg ml^−1^) or diABZI (1 µM; *n* = 3 independent experiments).

Supplementary Video 2 Time-lapse microscopy of MCF10A cells stably expressing the cGAMP reporter DNA transfected (1.33 µg ml^−1^) with limiting transfection reagent (*n* = 3 independent experiments).

Supplementary Video 3 Time-lapse microscopy of MCF10A cells stably expressing the cGAMP reporter activating cGAS after 7 Gy irradiation (*n* = 3 independent experiments).

Supplementary Video 4 Time-lapse microscopy of mScarlet–cGAS being recruited to a micronucleus in MCF10A cells stably expressing both the cGAMP reporter and mScarlet–cGAS treated with 0.5 µM reversine (*n* = 3 independent experiments).

## Source data

Source Data for all Figures Statistical source data.

Source Data for all Figures Unprocessed western blots and gels.

## Data Availability

Uncropped images for gels, western blots and microscopy shown in the figures have been deposited in *Zenodo* and are publicly available (https://doi.org/10.5281/zenodo.19789597)^96^. Due to the size of the raw imaging data, all data reported in this paper will be shared by C.Z. on request. Any additional information required to reanalyse the data reported in this paper is available from the lead contact on request. Source data are provided with this paper.

## Code availability

All original code has been deposited in *Zenodo* (https://doi.org/10.5281/zenodo.19789597)^96^ and is publicly available.
