# Supplementary material for: A versatile cGAMP reporter reveals principles of cGAS activation by DNA damage and chromosome instability
Source: Nat Cell Biol. 2026 Aug 21;28(9):1957–71. doi: 10.1038/s41556-026-02037-0 (PMC13561860; doi:10.1038/s41556-026-02037-0)
Supplement: Supplementary file 1 [file 41556_2026_2037_MOESM1_ESM.pdf]

Corresponding author(s): Christian Zierhut, Vivianne Lebrec

Last updated by author(s): Apr 30, 2026

# Reporting Summary

Nature Portfolio wishes to improve the reproducibility of the work that we publish. This form provides structure for consistency and transparency in reporting. For further information on Nature Portfolio policies, see our Editorial Policies and the Editorial Policy Checklist.

## Statistics

For all statistical analyses, confirm that the following items are present in the figure legend, table legend, main text, or Methods section.

| n/a | Confirmed | |
|---|---|---|
| ☐ | ☒ | The exact sample size (*n*) for each experimental group/condition, given as a discrete number and unit of measurement |
| ☐ | ☒ | A statement on whether measurements were taken from distinct samples or whether the same sample was measured repeatedly |
| ☐ | ☒ | The statistical test(s) used AND whether they are one- or two-sided<br>*Only common tests should be described solely by name; describe more complex techniques in the Methods section.* |
| ☒ | ☐ | A description of all covariates tested |
| ☒ | ☐ | A description of any assumptions or corrections, such as tests of normality and adjustment for multiple comparisons |
| ☐ | ☒ | A full description of the statistical parameters including central tendency (e.g. means) or other basic estimates (e.g. regression coefficient) AND variation (e.g. standard deviation) or associated estimates of uncertainty (e.g. confidence intervals) |
| ☐ | ☒ | For null hypothesis testing, the test statistic (e.g. *F*, *t*, *r*) with confidence intervals, effect sizes, degrees of freedom and *P* value noted<br>*Give P values as exact values whenever suitable.* |
| ☒ | ☐ | For Bayesian analysis, information on the choice of priors and Markov chain Monte Carlo settings |
| ☒ | ☐ | For hierarchical and complex designs, identification of the appropriate level for tests and full reporting of outcomes |
| ☒ | ☐ | Estimates of effect sizes (e.g. Cohen's *d*, Pearson's *r*), indicating how they were calculated |

*Our web collection on statistics for biologists contains articles on many of the points above.*

## Software and code

Policy information about availability of computer code

| | |
|---|---|
| Data collection | Flow cytometry data was collected using BD FACSDiva 9.5 (BD Biosciences). Microscopy images were acquired with Slidebook (3i, version 2022 to 2026) or MetaXpress 5 and 6 (Molecular Devices). |
| Data analysis | Flow cytometry was analysed using FlowJo 10<br>Image analysis was performed using custom python scripts (python 3.10). Key packages are btrack (0.7.0), csbdeep (0.8.2), matplotlib (3.10.8), napari (0.6.6), numpy (2.2.6), opencv-python (4.13.0.92), pandas (2.3.3), scikit-image (0.25.2), scipy (1.15.2), seaborn (0.13.2), stardist (0.9.2), tensorflow (2.20.0), tifffile (2025.5.10).<br>Other plots and statistical analysis were performed on Graphpad Prism 10.<br><br>All original code has been deposited on Zenodo and is publicly available at [doi: 10.5281/zenodo.19789597] as of the date of publication. |

For manuscripts utilizing custom algorithms or software that are central to the research but not yet described in published literature, software must be made available to editors and reviewers. We strongly encourage code deposition in a community repository (e.g. GitHub). See the Nature Portfolio guidelines for submitting code & software for further information.

## Data

Policy information about availability of data

All manuscripts must include a data availability statement. This statement should provide the following information, where applicable:

- Accession codes, unique identifiers, or web links for publicly available datasets
- A description of any restrictions on data availability
- For clinical datasets or third party data, please ensure that the statement adheres to our policy

Uncropped images for gels, western blots and microscopy shown in the figures have been deposited on Zenodo and are publicly available as of the date of publication at [doi: 10.5281/zenodo.19789597]. Due to the size of the raw imaging data, all data reported in this paper will be shared by the lead contact upon request. Any additional information required to reanalyse the data reported in this paper is available from the lead contact upon request.

## Research involving human participants, their data, or biological material

Policy information about studies with human participants or human data. See also policy information about sex, gender (identity/presentation), and sexual orientation and race, ethnicity and racism.

| | |
|---|---|
| Reporting on sex and gender | Not applicable. |
| Reporting on race, ethnicity, or other socially relevant groupings | Not applicable. |
| Population characteristics | Not applicable. |
| Recruitment | Not applicable. |
| Ethics oversight | Not applicable. |

Note that full information on the approval of the study protocol must also be provided in the manuscript.

# Field-specific reporting

Please select the one below that is the best fit for your research. If you are not sure, read the appropriate sections before making your selection.

☒ Life sciences ☐ Behavioural & social sciences ☐ Ecological, evolutionary & environmental sciences

For a reference copy of the document with all sections, see nature.com/documents/nr-reporting-summary-flat.pdf

# Life sciences study design

All studies must disclose on these points even when the disclosure is negative.

| | |
|---|---|
| Sample size | To capture rare FRET activation events following DNA damage induction, at least 50 to 80 fields of view were acquired per well and multiple technical replicates were combined per experimental condition. Each experiment was independently replicated at least two times, and in the majority of cases three times or more. Where possible, at least 2000 cells were analysed for each treatment group in each biological replicates, except in conditions in which treatments severely affected viability. In such cases, a minimum of 100-500 cells was analysed for each experiment. |
| Data exclusions | Despite being derived from a clone, slight variability in expression levels are apparent in cells stably expressing the cGAMP FRET reporter and FRET measurements do not appear to be fully accurate below a certain expression level. To account for that, the YFP fluorescence channel (YFP excitation laser, YFP emission filter) was used to assess expression level and cells in which reporter expression was deemed too low were fully excluded from any further analysis. In this work, a threshold of 1500 (au) for median YFP cytosolic intensity was empirically determined to be the most robust and reproducible across different treatment conditions. |
| Replication | All experiments were conducted at least two independent times (and in most cases three independent times or more) with all attempts at replication being successful. |
| Randomization | Not applicable. |
| Blinding | Not applicable. |

# Reporting for specific materials, systems and methods

We require information from authors about some types of materials, experimental systems and methods used in many studies. Here, indicate whether each material, system or method listed is relevant to your study. If you are not sure if a list item applies to your research, read the appropriate section before selecting a response.

## Materials & experimental systems

| n/a | Involved in the study |
|---|---|
| ☐ | ☒ Antibodies |
| ☐ | ☒ Eukaryotic cell lines |
| ☒ | ☐ Palaeontology and archaeology |
| ☒ | ☐ Animals and other organisms |
| ☒ | ☐ Clinical data |
| ☒ | ☐ Dual use research of concern |
| ☒ | ☐ Plants |

## Methods

| n/a | Involved in the study |
|---|---|
| ☒ | ☐ ChIP-seq |
| ☐ | ☒ Flow cytometry |
| ☒ | ☐ MRI-based neuroimaging |

# Antibodies

Antibodies used

- cGAS (D1D3G) #15102 from Cell Signaling Technology (CST) (1:300 dilution for immunofluorescence; 1:1000 dilution for Western Blot)
- IRF3 (D6I4C) #11904 from CST (1:500 dilution for IF)
- phospho-IRF3 Ser396 (4D4G) #4947 from CST (1:100 for IF)
- phospho-STAT1 Tyr701 (58D6) #9197 from CST (1:300 dilution for IF)
- γH2AX Ser139 (JBW301) #05-636-25UG from Millipore (1:500 for IF)
- STING (D2P2F) #13647 from Cell Signalling Technology (1:1000 for Western Blot)
- STING (E9X7F) #90947 from Cell Signalling (1:1000 for IF)
- α-tubulin (DM1A) #T9026 from Sigma (1:5000 for Western Blot)

Validation

- cGAS (D1D3G) #15102 was validated by our lab (PMID: 31299200, Zierhut et al, Cell 2019).
- IRF3 (D6I4C) #11904 was validated by manufacturer https://www.cellsignal.com/products/primary-antibodies/irf-3-d6i4c-rabbit-monoclonal-antibody/11904?srsltid=AfmBOor0iCA4K_gW1YM7W4BtBNCUcKM1NSejvqwO5_pof-gkPKJmtubW
- phospho-IRF3 Ser396 (4D4G) #4947 was validated by manufacturer https://www.cellsignal.com/products/primary-antibodies/phospho-irf-3-ser396-4d4g-rabbit-monoclonal-antibody/4947
- phospho-STAT1 Tyr701 (58D6) #9197 was validated by manufacturer https://www.cellsignal.com/products/primary-antibodies/phospho-stat1-tyr701-58d6-rabbit-monoclonal-antibody/9167
- γH2AX Ser139 (JBW301) #05-636-25UG was validated by De Lange lab (PMID: 35027730, Mirman et al, NCB 2022)
- STING (D2P2F) #13647 was validated by our lab (PMID: 31299200, Zierhut et al, Cell 2019).
- STING (E9X7F) #90947 was validated by manufacturer https://www.cellsignal.com/products/primary-antibodies/sting-e9x7f-rabbit-monoclonal-antibody/90947?srsltid=AfmBOorPG3YJNWkKca3ZrKuLAt554qOYRGueT0IvqVTuecD15OF1OgSl
- α-tubulin (DM1A) #T9026 was validated by manufacturer https://www.sigmaaldrich.com/GB/en/product/sigma/t9026

# Eukaryotic cell lines

Policy information about cell lines and Sex and Gender in Research

Cell line source(s)

- MCF10A were a kind gift from Jörg Mansfeld (PMID:28564611).
- HEK293FT were kindly provided by Kristian Helin, ICR London, UK.
- SUM149PT were kindly provided by Chris Lord, ICR London, UK.
- MDA-MB-231 were previously used by the lab (PMID:31299200)
- Hela Flp-IN T-Rex H2B-RFP cGAS KO were previously used by the lab (PMID:31299200)
- HeLa Flp-IN T-Rex H2B-RFP cGAS KO with EGFP-cGAS rescue were previously used by the lab (PMID:31299200).

The following cell lines were generated as part of this work:
- MCF10A stably expressing cGAMP reporter
- MCF10A stably expressing cGAMP-blind mutant cGAMP reporter
- MCF10A STING KO stably expressing cGAMP reporter
- MCF10A cGAS KO stably expressing cGAMP reporter
- MCF10A stably expressing cGAMP reporter and mScarlet3-cGAS
- MCF10A stably expressing cGAMP reporter and inducible mScarlet3-NLS-cGAS R255E
- MCF10A stably expressing GFP-IRF3
- MDA-MB-231 stably expressing cGAMP reporter
- SUM149PT stably expressing cGAMP reporter
- SUM149PT stably expressing cGAMP-blind mutant cGAMP reporter
- SUM149 Revertant stably expressing cGAMP reporter

Authentication

Hela Flp-IN T-Rex H2B-RFP cGAS KO, HeLa Flp-IN T-Rex H2B-RFP cGAS KO with EGFP-cGAS rescue cells and MDA-MB-231 cells were validated using polymorphic short tandem repeat loci (PMID:31299200). Other cell lines were not authenticated.

Mycoplasma contamination

Cell lines tested negative for mycoplasma contamination.

Commonly misidentified lines
(See ICLAC register)

Not applicable.

## Plants

| | |
|---|---|
| Seed stocks | Not applicable. |
| Novel plant genotypes | Not applicable. |
| Authentication | Not applicable. |

## Flow Cytometry

### Plots

Confirm that:

☒ The axis labels state the marker and fluorochrome used (e.g. CD4-FITC).

☒ The axis scales are clearly visible. Include numbers along axes only for bottom left plot of group (a 'group' is an analysis of identical markers).

☒ All plots are contour plots with outliers or pseudocolor plots.

☒ A numerical value for number of cells or percentage (with statistics) is provided.

### Methodology

| | |
|---|---|
| Sample preparation | Cells were washed in PBS, trypsinised, washed in media, spun down and the pellet resuspended in PBS. |
| Instrument | Live cell flow cytometry FRET measurements were performed on a BD FACSymphony S6 cell sorter (BD Biosciences) |
| Software | BD FACSymphony S6 was piloted by BD FACSDiva 9.5. Data was analysed using FlowJo 10. |
| Cell population abundance | Analysis was performed on 8,000-10,000 cells gated for doublet-exclusion and reporter expression. |
| Gating strategy | After gating for single cells based on FSC-H and FSC-A, we gated the mTurq2+ population based on the parental cells that did not express the reporter. |

☒ Tick this box to confirm that a figure exemplifying the gating strategy is provided in the Supplementary Information.
