# Supplementary material for: A versatile cGAMP reporter reveals principles of cGAS activation by DNA damage and chromosome instability
Source: Nat Cell Biol. 2026 Aug 21;28(9):1957–71. doi: 10.1038/s41556-026-02037-0 (PMC13561860; doi:10.1038/s41556-026-02037-0)

Fig 1g

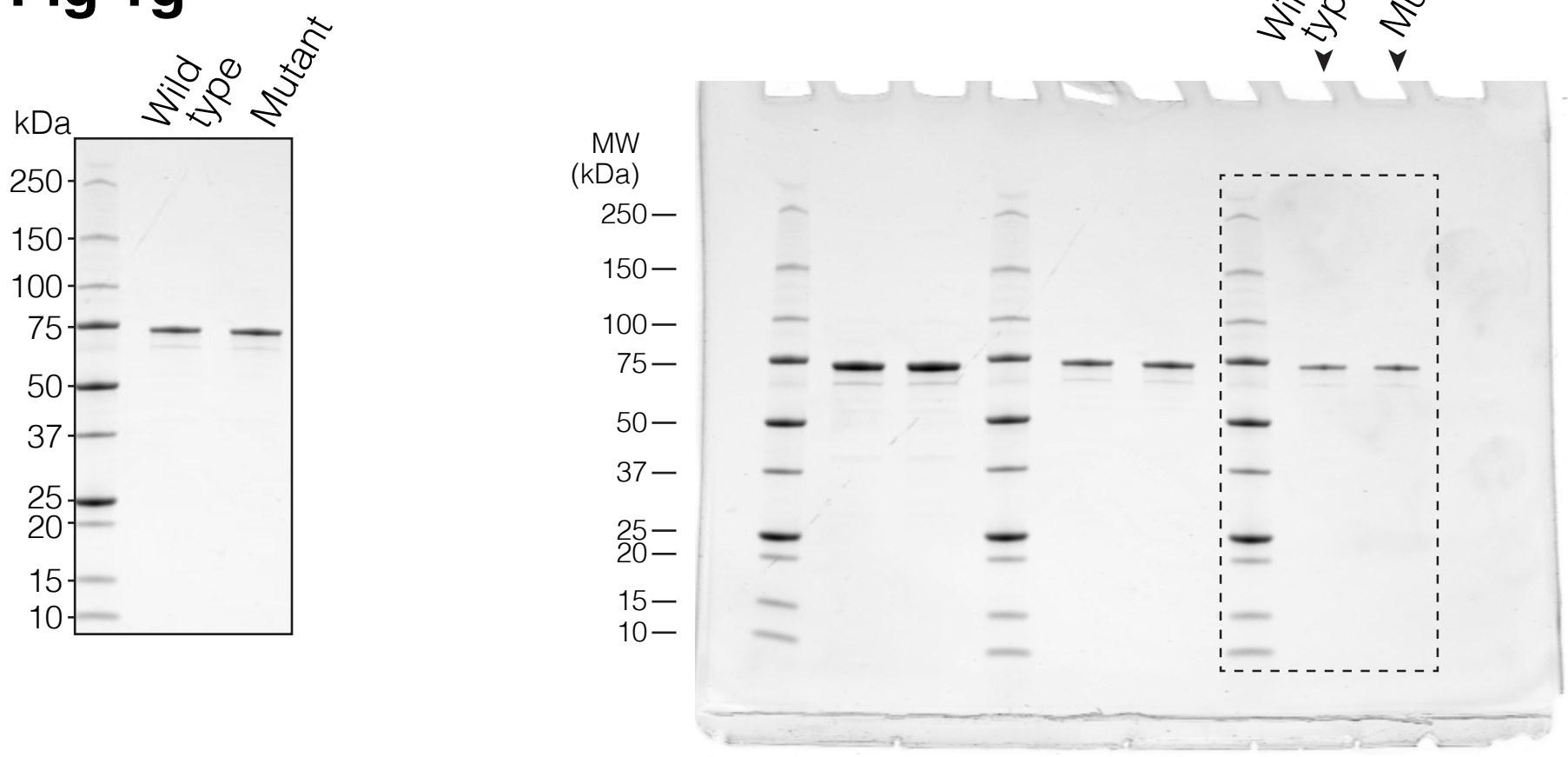

Ext. Data Fig 1e

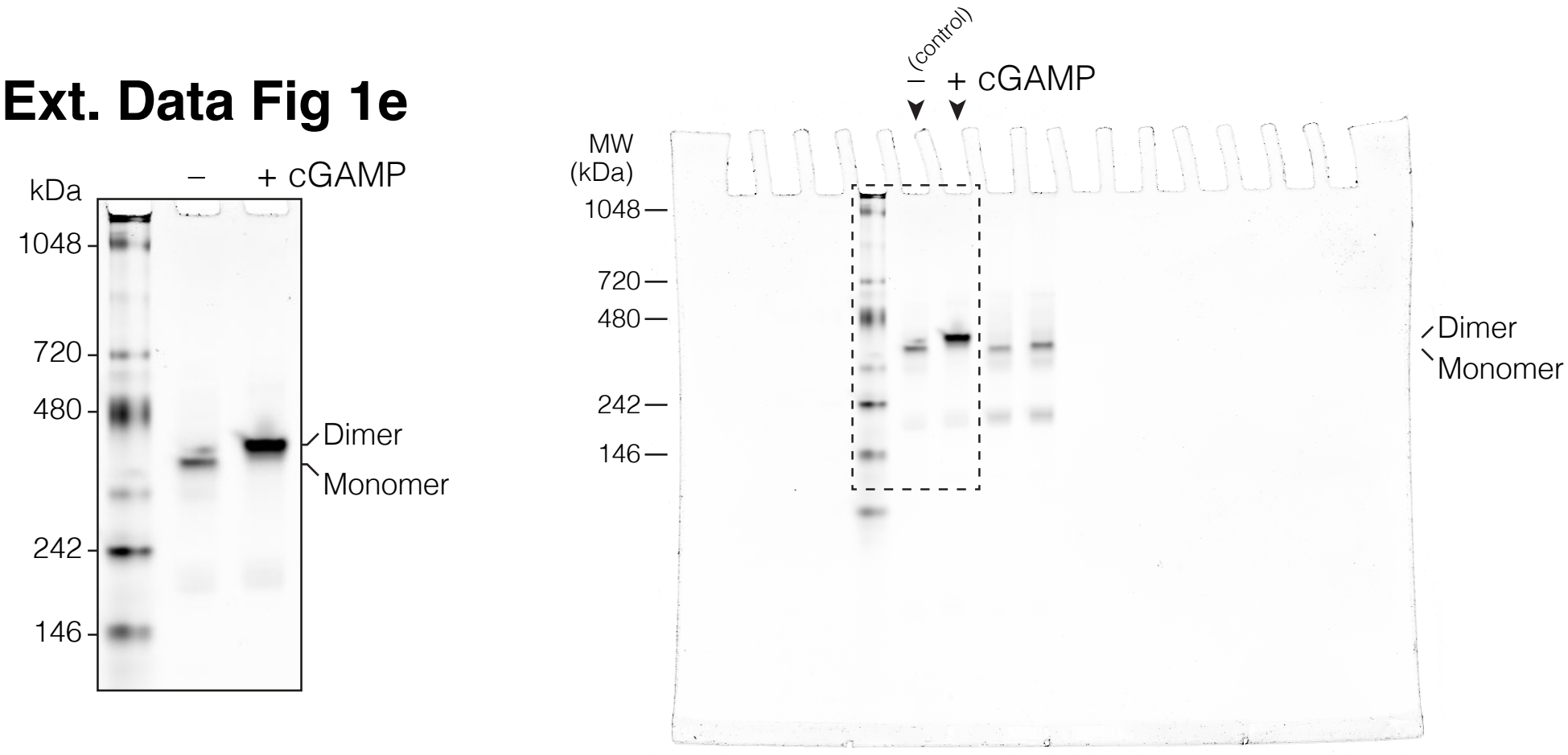

Ext. Data Fig 1f

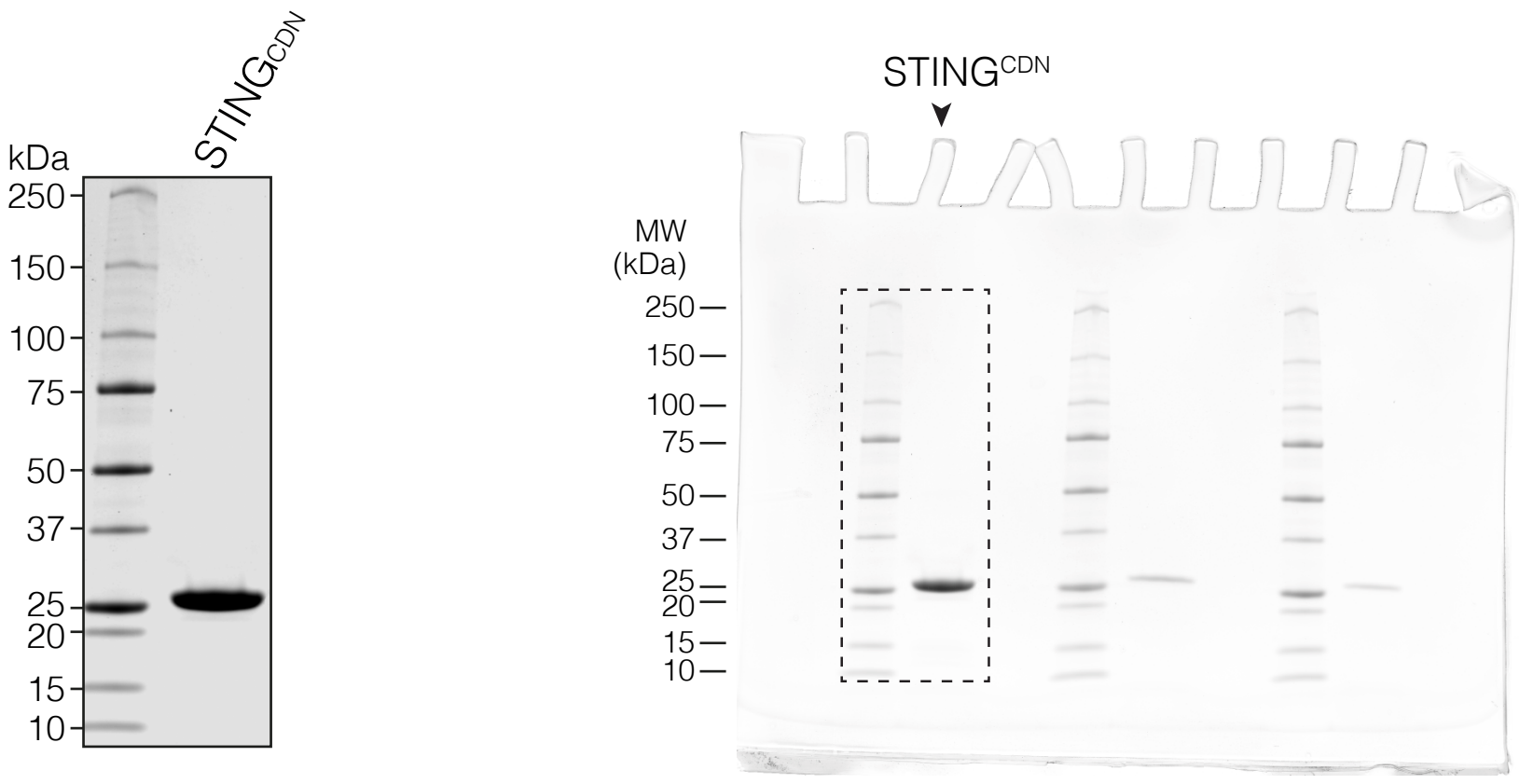

Ext. Data Fig 2c

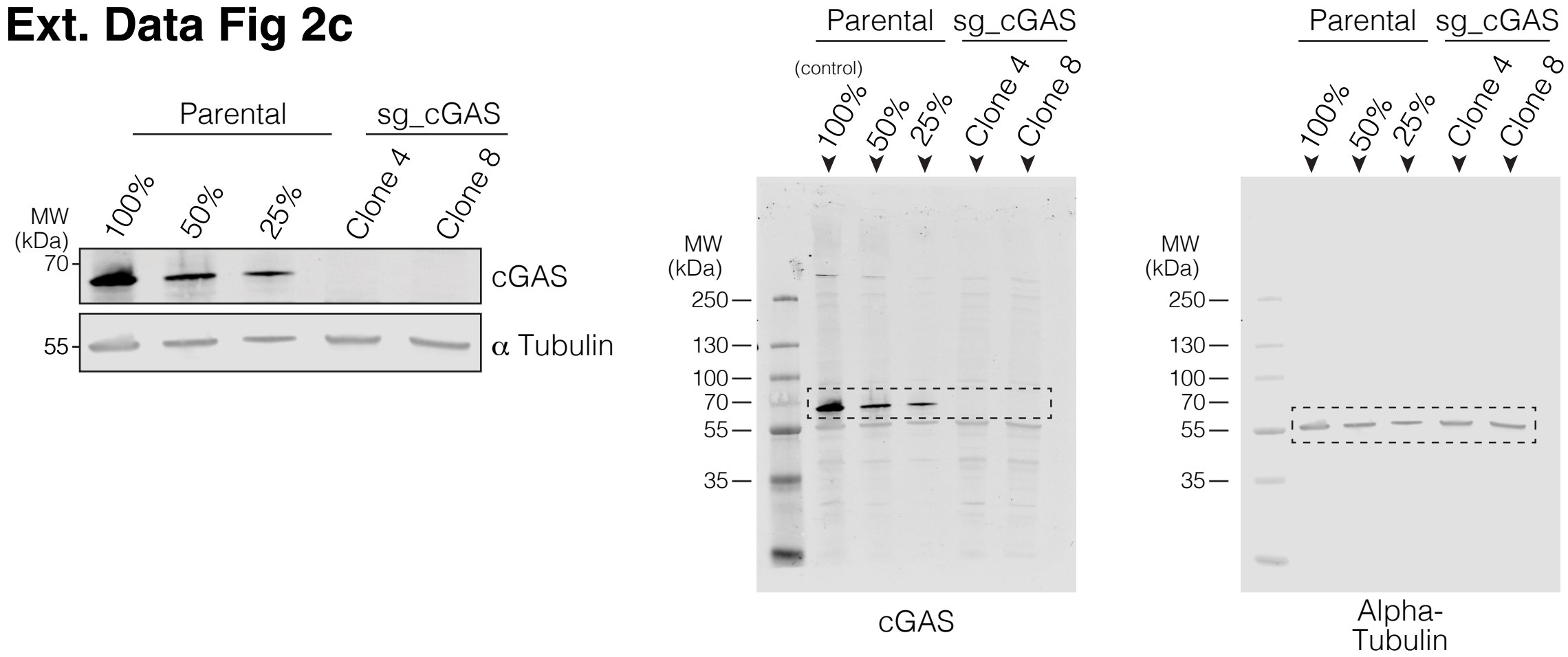

Ext. Data Fig 3i

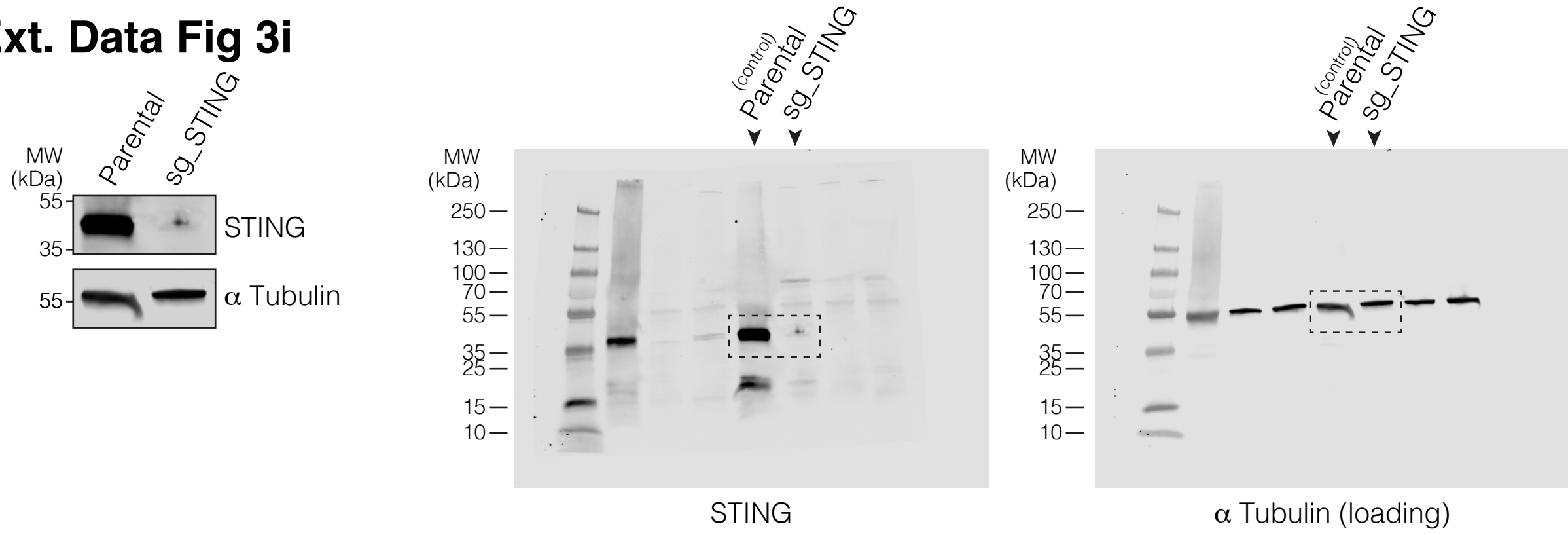

Ext. Data Fig 4h

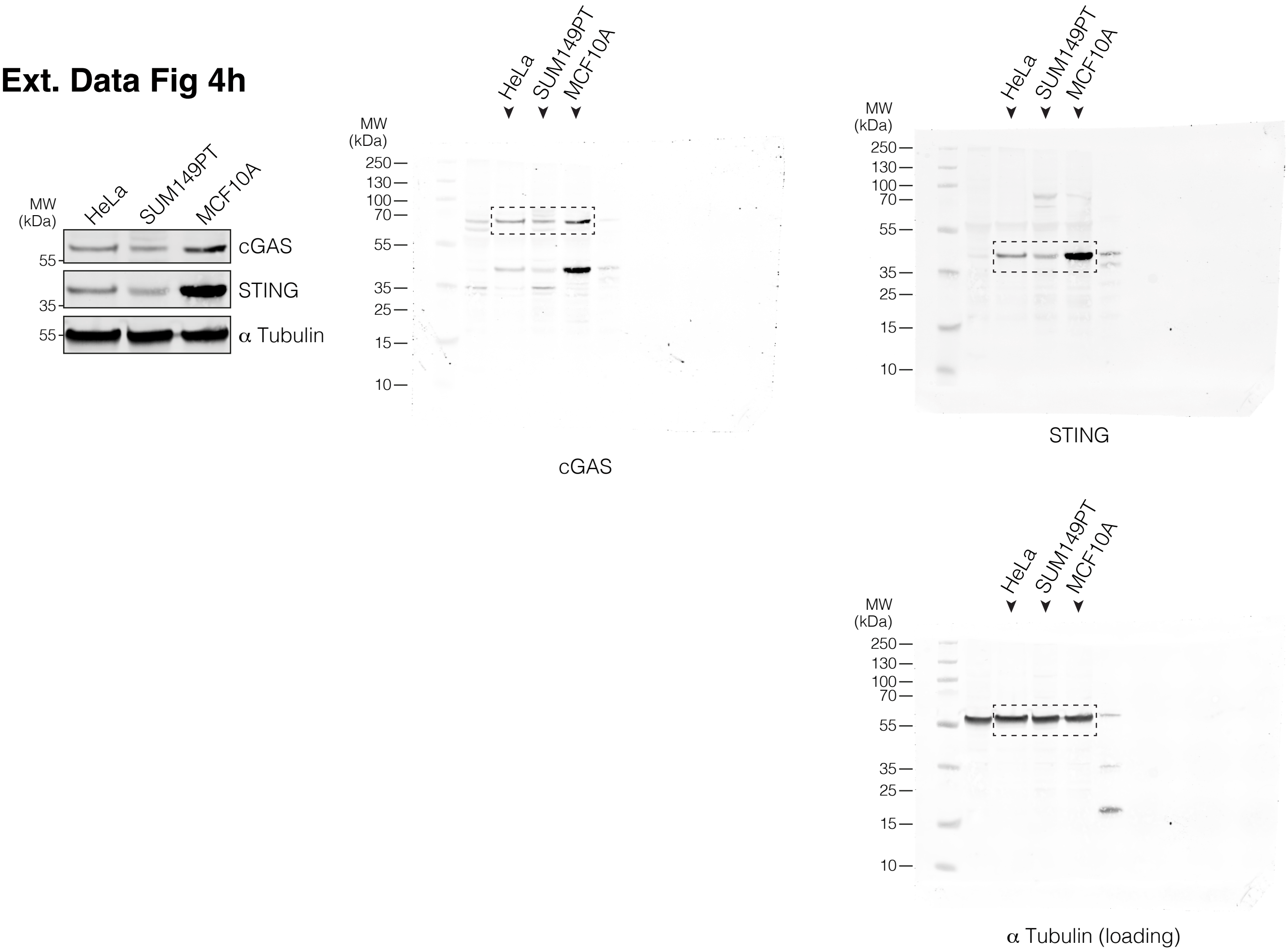

Ext. Data Fig 6l

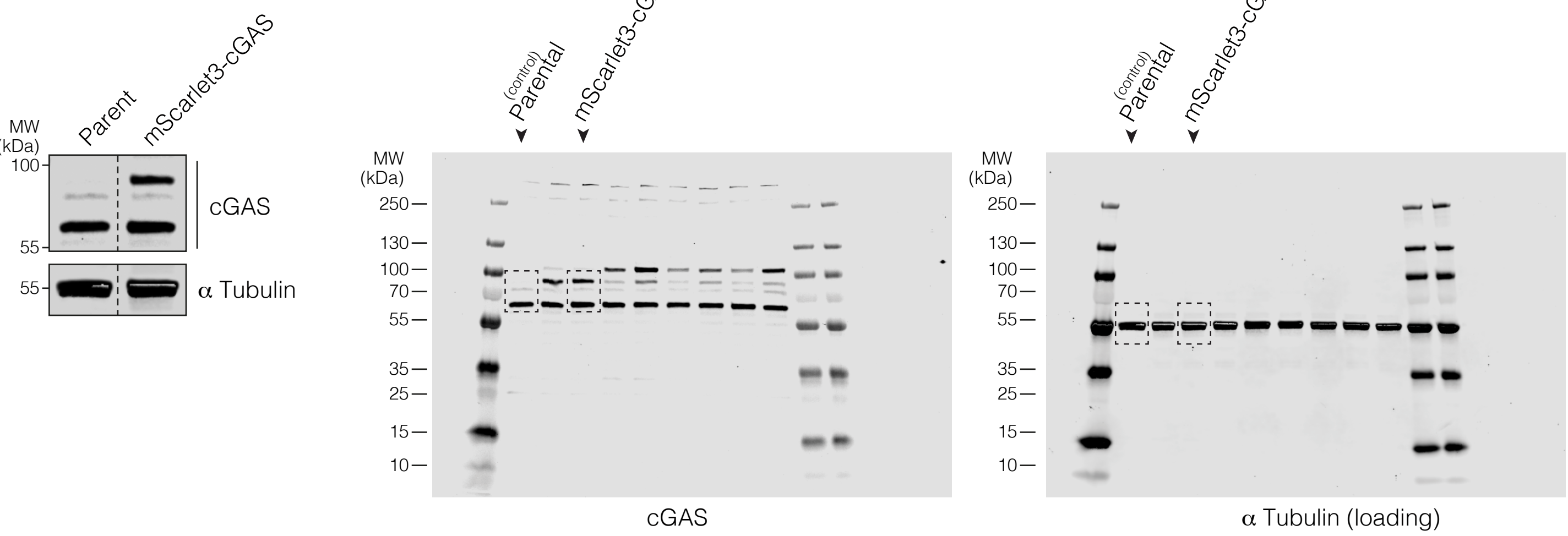

Ext. Data Fig 8b

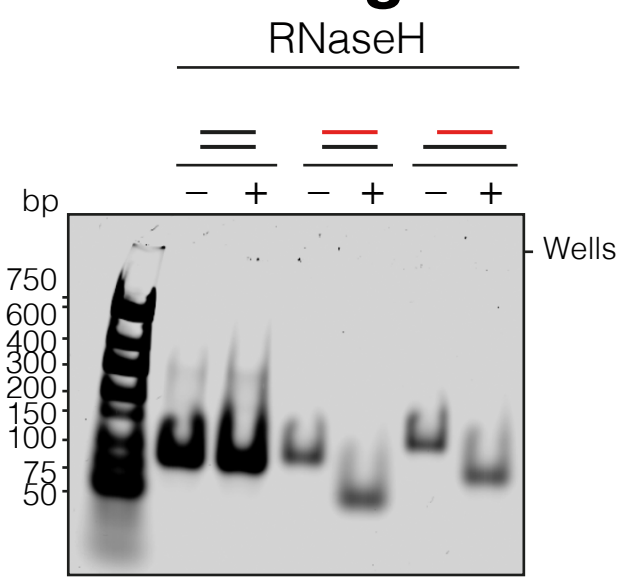

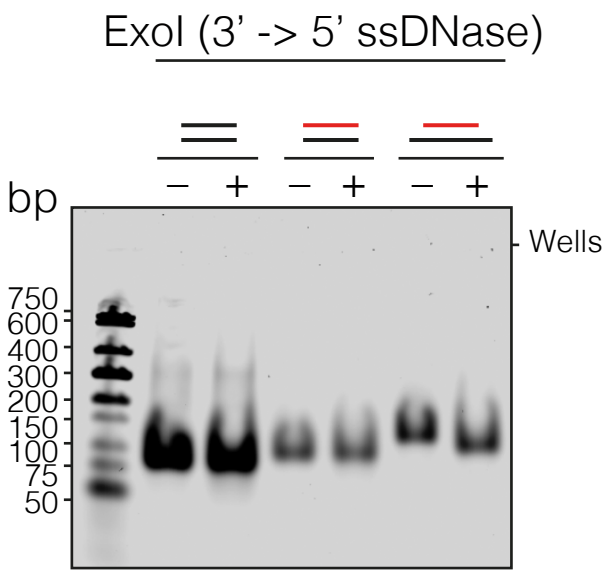

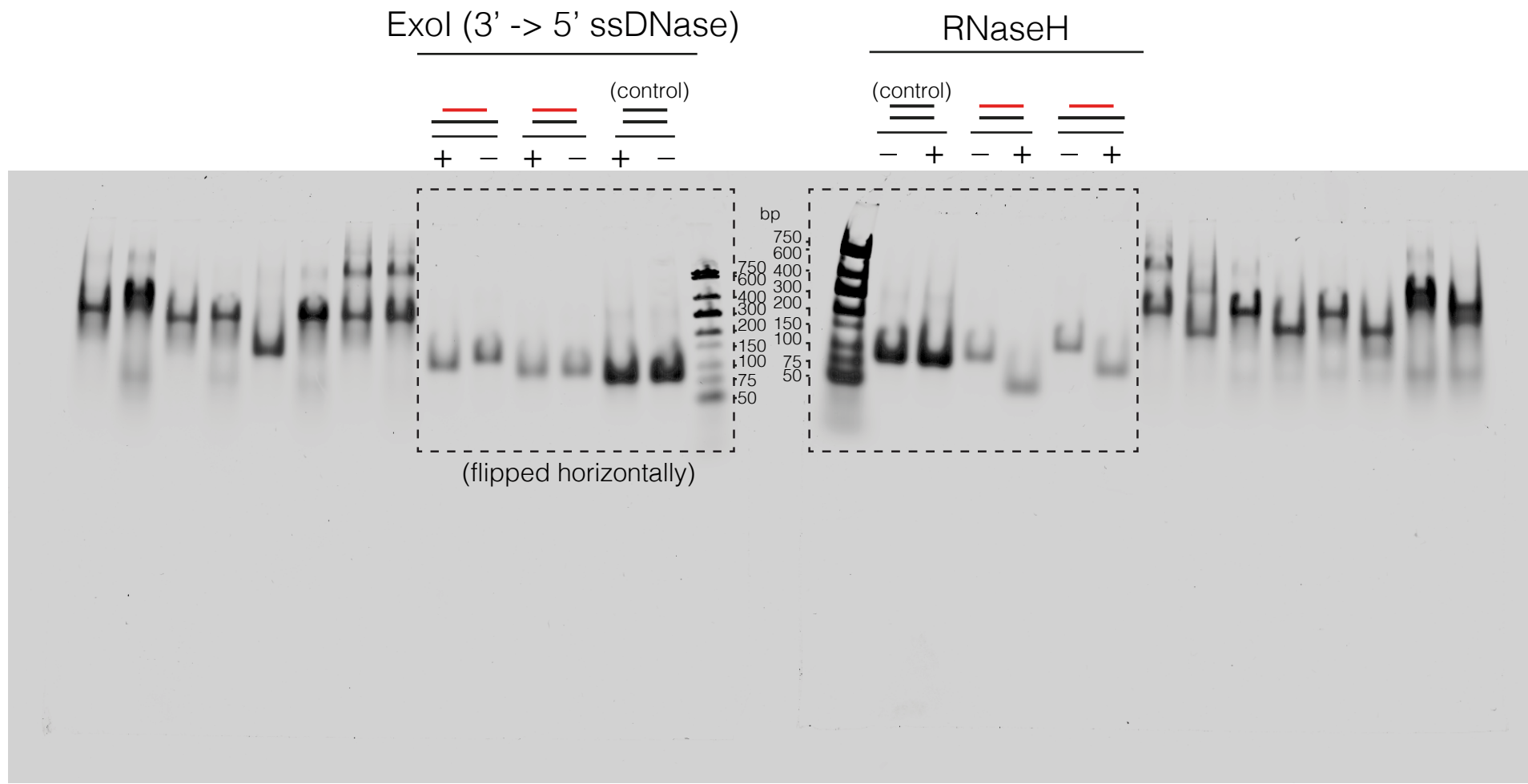

Ext. Data Fig 8c

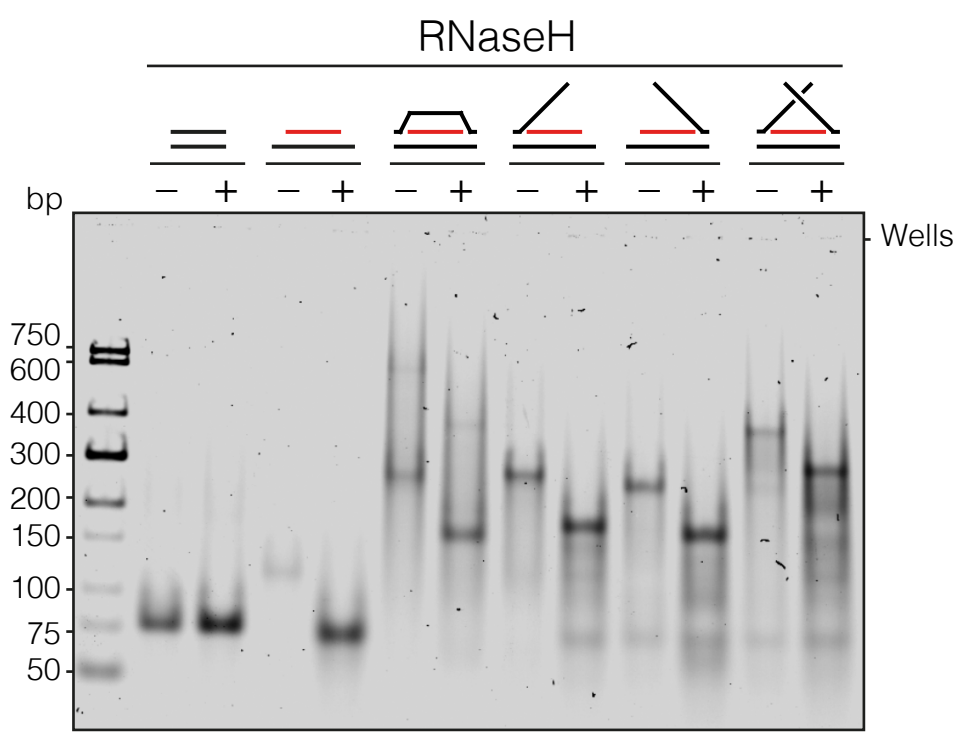

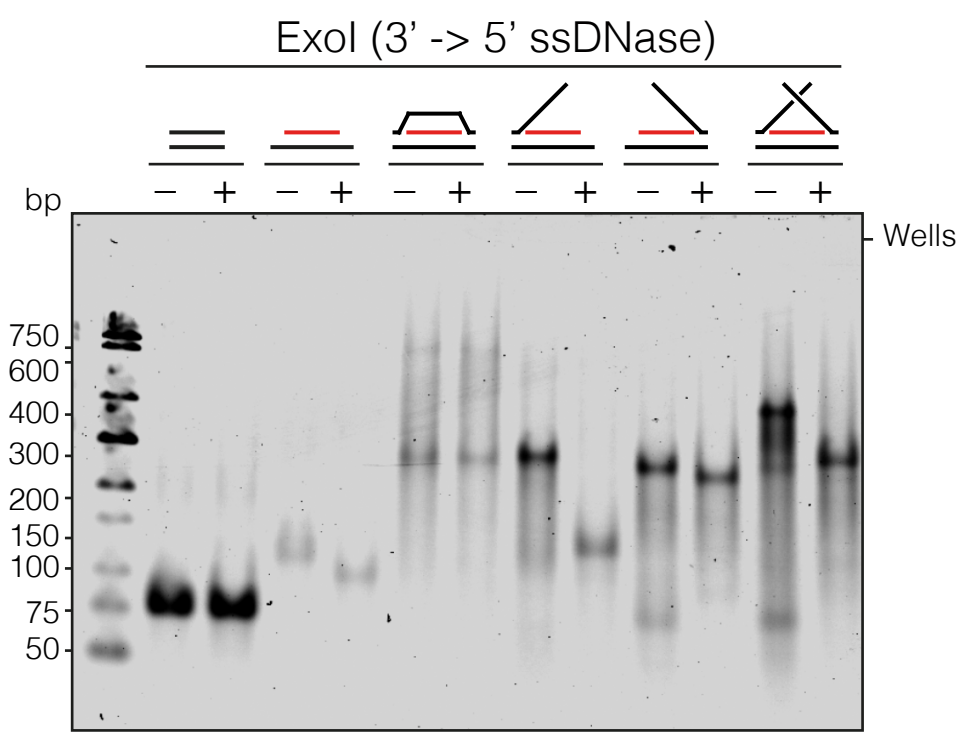

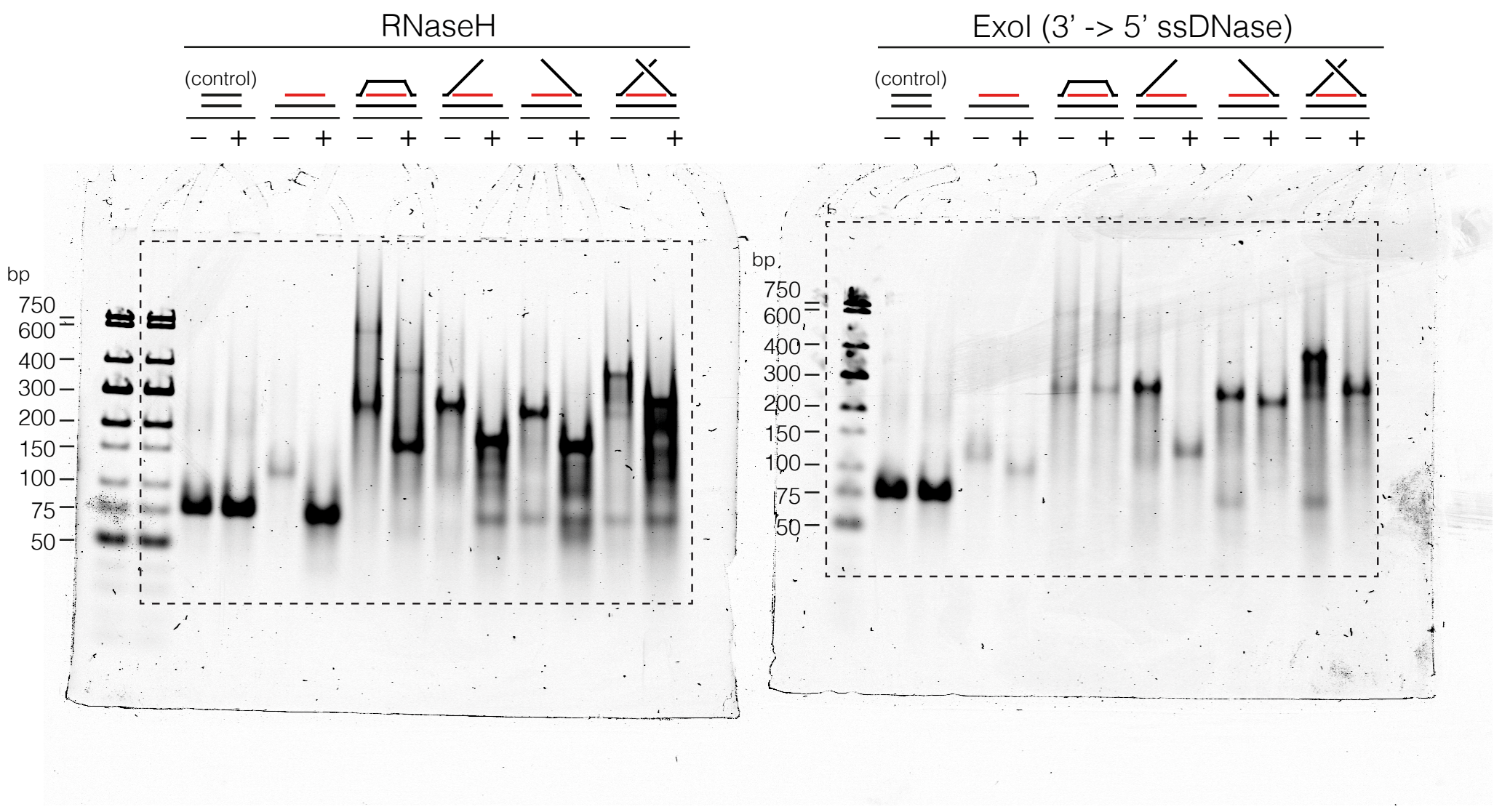

Supplement: Supplementary file 2 [file 41556_2026_2037_MOESM8_ESM.pdf]
